# Genome‐Wide Profiling of H3K27ac Identifies *TDO2* as a Pivotal Therapeutic Target in Metabolic Associated Steatohepatitis Liver Disease

**DOI:** 10.1002/advs.202404224

**Published:** 2024-10-04

**Authors:** Yaling Zhu, Limeng Shang, Yunshu Tang, Qiushuang Li, Lin Ding, Yi Wang, Tiantian Zhang, Bin Xie, Jinhu Ma, Xinyu Li, Shuwen Chen, Xinrui Yi, Jin Peng, Youfeng Liang, Anyuan He, Hong Yan, Huaqing Zhu, Buchun Zhang, Yong Zhu

**Affiliations:** ^1^ Department of Pathophysiology School of Basic Medical Sciences Anhui Medical University Hefei Anhui 230032 China; ^2^ Laboratory Animal Research Center School of Basic Medical Sciences Anhui Medical University Hefei Anhui 230032 China; ^3^ Department of Cardiology The First Affiliated Hospital of Anhui Medical University Hefei Anhui 230001 China; ^4^ School of Life Sciences Anhui Medical University Hefei Anhui 230032 China; ^5^ Department of Pathology The First Affiliated Hospital of USTC Division of Life Sciences and Medicine University of Science and Technology of China Hefei Anhui 230001 China; ^6^ Department of Pathology School of Basic Medical Sciences Southern Medical University Guangzhou Guangdong 510515 China; ^7^ Laboratory of Molecular Biology and Department of Biochemistry Anhui Medical University Hefei Anhui 230032 China; ^8^ Department of Cardiology The First Affiliated Hospital of USTC Division of Life Sciences and Medicine University of Science and Technology of China Hefei Anhui 230001 China

**Keywords:** H3K27ac, M1 polarization, MASLD, TDO2, YY1

## Abstract

H3K27ac has been widely recognized as a representative epigenetic marker of active enhancer, while its regulatory mechanisms in pathogenesis of metabolic dysfunction‐associated steatotic liver disease (MASLD) remain elusive. Here, a genome‐wide comparative study on H3K27ac activities and transcriptome profiling in high fat diet (HFD)‐induced MASLD model is performed. A significantly enhanced H3K27ac density with abundant alterations of regulatory transcriptome is observed in MASLD rats. Based on integrative analysis of ChIP‐Seq and RNA‐Seq, *TDO2* is identified as a critical contributor for abnormal lipid accumulation, transcriptionally activated by YY1‐promoted H3K27ac. Furthermore, *TDO2* depletion effectively protects against hepatic steatosis. In terms of mechanisms, *TDO2* activates NF‐κB pathway to promote macrophages M1 polarization, representing a crucial event in MASLD progression. A bovine serum albumin nanoparticle is fabricated to provide sustained release of Allopurinol (NPs‐Allo) for *TDO2* inhibition, possessing excellent biocompatibility and desired targeting capacity. Venous injection of NPs‐Allo robustly alleviates HFD‐induced metabolic disorders. This study reveals the pivotal role of *TDO2* and its underlying mechanisms in pathogenesis of MASLD epigenetically and genetically. Targeting H3K27ac‐*TDO2‐*NF‐κB axis may provide new insights into the pathogenesis of abnormal lipid accumulation and pave the way for developing novel strategies for MASLD prevention and treatment.

## Graphical abstract

A genome‐wide study in metabolic dysfunction‐associated steatotic liver disease (MASLD) uncovers the pivotal role of the active enhancer marker H3K27ac in modulating the key therapeutic target of *TDO2*, which triggers lipid accumulation and M1 macrophage polarization through NF‐κB activation. The use of BSA nanoparticles encapsulating Allopurinol for *TDO2* inhibition effectively mitigates metabolic disturbances, presenting a promising therapeutic strategy for MASLD.

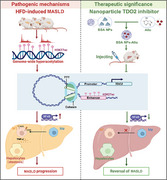

## 1 Introduction

Metabolic dysfunction‐associated steatotic liver disease (MASLD), also known as nonalcoholic fatty liver disease (NAFLD), is a growing epidemic chronic liver disease worldwide, which is characterized by lipid accumulation‐induced hepatocytes damage and innate immune cells‐activated chronic inflammation.^[^ ^1^ ^]^ Toxicity of excess lipids in hepatocytes promotes infiltration and activation of immune cells, resulting in inflammatory cascade.^[^ ^2^ ^]^ The combined effects of these fundamental cellular processes drive pathogenesis and progression of MASLD.^[^ ^3^ ^]^ Macrophage, as one of the most studied immune cells, has been widely reported to drive MASLD through polarizing to a proinflammatory phenotype (M1 polarization).^[^ ^4^ ^]^ Factors including overweight, insulin resistance, a sedentary lifestyle and an altered dietary pattern, as well as genetic factors and disturbances of the intestinal barrier function have been identified to impact on MASLD pathogenesis.^[^ ^5^ ^]^ Nevertheless, the high prevalence of MASLD cannot be entirely attributed to environmental factors or genetics alone. There is an increasing urgency to investigate the pivotal role of epigenetic factors in the development and progression of this disease, which are critical clinical challenges that have yet to be fully understood.

Epigenetics refers to heritable changes in gene expression without changing the underlying DNA sequence and can mediate crosstalk between genes and the environment.^[^ ^6^ ^]^ Histone modifications are epigenetic regulators of chromatin, which influence higher‐order chromatin structure by affecting contacts between different histones or/and between histones and DNA.^[^ ^7^ ^]^ Among them, H3K27ac (acetylation of the lysine residue at N‐terminal position 27 of the histone H3), a representative histone modification of active enhancer, has been proven to regulate gene expression by combining with transcription factors (TFs) and participate in the progression of various diseases, such as Alzheimer's disease^[^ ^8^ ^]^ and dilated cardiomyopathy.^[^ ^9^ ^]^ Besides, recent studies also implicated the role of H3K27ac in MASLD. For example, Liu *et al.* suggested that Snail1 prevents MASLD in obesity by modulating the deacetylation of H3K27 and suppressing fatty acid synthase expression.^[^ ^10^ ^]^ Liang *et al*. reported that homocysteine could increase H3K27ac and modulate steatosis.^[^ ^11^ ^]^ Nevertheless, the specific underlying mechanisms of H3K27ac in the occurrence and development of MASLD remain obscure.

*TDO2*, predominantly expressed in the liver, is a heme enzyme catalyzing tryptophan (TRP) to kynurenine (KYN), which is the first‐ and rate‐limiting step of the kynurenine pathway.^[^ ^12^ ^]^ Kynurenine pathway is mainly responsible for tryptophan metabolism, converting tryptophan to NAD^+^ along with intermediate products such as quinolinic acid and kynurenine.^[^ ^13^ ^]^ Aberrant *TDO2*‐induced dysfunctions of kynurenine pathway have been reported to be associated with multiple pathological processes, including cancer,^[^ ^14^ ^]^ autoimmune arthritis,^[^ ^15^ ^]^ and neurodegenerative disease.^[^ ^16^ ^]^ A pivotal oncogenic role of *TDO2* in the progression of HCC has been recognized recently.^[^ ^17^ ^]^ It was also reported that depletion of IDO1, the other isoenzyme that oxidize TRP to produce formylkynurenine and eventually degrade to KYN,^[^ ^18^ ^]^ aggravated atherosclerosis but not liver disease in MASH and atherosclerosis comorbidity mode, in which *TDO2* contributed to balancing the kynurenine pathway and inflammation.^[^ ^19^ ^]^ However, the functional significance and specific molecular mechanisms of *TDO2* in pathogenesis of MASLD are still largely undefined.

It is still challenging for clinical application of small‐molecule inhibitor due to their poor targeting ability, low potency and adverse events.^[^ ^20^ ^]^ Nanotechnology ‐ involved drug delivery is emerging with great potential for its capacity to enhance therapeutic efficacy while reducing systemic side effects.^[^ ^21^ ^]^ Protein‐based carriers such as bovine serum albumin (BSA) displayed an intriguing potential to be utilized in nanoformulation.^[^ ^22^ ^]^ Albumin has even been approved by Food and Drug Administration (FDA) as a nano‐drug carrier for its various advantages.^[^ ^23^ ^]^ Therefore, the development of high‐efficiency protein‐based nanoparticles guides the way for precise and safe delivery of selective inhibitors.

In the present study, we investigated genome‐wide patterns of enhancer‐target H3K27ac ChIP‐Seq and high‐throughput RNA‐Seq in the liver of MASLD rats, aiming to identify the critical disease‐associated targets. Interestingly, we found *TDO2*, which was transcriptionally activated by YY1‐mediated H3K27ac, was remarkably upregulated in MASLD. Ablation of *TDO2* notably alleviated hepatic steatosis both in vitro and in vivo. Furthermore, *TDO2* shifted macrophage towards M1 polarization via activation of KYN/AHR/NF‐κB signaling pathway, thus accelerating hepatic steatosis. A TDO2 inhibitor‐loaded BSA nanoparticles (NPs‐Allo) exhibited excellent therapeutic effects in HFD‐fed rats, representing a promising strategy for MASLD treatment.

## 2 Results

### 2.1 H3K27ac Is a Representative Histone Marker in Regulation of Key Genes in MASLD

To investigate epigenetic regulation of gene expression in the pathogenesis of MASLD, we first utilized ChIP‐Seq data of healthy and MASLD patients (GSE112221) to establish genome‐wide patterns of histone modification markers, including H3K4me3 (active promoters), H3K27ac (active enhancers), and H3K4me1 (active/poised enhancers). Notably, it was remarkably distinguished by H3K27ac between normal and patients (Figure S1A,B, Supporting Information), suggesting a pivotal role acted by H3K27ac in regulation of the disease states. Subsequently, we integrated RNA‐Seq data from human, rat, and mouse samples, identifying 256 overlapping genes across species, with 121 genes consistently upregulated (uniform genes) in MASLD‐affected samples involved in lipid metabolic processes (Figure S1C and Table S5, Supporting Information). We further analyzed the peak heatmap of these uniform genes, normalized across all histone samples (Figure S1D,E and Table S6, Supporting Information), confirming that samples primarily grouped by disease status in the H3K27ac marker. This supports the critical role of H3K27ac in regulating these uniform genes in MASLD development across species.

To elucidate the molecular mechanism of H3K27ac in regulating these uniform genes in MASLD, we established HFD‐induced MASLD model in rats (Figure S2A, Supporting Information). The liver of HFD‐induced rats sequentially becomes larger, softer and yellower during the modeling process (Figure S3A, Supporting Information). Histological analyses demonstrated typical signs of MASLD, including extensive lipid accumulation (Figure S3B,C, Supporting Information), ultrastructural changes in hepatocytes (Figure S3D, Supporting Information) of HFD‐fed rats, with increased body weight and liver index (Figure S3E,F, Supporting Information). Biochemical analyses indicated the altered serum lipid (Figure S3G–L, Supporting Information) and elevated inflammatory profiles (Figure S3M–O, Supporting Information) in MASLD rats. These findings validated the successful establishment of the HFD‐induced MASLD model in rats.

**Figure 1:**
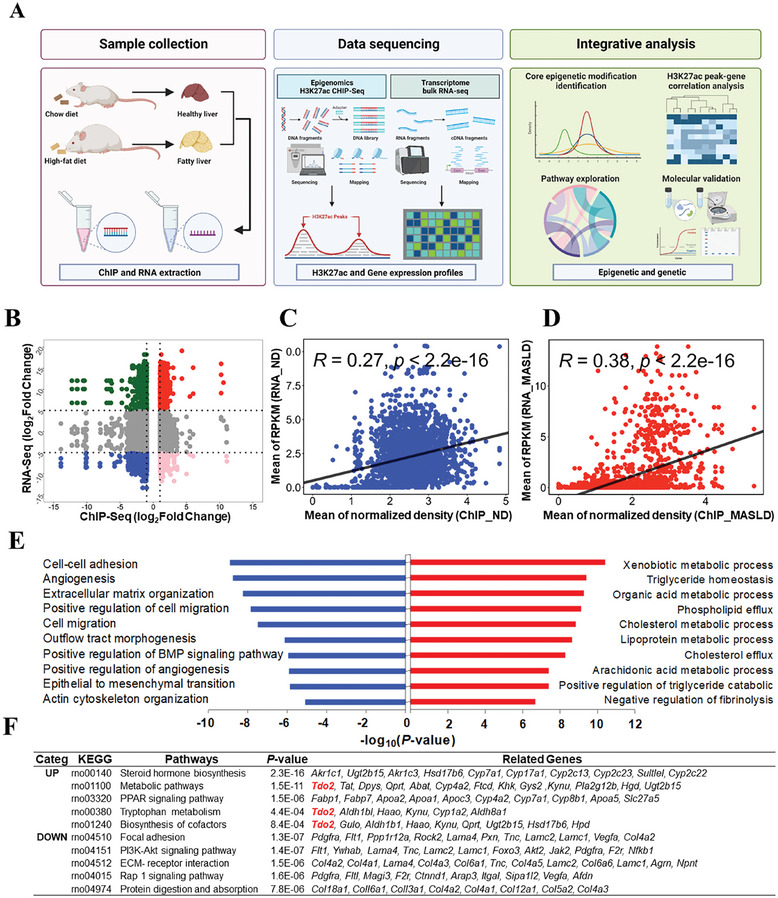
HFD induced robust H3K27ac‐marked enhancer aberrations in lipid metabolism genes. A) Schematic diagram of the strategy to identify core epigenetic and genetic biomarkers in MASLD progression. B) Genome‐wide “four‐way” plot showed the genes with a threshold of log_2_foldchange (|ChIP| >1 and |RNA| >5), which were generated by integrated analysis of ChIP‐Seq and RNA‐Seq between ND and MASLD groups. PP peak‐genes with positive upregulation between H3K27ac peaks and genes, which were colored red (log_2_foldchange (ChIP > 1 and RNA > 5), NN peak‐genes with downregulation between H3K27ac peaks and genes, which were colored blue (log_2_foldchange (ChIP < −1 and RNA < −5), PN peak‐genes with positive regulation but negative expression, which were colored pink (log_2_foldchange (ChIP > 1 and RNA < −5), NP peak‐genes with negative regulation but positive expression, which were colored green. C) The positive correlation of H3K27ac peaks of ChIP‐Seq and gene expression of RNA‐Seq in ND group. D) The positive correlation of H3K27ac peaks of ChIP‐Seq and gene expression of RNA‐Seq in MASLD group. E) Over‐representative of biological processes of putative peak target‐genes of PP and NN. F) Top KEGG pathways of putative target‐genes of PP and NN by adopting DAVID (https://david‐d.ncifcrf.gov/). ND: Normal Diet; MASLD: Metabolic Associated Steatohepatitis Liver Disease. The correlation coefficients (*R* values) and *p*‐values were calculated by Spearman analysis.

Subsequently, an integrative analysis of H3K27ac ChIP‐Seq and RNA‐Seq data was conducted to identify differential H3K27ac peaks and their target genes in the pathological process of MASLD (**Figure** **1**A). First, we computed the Pearson correlation coefficient (PCC) between differential H3K27ac peaks and their potential target genes whose transcription start sites (TSSs) were positioned within a 1000‐kb window of the peaks referring to the methodology detailed in Hongbo et al.^[^ ^24^ ^]^ Notably, a total of 3607 differential peak‐gene correlations of both positive and negative with a threshold of log_2_foldchange (|ChIP| > 1 and |RNA| > 5), including 400 PP peak‐genes (log_2_foldchange (ChIP > 1 and RNA > 5), 1918 NN peak‐genes (log_2_foldchange (ChIP < −1 and RNA < −5), 756 PN peak‐genes (log_2_foldchange (ChIP >1 and RNA < −5), and 533 NP peak‐genes (log_2_foldchange (ChIP < −1 and RNA > 5) (Figure 1B). More interestingly, we found that genes and peaks were not one‐on‐one relationship (Figure S4, Supporting Information), which is consistent with previous study that multiple enhancers may be used to regulate a gene and various genes may be contributed by one peak.^[^ ^25^ ^]^ Besides, the correlation of ChIP‐Seq and RNA‐Seq in Normal Diet (ND) (*R* = 0.27, *p* < 2.2 × 10^−16^, NN peak‐genes) and MASLD (*R* = 0.38, *p* < 2.2 × 10^−16^, PP peak‐genes) group are shown in Figure 1C,D, which suggested various genes were mediated by H3K27ac in both groups. Then we performed functional enrichment analysis of differential H3K27ac peak‐genes of PP and NN to better evaluate the function alteration by HFD‐induced MASLD. Of interest, the differentially hyper‐acetylated peak‐genes between normal and MASLD livers were significantly enriched in lipid metabolic processes including “triglyceride homeostasis,” “cholesterol metabolic process,” and “lipoprotein metabolic process” using DAVID database (Figure 1E). And for KEGG pathways, “steroid hormone biosynthesis,” “metabolic pathways,” and “PPAR signaling pathway” were also discerned (Figure 1F). Taken together, these results not only revealed robust alterations of H3K27ac‐marked enhancers and transcriptome variations in livers of MASLD rats induced by HFD, but also further emphasized the critical role of H3K27ac in the regulation of key genes by dysregulating lipid metabolic processes in MASLD pathogenesis.

### 2.2 *TDO2* Is Epigenetically Activated by H3K27ac Modification in MASLD

**Figure 2:**
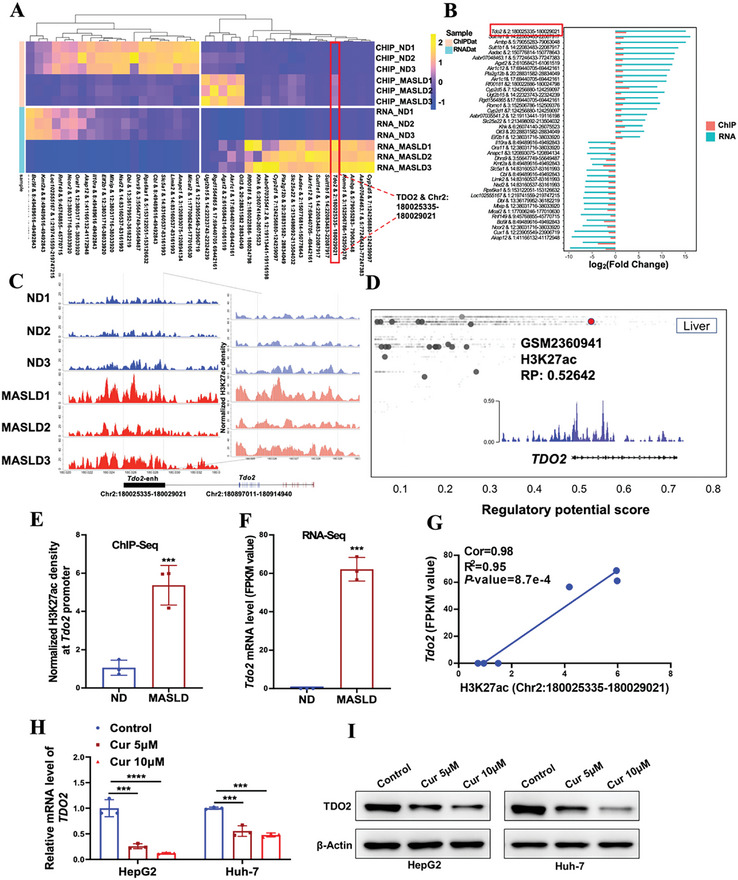
*Tdo2* is a pivotal factor epigenetically activated by H3K27ac in MASLD. A) Heatmap of ChIP‐Seq and RNA‐Seq data showing differential H3K27ac enrichment and transcriptional level between ND and MASLD group, of which *Tdo2 &* Chr2: 180025335‐180029021 was significantly over‐represented. B) The barplot of differential fold change of top 20 H3K27ac peak‐genes in ChIP‐Seq and RNA‐Seq. C) The differential density of H3K27 acetylation on *Tdo2* between ND and MASLD rats (Rat: Rnor_6.0_ensembl_104). D) H3K27ac was a histone modification marker of *TDO2* gene in the liver according to the Cistrome database (accession number: GSM2360941). All Cistrome data have been carefully curated and processed with a streamlined analysis pipeline and evaluated with comprehensive quality control metrics. E) The H3K27ac density of *Tdo2* gene in MASLD rats compared with ND rats using ChIP‐Seq data (*n* = 3 per group; unpaired two‐sided Student *t*‐test). F) The mRNA expression level of *Tdo2* in MASLD rats relative to ND individuals (*n* = 3 per group; unpaired two‐sided Student *t*‐test). G) Correlation between *Tdo2* expression level and H3K27ac peak (chr2:180025335‐180029021) density (*n* = 6, Cor = 0.98, R2 = 0.95). H,I) The mRNA and protein levels of *TDO2* in HepG2 and Huh‐7 cells treated with indicated concentrations of Curcumin (Cur) were determined by qRT‐PCR and Western blotting, respectively. *P*‐values were calculated with one‐way ANOVA test (*n* = 3 per group). Results were shown as mean ± SD. *P*‐values are indicated by * < 0.05; ** < 0.01; *** < 0.001; **** < 0.0001.

Next, we sought to investigate the key genes and pathways overrepresented by positive correlated H3K27ac peak‐gene between normal and MASLD rats. Intriguingly, *Tdo2* was significantly screened out as the most distinguished disease‐associated H3K27ac peak‐genes with Correlation = 0.98 and *p*‐value = 8.7 × 10^−4^ (**Figure** **2**A–G; Table S4, Supporting Information), and multiple enriched in metabolic pathways (Figure 1F), as well as one of significant uniform genes induced by H3K27ac among different species (Figure S1C and Table S5, Supporting Information). Notably, we further validated a higher density of H3K27 acetylation at the peak region of Chr2: 180025335‐180029021 in MASLD group, which was defined as putative enhancer region of *Tdo2* (Chr2: 180897011‐180914940) (Figure 2C), confirmed by Cistrome database (accession number: GSM2360941) validation in human liver tissues (Figure 2D). Pearson correlation analysis also revealed a positive correlation between *Tdo2* and H3K27ac in MASLD (Figure 2E–G). In addition, Curcumin (an inhibitor of H3K27ac)‐induced decrease in *TDO2* mRNA and protein levels were observed in a dose‐dependent manner (Figure 2H,I), further confirming that H3K27ac enhancers were important regulatory elements that regulated the hub target gene of *TDO2* in the pathological process of MASLD. Besides, we were surprised to observe that the expression of *TDO2* in the liver was significantly higher than that in other tissues in humans, rats, and mice (Figure S5A–C, Supporting Information). RNA single cell type specificity analysis showed that among various cells in the liver, hepatocytes accounted for the majority of *TDO2* cell localization (Figure S5D–F, Supporting Information). Therefore, *TDO2*, which characterized by the expression specificity to aggregate in hepatocytes was upregulated in MASLD upon the epigenetic regulation of H3K27ac, potentially manifested a pivotal role in pathophysiology of the liver.

### 2.3 *YY1* Promoted Transcriptional Activation of *TDO2* via Enhancing H3K27ac Modification

The mechanism underlying the activated H3K27ac modification of *TDO2* in MASLD model was further investigated. We firstly predicted the TFs binding with the enhancer and promoter of *TDO2* based on UCSC (http://genome‐asia.ucsc.edu) and PROMO (http://alggen.lsi.upc.es/) database (Figure S6, Supporting Information). Top 20 overlapped TFs related to both promoter and enhancer of *TDO2* are presented in Figure S7A (Supporting Information), among which Yin Yang 1 (*YY1*) was screened out as the most related TF, which was also confirmed by our PPI network analysis (Figure S7B, Supporting Information).

To further investigate the conservation of *YY1* and *TDO2* among human, mouse, and rat, homology analysis displayed high sequence consensus of *TDO2* and *YY1* by using Uniprot database (https://www.uniprot.org/) (Figure S7C,D, Supporting Information). Totally, 45 overlapped TFs were identified to potentially target both promoter and enhancer of *TDO2* in three different species (Figure S7E, Supporting Information), and *YY1* was also pointed out as the most relevant TF for its core status of widespread interaction with other TFs (Figure S7F, Supporting Information). Moreover, Cistrome database also validated that *YY1* acted as a potential transcriptional regulator of *TDO2* in liver tissue (Figure S7G, Supporting Information).

**Figure 3:**
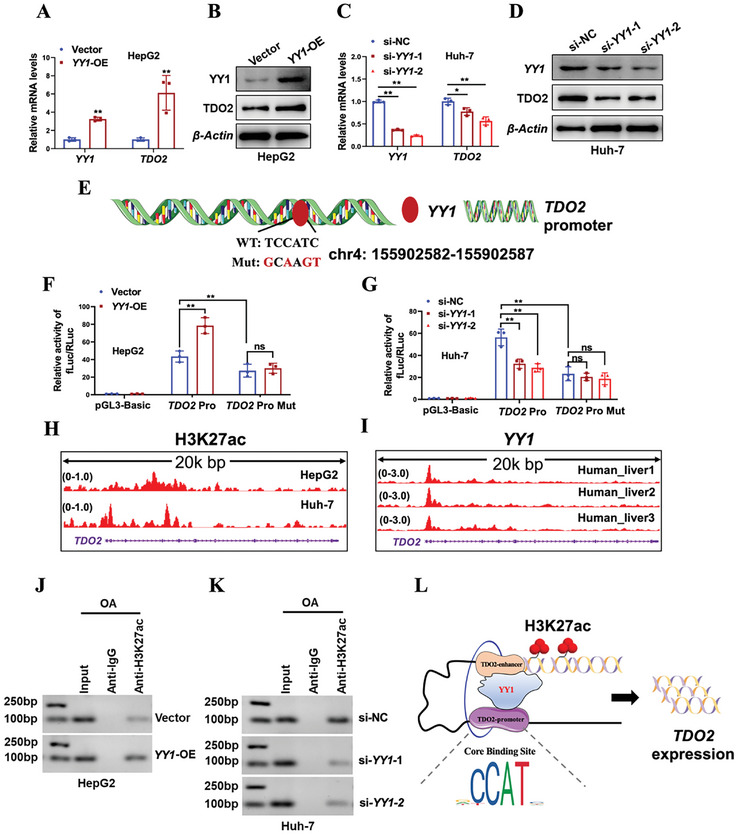
*YY1* induced *TDO2* upregulation via modulating H3K27ac. A,B) The mRNA and protein levels of YY1 and TDO2 in OA (0.6 × 10^−3^ m)‐induced HepG2 cells transfected with *YY1* overexpression plasmid (*YY1*‐OE) or empty vector (Vector) were examined by qRT‐PCR and Western blotting, respectively. *P*‐values were calculated with unpaired two‐sided Student *t*‐test (*n* = 3 per group). C,D) The mRNA and protein levels of YY1 and TDO2 in OA (0.6 × 10^−3^ m)‐induced Huh‐7 cells transfected with siRNAs against *YY1* (si‐*YY1*‐1 and si‐*YY1*‐2) or negative control siRNA (si‐NC) were determined by qRT‐PCR and Western blotting, respectively. *P*‐values were calculated with one‐way ANOVA test (*n* = 3 per group). E) Schematic representation of the predicted wild‐type and mutant *YY1* binding sites in the DNA promoter region of *TDO2* based on Jaspar (https://jaspar.genereg.net/). F,G) Regulation of wild‐type or mutant *TDO2* promoter activities by *YY1* was determined by luciferase reporter assay. Renilla luciferase activity as input control (*n* = 3 per group, unpaired two‐sided Student *t*‐test in (F) and one‐way ANOVA test in (G). H) ChIP‐Seq data shows the enrichments of H3K27ac around the promoter region of *TDO2* in HepG2 and Huh‐7 cells according to the Cistrome database (accession number: GSM1670897, GSM2360939). I) ChIP‐Seq data shows the enrichments of *YY1* around the promoter region of *TDO2* in human liver according to the Cistrome database (accession number: ENCSR382MOM_1, ENCSR994YLZ_2, ENCSR994YLZ_1). J,K) Regulation of enrichments of H3K27ac around the promoter region of *TDO2* in HepG2 and Huh‐7 cells by *YY1* was determined by ChIP assay. L) Schematic diagram shows an interactive model that *YY1* transcriptionally activated *TDO2* expression. Results were shown as mean ± SD. *P*‐values are indicated by * < 0.05; ** < 0.01; *** < 0.001; ns: not significant.

A series of experiments were subsequently carried out to verify our predicted results by bioinformatics. The mRNA and protein levels of *YY1* and *TDO2* were first confirmed to be upregulated in *YY1*‐overexpressing HepG2 cells with treatment of OA (Oleic acid) (**Figure** **3**A,B). In contrast, it was observed that the expression of *YY1* and *TDO2* were reduced in *YY1*‐depleted Huh‐7 cells treated with OA, both at RNA and protein levels (Figure 3C,D). Furthermore, the promoter sequence of *TDO2* (2 kb) containing potential wild‐type or mutant *YY1*‐binding sites was cloned into the pGL3‐reporter plasmid (Figure 3E), wild‐type *TDO2* promoter‐derived reporter activity was dependent on *YY1*, but mutation of *YY1*‐binding sites abolished the influence of *YY1* on *TDO2* promoter‐derived reporter activity (Figure 3F,G). Besides, ChIP‐Seq data from the Cistrome database suggested the high H3K27ac enrichments in *TDO2* gene region in HepG2 and Huh‐7 cells (Figure 3H), obvious binding peaks of *YY1* were also observed at the promoter region of *TDO2* in human liver tissues (Figure 3I), which implied *TDO2* was transcriptionally regulated by both *YY1* and H3K27ac. Interestingly, ChIP assay showed increased H3K27ac level of *TDO2* in HepG2 cells with forced expression of *YY1* (Figure 3J), while a decreased enrichment of H3K27ac was observed in Huh‐7 cells upon *YY1* depletion (Figure 3K). Taken together, these data highlighted YY1's critical role in promoting transcriptional activation of *TDO2* via enhancing H3K27ac modification (Figure 3L).

### 2.4 Elevated *TDO2* Is Observed in Hepatic Steatosis Patients and Hepatocellular Steatosis Model

**Figure 4:**
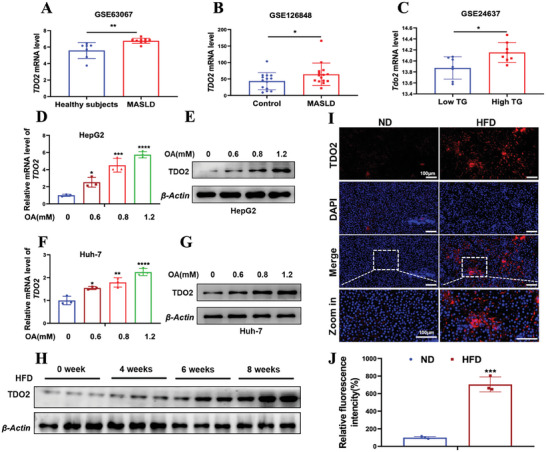
*TDO2* expression was increased in hepatic steatosis patients and models. A,B) *TDO2* mRNA expression in the liver of MASLD and health patients from GSE63067 (Healthy subjects *n* = 7, MASLD *n* = 9, unpaired two‐sided Student *t*‐test) and GSE126848 (Control *n* = 14, MASLD *n* = 15, unpaired two‐sided Student *t*‐test). C) The mRNA level of *Tdo2* in the liver of mice with low serum TG and high serum TG from GSE34637 (*n* = 8 per group, unpaired two‐sided Student *t*‐test). D–G) The mRNA and protein level of TDO2 in HepG2 and Huh‐7 cells treated with indicated dose of OA for 48 h were examined by qRT‐PCR and Western blotting, respectively. *P*‐values were calculated using one‐way ANOVA test (*n* = 3 per group). H) Protein level of TDO2 in the liver tissues of rats fed with HFD for indicated weeks was evaluated by Western blotting (*n* = 3 per group). I,J) Representative immunofluorescence staining and quantification of TDO2 expression in liver tissue sections of rats fed with ND or HFD (*n* = 3 per group, unpaired two‐sided Student *t*‐test). Scale bar, 100 µm. Results in (D) and (F) are shown as mean ± SD. *P*‐values are indicated by * < 0.05; ** < 0.01; *** < 0.001; **** < 0.0001.

To investigate the functional role of *TDO2* in hepatocellular steatosis, we first examined its expression pattern in MASLD. Analysis of GEO data revealed elevated *TDO2* expression in the liver tissues of patients with MASLD compared to normal healthy subjects (**Figure** **4**A,B), increased expression level of *Tdo2* in the liver tissues of mice with high TG level was observed compared with counterparts with low TG level (Figure 4C). Further, *TDO2* expression was induced in HepG2 and Huh‐7 cells by OA in a dose‐dependent manner (Figure 4D–G). Consistent with the results of cellular steatosis model in vitro, rats fed with HFD also exhibited higher hepatic *TDO2* expression, as compared with ND‐fed rats (Figure 4H–J). Collectively, these findings suggest that *TDO2* upregulation is associated with hepatic steatosis, supporting its potential role as a biomarker or therapeutic target in the context of MASLD.

### 2.5 Knockdown of *Tdo2* Protects against Hepatic Steatosis in HFD‐Induced Rats Model

**Figure 5:**
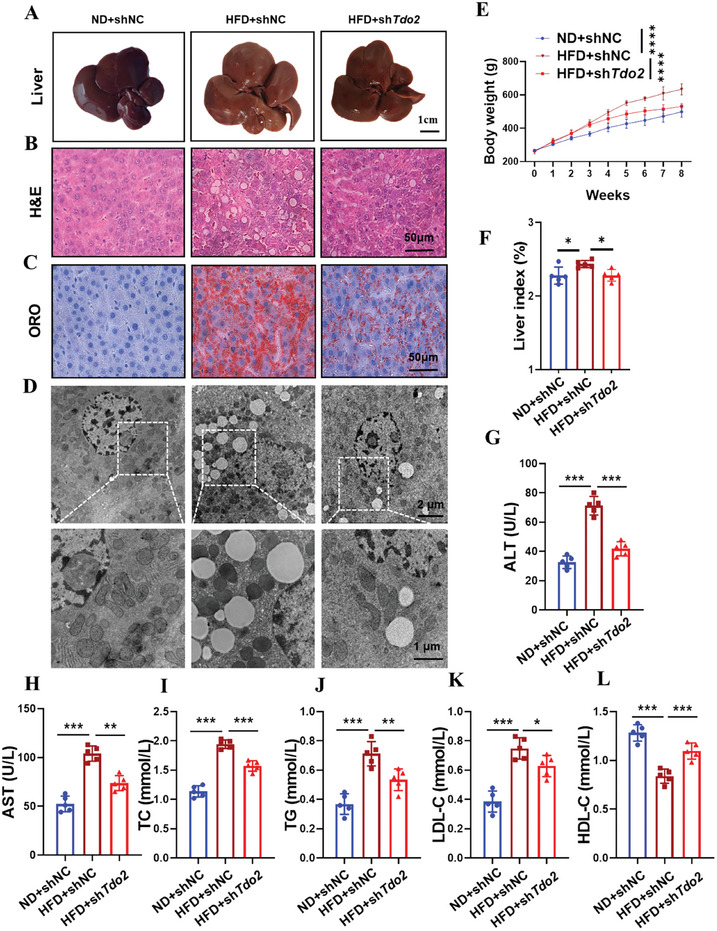
Ablation of *Tdo2* ameliorated HFD‐induced hepatic steatosis in rats. Rats were infected with lentivirus particles of shNC or sh*Tdo2* through tail‐vein injection and fed with ND or HFD. A) Representative captured liver tissues (*n* = 3 per group). scale bar, 1 cm. B,C) H&E and Oil Red O staining of liver tissue sections (*n* = 3 per group). Scale bar, 50 µm. D) The ultrastructure of transmission electron microscope of liver tissue sections at 3600× (left, scale bar, 2 µm) and 8500× (right, scale bar, 1 µm) magnification (*n* = 3 per group). E) Body weight was measured every week from 0 to 8 weeks (*n* = 5 per group). F) Liver index was measured after sacrifice of rats in each group (*n* = 5 per group). G–L) Serum levels of a ALT, AST, TC, TG, LDL‐C, and HDL‐C of rats in each group (*n* = 5 per group). Results are shown as mean ± SD. *P*‐values are indicated by * < 0.05; ** < 0.01; *** < 0.001; **** < 0.0001 (two‐way ANOVA test in (E), others with one‐way ANOVA test). Blue: ND+shNC; Dark red: HFD+shNC; Red: HFD+sh*Tdo2*. ALT: Alanine aminotransferase; AST: Aspartate aminotransferase; TC: Total cholesterol; TG: Total triglycerides; LDL‐C: Low‐density lipoprotein cholesterol; HDL‐C: High‐density lipoprotein cholesterol.

To further determine the pathological role of *Tdo2* in hepatic lipid accumulation, lentivirus‐carrying shRNAs against *Tdo2* (sh*Tdo2*) was injected into HFD‐induced rats via tail vein (Figure S2B, Supporting Information). The elevated TDO2 expression in liver tissues of HFD‐induced rats was markedly decreased following sh*Tdo2* lentivirus administration (Figure S8A–C, Supporting Information). Functionally, the appearance of the liver changed notably from HFD‐induced light yellow color with thicker edges and greasy surface to dark red hue with sharper edges and a smoother surface upon *Tdo2* suppression (**Figure** **5**A). Moreover, severe steatosis and lipid accumulation in livers of HFD‐induced rats were remarkably diminished by sh*Tdo2* injection, as evidenced by H&E staining (Figure 5B), Oil Red O staining (Figure 5C), and transmission electron microscopy (Figure 5D). In addition, *Tdo2* knockdown resulted in noticeable reductions in both body weight and liver index gain in HFD‐fed rats (Figure 5E,F). Furthermore, the increased serum levels of ALT, AST, TC, TG, LDL‐C and decreased serum HDL‐C levels of HFD‐induced rats were notably reverted by *Tdo2* depletion (Figure 5G–L). Thus, loss of hepatic *Tdo2* alleviated hepatic steatosis in HFD‐fed rats.

### 2.6 *TDO2* Promotes M1 Polarization of Macrophages in Hepatic Steatosis Models

**Figure 6:**
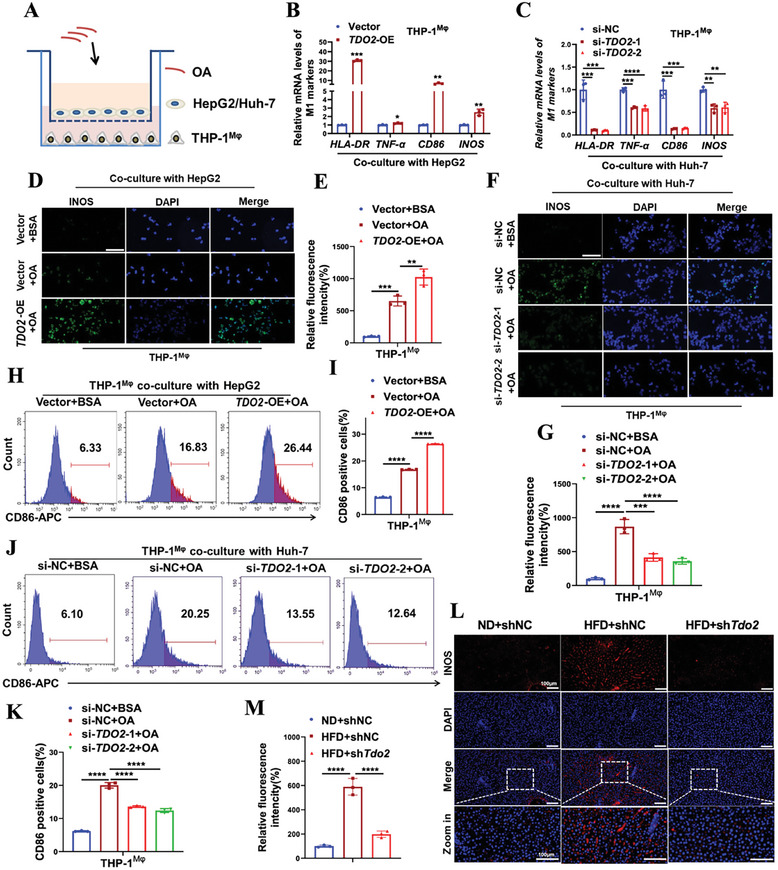
*TDO2* contributed to M1 polarization of macrophages in hepatic steatosis models. A) Schematic diagram depicts the co‐culture system. OA‐induced HepG2/Huh‐7 cells transfected with *TDO2* over‐expression/siRNAs or their respective negative control were seeded into the upper chamber of a transwell, THP‐1‐derived macrophages were seeded into the bottom chamber. B,C) The mRNA levels of M1 markers (*HLA‐DR* and *TNF‐α*) in macrophages co‐cultured with *TDO2*‐overexpressing HepG2 and *TDO2*‐depleting Huh‐7 cells with OA treatment (0.6 × 10^−3^ m) were determined by qRT‐PCR (*n* = 3 per group, unpaired two‐sided Student *t*‐test in (B) and one‐way ANOVA test in (C)). D–G) Representative immunofluorescence staining and quantification of iNOS expression in macrophages co‐cultured with *TDO2*‐overexpressing HepG2 and *TDO2*‐depleting Huh‐7 cells with OA treatment (0.6 × 10^−3^ m, scale bar, 100 µm). *P*‐values were calculated with one‐way ANOVA test (*n* = 3 per group). H–K) The percentage of M1 (CD86^+^) macrophages in THP‐1‐derived macrophages co‐cultured with *TDO2*‐overexpressing HepG2 and *TDO2*‐depleting Huh‐7 cells with OA treatment (0.6 × 10^−3^ m) was measured by flow cytometry. *P*‐values were calculated with one‐way ANOVA test (*n* = 3 per group). L,M) Representative immunofluorescence staining and quantification of iNOS expression in liver tissue sections of rats infected with lentivirus particles of shNC or sh*Tdo2* through tail‐vein injection and fed with ND or HFD (*n* = 3 per group, one‐way ANOVA test). Scale bar, 100 µm. Results were shown as mean ± SD. *P*‐values are indicated by * < 0.05; ** < 0.01; *** < 0.001; **** < 0.0001 (unpaired two‐tailed Student *t*‐test).

It has been reported that induction of inflammatory M1 macrophage polarization facilitated the development of MASLD.^[^ ^4^ ^]^ To explore whether *TDO2* promoted lipid accumulation by shifting polarization of macrophages, a co‐culture system was established using OA‐induced HepG2/Huh‐7 cells and THP‐1‐derived macrophages (**Figure** **6**A). Given the lower endogenous TDO2 level in HepG2 cells and higher endogenous TDO2 level in Huh‐7 cells (Figure S9, Supporting Information), *TDO2* was thereby overexpressed in HepG2 cells and depleted in Huh‐7 cells for subsequent functional and mechanistic investigation. As expected, qRT‐PCR analysis showed overexpression of *TDO2* in HepG2 cells significantly enhanced the expressions of M1 markers (*HLA‐DR* and *TNF‐α*) in the co‐cultured macrophages with OA induction (Figure 6B), while Huh‐7 cells transfected with *TDO2* siRNAs exhibited an opposite effect on expressions of M1 markers (Figure 6C). The protein level of iNOS in macrophages was enhanced when co‐cultured with *TDO2*‐overexpressing HepG2 cells with OA supplement (Figure 6D,E), but was inhibited when co‐cultured with *TDO2*‐depleting Huh‐7 cells (Figure 6F,G), as determined by immunofluorescence staining. Flow cytometric analysis revealed that forcing expression of *TDO2* in HepG2 cells further boosted the increased percentage of CD86^+^ macrophages due to OA treatment (Figure 6H,I), whereas knockdown of *TDO2* in Huh‐7 cells reduced OA‐induced increased percentage of CD86^+^ macrophages (Figure 6J,K). Furthermore, ablation of *TDO2* remarkably suppressed the increased expression of iNOS in the liver tissue sections of rats fed with HFD (Figure 6L,M).

Next, we focused on the hepatocytes seeded into the upper chamber and explored the effects of *TDO2* on OA‐induced hepatic steatosis in HepG2 and Huh‐7 cells. Both mRNA and protein levels of *TDO2* were obviously increased upon *TDO2* overexpression plasmid transfection in HepG2 cells (Figure S10A,B, Supporting Information). As expected, *TDO2* overexpression significantly promoted lipid accumulation in OA‐treated HepG2 cells as determined by Oil Red O staining assay (Figure S10C,D, Supporting Information) and Bodipy 493/503 staining assay (Figure S10E,F, Supporting Information). There was an apparent increase in intracellular TG level in *TDO2*‐overexpressing HepG2 cells (Figure S10G, Supporting Information). Overexpression expression of *TDO2* obviously increased OA‐induced upregulation of lipid transport and synthesis genes (*ACC1, SCD1, CD36*, and *PPARG*) in HepG2 cells (Figure S10H, Supporting Information). Conversely, *TDO2* was silenced in OA‐induced Huh‐7 cells (Figure S10I,J, Supporting Information). Increased lipid droplet accumulation in OA‐treated Huh‐7 cells was significantly impaired due to *TDO2* depletion as examined by Oil Red O staining assay (Figure S10K,L, Supporting Information) and Bodipy 493/503 staining assay (Figure S10M,N, Supporting Information). An obvious decrease in intracellular TG level was observed in *TDO2*‐silent Huh‐7 cells (Figure S10O, Supporting Information). Knockdown of *TDO2* markedly diminished OA‐induced upregulation of lipid transport and synthesis genes (*ACC1, SCD1, CD36*, and *PPARG*) in Huh‐7 cells (Figure S10P, Supporting Information). Collectively, these results suggested hepatic *TDO2* promoted macrophages towards M1 polarization to accelerate hepatic steatosis both in vitro and in vivo.

### 2.7 *TDO2* Activates NF‐κB Pathway to Promote Macrophages M1 Polarization

**Figure 7:**
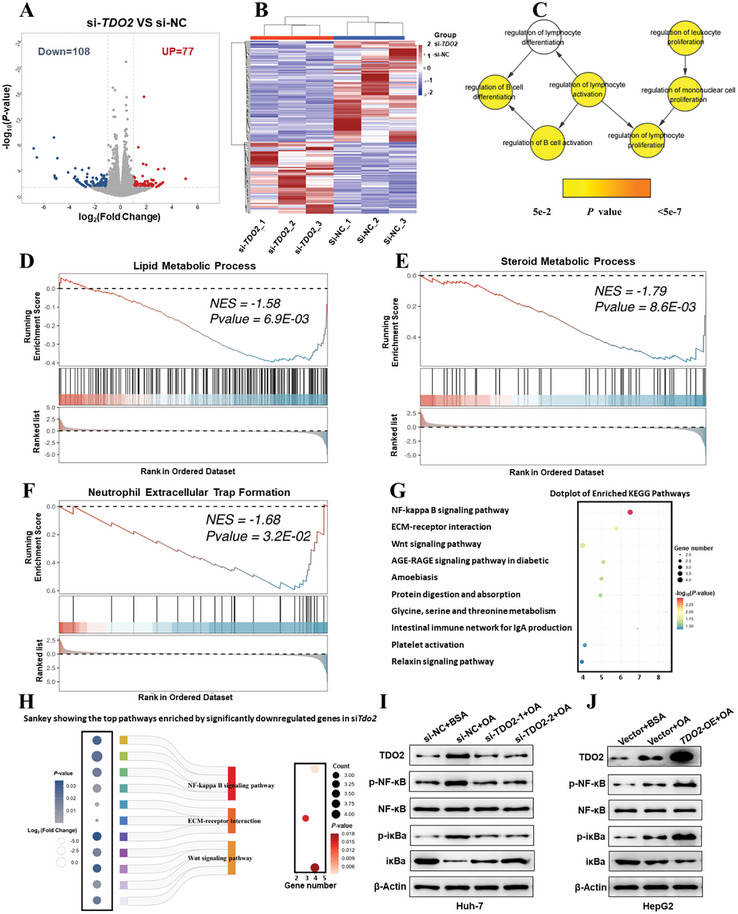
*TDO2* promotes M1 polarization of macrophages by activating the NF‐κB pathway. A) Volcano plot of remarkable differentially expressed genes between si*TDO2* and siNC groups. B) Transcription profiles of differentially expressed genes between si*TDO2* and siNC groups. C) Inflammatory response network of over‐representative GO terms of DEGs. The size and color of node represented gene number and *p*‐value, respectively. D–F) Gene Set Enrichment Analysis (GSEA) of differential expressing genes in *TDO2*‐depleted Huh‐7 cells in the co‐culture system. G) Dot plot of enriched KEGG terms. H) Sankey plot showing top three pathways enriched by significantly downregulated genes in si*TDO2* group. Left column shows the *p* value of enriched pathway, where circle size represents the log_2_Foldchange value, and right column indicates the number of gene counts enriched in the pathway. I,J) Protein levels of TDO2, p‐NF‐κB, NF‐κB p‐iκBa, and iκBa in *TDO2*‐depleting Huh‐7 and *TDO2*‐overexpressing HepG2 cells with OA treatment (0.6 × 10^−3^ m) in the co‐culture system were examined by Western blotting.

To delve into how *TDO2* contributed to macrophage M1 polarization, we collected *TDO2*‐depleted Huh‐7 cells and its negative control counterparts in the upper chamber of co‐culture system, which were subsequently subjected to RNA‐Seq analysis. The reproducibility of the RNA‐Seq data across samples was conducted by Neighbor Joining (NJ) tree and Principal Component Analysis (PCA), as well as Spearman's correlation coefficient (Figure S11, Supporting Information), further affirming the validity of the data for further analysis. A total of 185 (0.96%) of 19 245 genes were identified as differentially expressed genes (DEGs) with a |log_2_(fold change)| ≥1 and a *p*‐value < 0.05, including 108 (0.56%) downregulated and 77 (0.4%) upregulated DEGs, respectively (**Figure** **7**A,B). Subsequently, functional enrichment analysis was conducted with BiNGO and revealed that the proliferation, differentiation, and activation of immune cells were over‐representative (Figure 7C). To gain further insights of biological processes and pathways, GSEA was performed to further identify the potential targets enriched by DEGs. Among that, lipid metabolic process, steroid metabolic process, and neutrophil extracellular trap formation were significantly enriched (Figure 7D‐F). Besides, KEGG database revealed NF‐κB pathway as the top canonical signaling pathway (*p* < 0.05) (Figure 7G,H), which is known as a classical pro‐inflammatory signaling pathway and has widely been reported to serve as a driving factor for macrophages M1 polarization.^[^ ^26^ ^]^ Further validation experiments demonstrated that the increased protein levels of p‐NF‐κB and p‐iκBa in Huh‐7 cells under OA treatment were significantly decreased due to *TDO2* depletion, while *TDO2‐*overexpressing HepG2 cells exhibited an opposite trend (Figure 7I,J). It has been reported that KYN/AHR axis mediated *TDO2*‐activated NF‐κB signaling pathway,^[^ ^27^ ^]^ we subsequently demonstrated that overexpression of *TDO2* enhanced the increased levels of *KYN* and *AHR* in HepG2 cells treated with OA (Figure S12A,B, Supporting Information), while the increased levels of *KYN* and *AHR* in Huh‐7 cells under OA treatment were significantly decreased due to *TDO2* depletion (Figure S12C,D, Supporting Information). Moreover, *KYN* promoted, while *AHR* inhibitor (CH‐223191) decreased the increased protein levels of p‐NF‐κB and p‐iκBa in hepatic cells under OA treatment (Figure S12E,F, Supporting Information). To strengthen the evidence supporting our proposed downstream pathway of TDO2, we demonstrated that protein levels of AHR, p‐NF‐κB and p‐iκBa were induced in HepG2 and Huh‐7 cells by OA in a dose‐dependent manner (Figure S13A,B, Supporting Information), as well as in the liver tissues of rats fed with HFD for different weeks during MASLD modeling process (Figure S13C, Supporting Information). Furthermore, the elevated protein levels of AHR, p‐NF‐κB and p‐iκBa in the liver tissues of rats fed with HFD were markedly decreased following sh*Tdo2* lentivirus administration (Figure S13D, Supporting Information). Therefore, these results manifested that *TDO2* participates in hepatic steatosis process via skewing macrophage toward M1 polarization by activating KYN/AHR/NF‐κB signaling pathway.

### 2.8 NPs‐Allo Potentiates Therapeutic Effects of Allopurinol on Hepatic Lipid Metabolic Disorders

**Figure 8:**
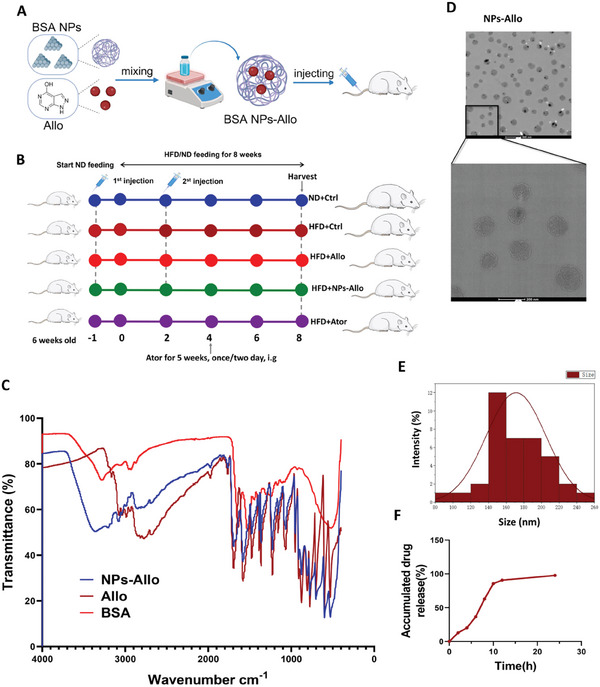
Characterizations of TDO2 inhibitor‐loaded BSA nanoparticles (NPs‐Allo). A) Schematic illustration of synthesis of NPs‐Allo. B) Schematic diagram of animal experiments design. In total 20 male rats of six weeks old were randomly divided into ND (*n* = 5) and HFD (*n* = 15) groups, which fed with standard‐diet or high‐fat diet for eight weeks, respectively. Rats were injected with free Allopurinol, NPs‐Allo or negative control via the tail vein as follows: normal diet with negative control (ND+Ctrl, *n* = 5), high fat diet with negative control (HFD+Ctrl, *n* = 5), high fat diet with positive control (HFD+Ator, *n* = 5), high fat diet with Allopurinol (HFD+Allo, *n* = 5), high fat diet with TDO2 inhibitor‐loaded BSA nanoparticles (HFD+ NPs‐Allo, *n* = 5). The rats were humanely sacrificed at the end of eight weeks for further effects evaluation. C) FTIR spectrum of NPs‐Allo, Allo, and BSA. D) TEM images of NPs‐Allo. Scale bars, 200 and 500 nm. E) The size distribution of NPs‐Allo. F) Release profiles of Allopurinol from NPs‐Allo.

Building on prior research, we delved deeper into the therapeutic possibilities for MASLD by focusing on TDO2 inhibition. To enhance therapeutic efficacy while minimizing side effects, we engineered a BSA‐based functional nanoparticle to administer Allopurinol (a TDO2 inhibitor) for MASLD treatment (**Figure** **8**A,B). FTIR spectroscopy results revealed that the allopurinol‐loaded BSA nanoparticle (NPs‐Allo) and free allopurinol exhibited nearly identical absorption peaks, confirming successful drug encapsulation. Absorption peaks in the ranges of 2900–3200 and 1500–1700 cm^−1^ correspond to the stretching vibration of ─OH (N─H) and C═N bonds within the aromatic structures of free allopurinol (Figure 8C). Transmission electron microscopy (TEM) was utilized to assess the morphology of allopurinol‐loaded BSA‐based nanoparticles (NPs‐Allo), with an approximate diameter of 140–180 nm (Figure 8D,E). The accumulated drug release reached about 100% about 20 h later (Figure 8F).

**Figure 9:**
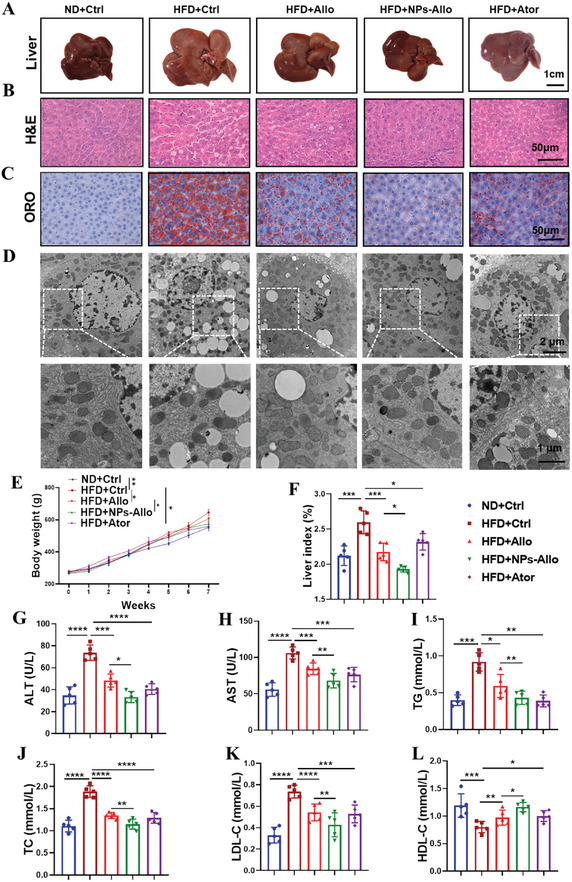
Effects of free Allopurinol and NPs‐Allo on liver lipid metabolism disorder in HFD‐fed rats. A) Representative liver morphology of ND+Ctrl, HFD+Ctrl, HFD+Ator, HFD+Allo, and HFD+NPs‐Allo rats (*n* = 3 per group). Scale bar, 1 cm. B) H&E staining showed that NPs‐Allo promoted the reduction of steatosis after Allopurinol treatment (*n* = 3 per group). Scale bar, 50 µm. C) Oil red O (ORO) staining showed that NPs‐Allo promoted the reduction of lipid deposition after Allopurinol treatment (*n* = 3 per group). Scale bar, 50 µm. D) The ultrastructure of rat hepatocytes in ND+Ctrl, HFD+Ctrl, HFD+Ator, HFD+Allo, and HFD+NPs‐Allo groups at 3600× (left, scale bar, 2 µm) and 8500× (right, scale bar, 1 µm) magnification (*n* = 3 per group). E) Body weight of rats in ND+Ctrl, HFD+Ctrl, HFD+Ator, HFD+Allo, and HFD+NPs‐Allo groups (*n* = 5 per group). F) Liver index of rats ND+Ctrl, HFD+Ctrl, HFD+Ator, HFD+Allo, and HFD+NPs‐Allo groups (*n* = 5 per group)). G–L) The content of serum ALT, AST, TC, TG, LDL‐C, and HDL‐C in the indicated groups (*n* = 5 per group). Results were shown as mean ± SD. *P*‐values are indicated by * < 0.05; ** < 0.01; *** < 0.001; **** < 0.0001 (*n* = 5 per group, two‐way ANOVA test in (E), others with one‐way ANOVA test). Blue: ND+Ctrl; Dark red: HFD+Ctrl; Red: HFD+Allo; Green: HFD+NPs‐Allo; Purple: HFD+Ator. ALT: Alanine aminotransferase; AST: Aspartate aminotransferase; TC: Total cholesterol; TG: Total triglycerides; LDL‐C: Low‐density lipoprotein cholesterol; HDL‐C: High‐density lipoprotein cholesterol.

To ascertain the therapeutic effects of NPs‐Allo on hepatic steatosis in HFD‐fed rats, Atorvastatin, which has been widely reported to play a protective role in MASLD,^[^ ^28^ ^]^ was used as a positive control. HFD/ND‐fed rats were injected with Allopurinol, NPs‐Allo or negative control via the tail vein (Figure 8B). **Figure** **9** exhibited a significant ameliorative effects of Atorvastatin on hepatic steatosis in rats induced by HFD. The liver of HFD+Ctrl rats turned yellow, with blunted edges and rough surface, of which improved after Allopurinol intervention and the improvement was more pronounced in the NPs‐Allo group. H&E staining showed that Allopurinol treatment apparently attenuated the hepatic lipid accumulation, and this trend was more significant in NPs‐Allo group (Figure 9B). Similarly, Oil red O staining and ultrastructure examination also showed the same trend in hepatic lipid deposition (Figure 9C,D). In addition, Allopurinol intervention effectively alleviated the increase in body weight and liver index induced by HFD, the inhibitory effect was more significant after the injection of NPs‐Allo (Figure 9E,F). Meanwhile, serum biochemical indicators showed that Allopurinol effectively alleviated the increase in serum AST, ALT, TG, TC, LDL‐C levels and the decrease in HDL‐C levels induced by HFD diet, and the effect was also amplified after treatment of NPs‐Allo (Figure 9G–L).

We logically explored the reasons why NPs‐Allo exhibited therapeutic superiority over direct injection of free Allopurinol molecules. The biodistribution of the NPs‐Allo, with indocyanine green (ICG) replacing Allopurinol, was initially monitored using the fluorescence‐imaging in vivo system (IVIS). 0.3 mg ICG or NPs‐Allo wrapped ICG (BSA(ICG)‐Allo) was venously injected into rats. In vivo and ex vivo imaging signals of fluorescence probes have shown that the nanoparticles exhibited substantial infiltration into the liver site within 30 min after administration and persisted up to 24 h with a relatively high retention (Figure S14A,B, Supporting Information). We subsequently determined the biocompatibility of NPs‐Allo and free Allopurinol in ND‐fed rats. As expected, NPs‐Allo was well tolerated in all rats as no significant body weight changes (Figure S15A, Supporting Information), normal tissue damage, and organ function abnormalities (e.g., liver function, renal function, and myocardial enzymes) were noted after treatment (Figure S15B, Supporting Information). In contrast, injection of free Allopurinol caused significant weight loss (Figure S15A, Supporting Information), abnormal variations of biochemical indicators, including liver function, renal function, and myocardial enzymes (Figure S15B, Supporting Information), indicating the adverse effects of the free Allopurinol treatment. Taken together, these results suggest NPs‐Allo potentiates therapeutic effects of Allopurinol on hepatic lipid metabolic disorder, likely due to its improved biodistribution and biocompatibility.

## 3 Discussion

MASLD is the most prevalent liver disease and represents a growing health concern globally due to its association with metabolic syndromes and cardiovascular diseases.^[^ ^29^ ^]^ In the present study, we investigated the genome‐wide patterns of H3K4me3, H3K27ac and H3K4me1 in the liver tissues affected by MASLD compared to normal liver tissues, and found that H3K27ac density in liver tissues was remarkably distinguished between normal and MASLD groups. Further integrating ChIP‐Seq and RNA‐Seq data, we identified upregulated genes associated with H3K27ac peaks in MASLD, which were significantly enriched in lipid metabolism process. Subsequently, Key PP (upregulated peak‐genes positively regulated by H3K27ac) gene *TDO2* was pinpointed, which was transcriptionally activated by *YY1*‐enhanced H3K27ac modification. This upregulation of *TDO2* promoted lipid accumulation via inducing polarization of macrophages towards a proinflammatory M1 phenotype through KYN/AHR/NF‐κB signaling pathway.

The role of H3K27ac in disease pathogenesis is not limited to MAFLD but extends to various other conditions.^[^ ^10^, ^30^ ^]^ For instance, in Alzheimer's disease, H3K27ac levels correlate with the regulation of genes involved in neuroinflammation and synaptic dysfunction.^[^ ^31^ ^]^ In cancers, H3K27ac marks oncogenes, tumor suppressors and influences epithelial–mesenchymal transition and metastasis.^[^ ^32^ ^]^ Similarly, in myocardial infarction, H3K27ac enhances expression of genes governing cell proliferation, contributing to cardiac repair.^[^ ^33^ ^]^ Autoimmune diseases like rheumatoid arthritis and systemic lupus erythematosus also involve H3K27ac‐mediated regulation of immune‐related genes.^[^ ^34^ ^]^ Thus, it is imperative to recognize that while H3K27ac is integral to the regulation of gene expression across a wide range of diseases, the precision of its action and the profound cellular responses it elicits are heavily context‐dependent on the specific disease setting. The influence of H3K27ac on disease mechanisms is largely sculpted by its intricate and dynamic interactions with a multitude of transcription factors, the chromatin structure, and the broader epigenetic environment.^[^ ^35^ ^]^ This underscores the critical importance of H3K27ac not only in the nuanced choreography of biological regulation but also in the trajectory of disease progression.

Besides, other histone modifications have also been implicated in metabolic disorders. For instance, histone H3K4 trimethylation (H3K4me3) marks a transgenerational epigenetic signal for lipid metabolism and increases their transcription response to multigenerational obesogenic effects.^[^ ^36^ ^]^ METTL3‐mediated modulation of H3K9ac and H3K27ac influences *Cd36* and *Ccl2* expression in nonalcoholic steatohepatitis (NASH) progression.^[^ ^37^ ^]^ JMJD3 regulates autophagy‐network genes through H3K27me3, impacting lipid degradation.^[^ ^38^ ^]^ Thereby, based on the pivotal roles played by histone modifications in metabolic‐related diseases, it is of great importance for us to further study the crosstalk of various histone modifications for a deep understanding of the pathogenesis of MASLD.

Yin Yang 1 (*YY1*) is a versatile zinc‐finger transcription factor known for its role a transcriptional repressor, activator, or initiator element binding protein.^[^ ^39^ ^]^ It has been implicated in various diseases due to its pivotal role in regulating cell proliferation and differentiation. Upregulation of *YY1* has been associated with proliferation, metastasis, treatment tolerance, and immunosuppression in various types of tumors.^[^ ^40^ ^]^ *YY1* has garnered attention in metabolic diseases, particularly MASLD in recent years.^[^ ^41^ ^]^ It has been reported that YY1/FAS signaling pathway played a critical role in therapeutic effects of Betulinic acid on MASLD.^[^ ^42^ ^]^ Moreover, *YY1* promoted hepatic steatosis through repression of farnesoid X receptor in obese mice.^[^ ^43^ ^]^ Targeting mTOR/YY1 signaling pathway by quercetin, through CYP7A1‐mediated cholesterol‐to‐bile acids conversion, alleviated hepatic lipid accumulation induced by type 2 diabetes mellitus.^[^ ^44^ ^]^ All of these previous studies convincingly demonstrated that *YY1* served as a crucial driving factor in the progression of MASLD. In our study, we discovered *YY1* promoted transcription of *TDO2* by activating H3K27ac modification in its gene region, thereby exacerbating the progression of hepatic steatosis. Our findings unveil a novel mechanism where the TDO2/NF‐κB signaling pathway induces M1 polarization in macrophages during YY1‐mediated MASLD.

*TDO2* has gained considerable attention in multiple tumor types,^[^ ^14^, ^45^ ^]^ including liver cancer.^[^ ^17^ ^]^ Nevertheless, its specific functional mechanisms in metabolic diseases have been less studied. Previous research has suggested that *TDO2* may act as an important mediator of cross‐communication between hepatocytes and macrophages in regulating liver inflammation,^[^ ^19^ ^]^ *TDO2* expression was increased in the liver of HFD‐fed mice.^[^ ^46^ ^]^ In our study, we systematically validated the expression pattern, explicit functions, and underlying molecular mechanisms of *TDO2* in hepatic steatosis. A TDO2 inhibitor‐loaded BSA nanoparticle was designed to achieve excellent therapeutic effects on MASLD due to the superiority of biodistribution and biocompatibility. It has been reported *TDO2* controls M2 macrophages polarization to promote esophageal squamous cell carcinoma progression via AKT/GSK3b/IL‐8 signaling pathway,^[^ ^14a^ ^]^ suggesting a correlation between *TDO2* and suppression of inflammatory responses. However, high level of *TDO2* was demonstrated to be associated with pro‐inflammatory cytokines in synovium and synovial fluid of patients with osteoarthritis.^[^ ^47^ ^]^ Additionally, *TDO2* inhibition has been proven to ameliorate autoimmune arthritis in rats through decreasing M1/M2 ratio,^[^ ^15^ ^]^ suggesting the pro‐inflammatory role of *TDO2*. Thus, the specific role of *TDO2* in inflammatory responses depends on the concrete context of diseases and is not universally consistent. The present study focuses on impacts of *TDO2* inhibition on lipid levels and macrophage polarization. A hepatic‐*Tdo2*‐specific KO mouse is being constructed and the long‐term effects of *TDO2* inhibition on liver function and overall metabolism (such as hepatic fibrosis, cirrhosis and even hepatocellular carcinoma) would be systematically studied in the near future.

**Figure 10:**
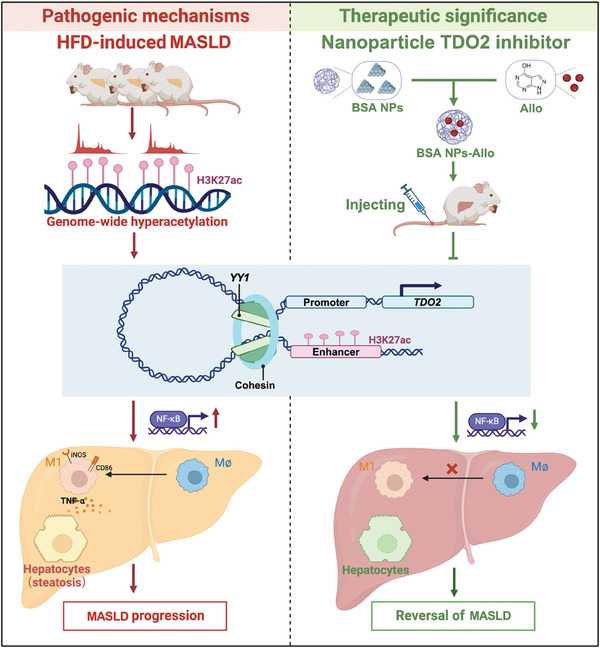
A hypothetical mechanism for epigenetic regulation of H3K27ac leading to the development of MASLD in rats and potential therapeutic mechanisms. A proposed model suggests that HFD induced a significantly increase in histone H3K27 acetylation, which resulting in the remodeling of chromatin structure. Then, the enhancer was recruited by transcription factors *YY1*, promoting the expression of gene *TDO2*, which promoted macrophages M1 polarization by activating NF‐κB pathway to facilitate occurrence and development of MASLD. Based on this, we proposed a potential treatment approach for MASLD: a TDO2 inhibitor (Allopurinol)‐loaded BSA nanoparticle was designed to inhibit *TDO2* activity, further inhibit M1 polarization of macrophages and mitigate MASLD progression.

Our study focused on the profile of H3K27ac in HFD‐fed rats model, and proved H3K27ac is a representative histone marker in regulation of key genes in MASLD. Hepatocyte *TDO2* was identified as a critical positive regulator of abnormal lipid accumulation of liver, which was transcriptionally activated by *YY1*‐promoted H3K27ac modification. *TDO2* shifted macrophage towards M1 polarization via activating NF‐κB signaling pathway to facilitate hepatic steatosis. And *TDO2* inhibitor‐loaded BSA nanoparticles (NPs‐Allo) showed remarkable therapeutic effects in HFD‐fed rats, representing a promising strategy for MASLD. Collectively, our findings demonstrate novel functions of H3K27ac and *TDO2* in MASLD and provide promising therapeutic targets for both prevention and treatment of this disease (**Figure** **10**).

## 4 Experimental Section

### Animal Models and Treatments

Sprague Dawley littermate rats purchased from the Vital River Laboratory Animal Technology Co. Ltd. (Beijing, China) were allowed to adapt for one week before experiment. The rats were housed in a specific pathogen‐free environment (24–26 °C, relative humidity 50%−60%) with a 12 h light/dark cycle and free access to food and water. To establish a MASLD model, 20 male 6‐week‐old rats were randomly divided into ND and HFD groups, which fed with normal diet (20.6% protein, 12% fat, and 67.4% carbohydrate) and high fat diet (20.2% protein, 45.4% fat, and 34.5% carbohydrate) for 8 weeks, respectively. The body weight of each rat was recorded weekly. Collected liver tissue and plasma samples from rats fed with HFD for 0, 4, 6, and 8 weeks to verify the successful construction of the HFD induced MASLD model after the rats were sacrificed humanely. To determine the function of *TDO2* in vivo, rats were injected with lentivirus‐containing shRNAs against *Tdo2* or shNC via the tail vein: normal diet with shNC (ND + shNC, *n* = 5), high fat diet with shNC (HFD + shNC, *n* = 5), and high fat diet with shTdo2 (HFD + sh*Tdo2*, *n* = 5). To evaluate the therapy effects of TDO2 inhibitor (Allopurinol) and TDO2 inhibitor‐loaded BSA nanoparticles (NPs‐Allo), Atorvastatin was used as a positive control (100 mg kg^−1^, once/two day, intragastric administration, *n* = 5). Rats were injected with free Allopurinol, NPs‐Allo, or negative control via the tail vein as follows: normal diet with negative control (ND + Ctrl, *n* = 5), high fat diet with negative control (HFD + Ctrl, *n* = 5), high fat diet with Allopurinol (HFD + Allo, 1.5 mg kg^−1^, *n* = 5), and high fat diet with TDO2 inhibitor‐loaded BSA nanoparticles (HFD + NPs‐Allo, 1.5 mg kg^−1^, *n* = 5). Similarly, at the end of each experiment, liver tissues and plasma samples were collected for further examination. The IVIS was used to evaluate the biodistribution of the BSA nanoparticle, as previously described.^[^ ^48^ ^]^ A fluorescent probe ICG was encapsulated into BSA nanoparticle to indicate the in vivo distribution at different time points following venous injection into the rats. All animal experiments in this study were conducted according to the national legislation and the guidelines of the laboratory animal center at Anhui Medical University (Hefei, China) (LLSC20230636). All animal experimental procedures were approved by the Ethics Committee of Anhui Medical University.

### Histopathological Examination

All histological analyses were performed by Servicebio Technology Co. Ltd. (Wuhan, China). Briefly, fresh liver tissues were dissected and fixed in 10% neutral buffered formation, then paraffin sections were stained with H&E Staining Kit (Beyotime, Beijing, China) and an ORO Staining Kit (Solarbio, Beijing, China). The histological samples were observed and imaged using a light microscope (ECLIPSE 80i, Nikon, Tokyo, Japan).

### Serum Analysis

Serum levels of total cholesterol (TC), triglyceride (TG), aspartate aminotransferase (AST), alanine aminotransferase (ALT), albumin (ALB), blood urea nitrogen (BUN), creatinine (Crea), creatine kinase (CK), creatine kinase isoenzyme (CK‐MB), high‐density lipoprotein cholesterol (HDL‐C), and low‐density lipoprotein cholesterol (LDL‐C) were measured with a HITACHI Automatic Analyzer (3100, Tokyo, Japan) according to the manufacturer's protocol. The pro‐inflammatory factor interleukin‐1α (IL‐1α), interleukin‐1β (IL‐1β), and tumor necrosis factor‐α (TNF‐α) levels were measured with the rat ELISA Kit (Shibayagi, Gunma, Japan).

### Ultrastructure Examination

Liver samples measured 1 mm^3^ were fixed in 3% glutaraldehyde at 4 °C, postfixed in 1% OsO4, dehydrated in increasing concentrations of ethyl alcohol (50%, 70%, 90%, 100%) and embedded in resin. The ultrathin sections were cut on a Leica EM UC7 ultramicrotome, stained with uranyl acetate and lead citrate, and assessed under a Thermoscientific Talos L120C G2 transmission electron microscope.

### ChIP‐Seq and Differential Analysis of H3K27ac Peaks

deepTools (https://deeptools.readthedocs.io/en/develop/) was used to profile the ChIP‐seq data of histone modification of H3K27ac, H3K4me3, and H3K4me1 between normal and MASLD samples. SimpleChIP enzymatic chromatin IP kit (9005, Cell Signal Technology) was used to perform chromatin immunoprecipitation (ChIP) analysis with three samples in each group, and the detailed operation process according to standard protocols. Then ChIP and input libraries were prepared according to the manufacturer's protocol, and sequencing procedures were performed by HiSeq 2500 platform (Illumina) by Novogene (United States). Subsequently, differential analysis of H3K27ac peaks was performed as previously described.^[^ ^49^ ^]^ Briefly, clean reads were mapped to rat reference genome Rattus_norvegicus_6.0. Model‐based Analysis for ChIP‐Seq version 2.1.0 (MACS 2.1.0) was conducted to obtain H3K27ac peak files with a *q*‐value threshold of 1 × 10^−5^.^[^ ^50^ ^]^ Next, to quantify MASLD‐related differentially acetylated peaks between normal and MASLD rats, all BAM files were sorted and analyzed with the samtools (version 1.2) “bedcov” utility. Meanwhile, empirical coverage was estimated by comparing the coverage within 20 bp bins across one megabase of mappable rat reference genome sequence. ChIP and input sample coverage were normalized by total mapped read count and peak length, with the subtraction of input coverage from ChIP coverage to limit the influence of fragmentation bias. Finally, DESeq2 R package was adopted to perform differential H3K27ac analysis with a *p*‐value < 0.05 and |log_2_foldchange| ≥1. The analyses were conducted in R version 3.5.1.

### RNA‐Seq and Differential Gene Expression Analysis

RNA was isolated from the same liver tissues of corresponding samples from ChIP‐Seq with Trizol (Invitrogen, USA) according to the manufacturer's protocol. Then cDNA libraries were constructed with the Extracted RNA through NERNext Ultra Directional RNA Library Prep Kit for Illumina R (NEB, USA). The paired‐end sequencing of the libraries was constructed on a Hi‐Seq 4000 platform (Illumina, USA) via Novogene (Novogene, Beijing, China). Next, filtered reads were mapped to the rat reference genome of Rattus_norvegicus_6.0 by STAR‐2.5.3a^[^ ^51^ ^]^ and mapped reads were counted by Feature Count software,^[^ ^52^ ^]^ the fragments per kilobase of transcript sequence per millions base pairs (FPKM) algorithm was used to normalize the expression of each gene. Afterward, DEGs were adopted by DESeq2 with the cutoff criteria of |log_2_ (fold change) |≥1 and *p*‐value < 0.05. And the detailed information please refer to a previous study described.^[^ ^53^ ^]^

### Integrative Analysis of H3K27ac ChIP‐Seq and RNA‐Seq

An integrative analysis of H3K27ac ChIP‐Seq in acetylome and RNA‐Seq in transcriptome was conducted by calculating Pearson correlation coefficient (PCC) between H3K27ac peaks and genes whose TSSs were positioned within a 1000‐kb window of the ChIP‐Seq peaks. This approach was used to identify putative H3K27ac peak‐targeted genes, aligning with the methodology described in the publication by Hongbo et al.^[^ ^24^ ^]^ Pearson correlation coefficient ≥ 0.5 and *p*‐value < 0.05 were set as the criteria for correlated peak‐gene and conducted Genome‐wide “four‐way” according to the regulated direction of differential peak‐genes by H3K27ac at a threshold of log_2_foldchange (|ChIP| > 1 and |RNA| > 5) to screen out the PP peak‐genes (upregulated peak‐genes positively regulated by H3K27ac (log_2_foldchange (ChIP > 1 and RNA > 5)) and NN peak‐genes (downregulated peak‐genes negatively regulated by H3K27ac (log_2_foldchange (ChIP < −1 and RNA < −5)) for further investigation. Finally, DAVID (http://david‐d.ncifcrf.gov/), PANTHER (www.pantherdb.org/), and KEGG (http://www.genome.jp/kegg/) were executed to understand the functional enrichment of these differentially regulated peak‐genes.

### Determination of Core Transcription Factor

To screen out the core transcription factor of *TDO2*, transcription factor prediction was performed as previously described.^[^ ^54^ ^]^ The summary steps are as follows: the TFs binding with the enhancer and promoter of *TDO2* was first predicted based on UCSC (http://genome‐asia.ucsc.edu) and PROMO (http://alggen.lsi.upc.es/) database. Then, protein–protein interaction (PPI) network of top 20 overlapped TFs related to both promoter and enhancer of *TDO2* was constructed to screen the crucial TFs at the core position. Next, homology analysis was used to investigate the homologies of *TDO2* and YY1 among human, mouse, and rat, and the TFs of *TDO2* in human and mouse were also predicted. Finally, further validation of candidate transcription factors was conducted in the Cistrome (http://cistrome.org/) database.

### Cell Culture and Treatment

The human hepatocyte cell lines HepG2 and Huh‐7, human monocyte cell line THP‐1 were purchased from the American Type Culture Collection (ATCC, Rockville, MD). All these three cell lines were cultured in DMEM (Invitrogen) with 10% FBS (Gibco) and 1% penicillin/streptomycin (Sigma) and maintained in a 5% CO_2_ incubator at 37 °C. HepG2 and Huh‐7 cells were used to establish in vitro models of hepatic steatosis as recommended.^[^ ^55^ ^]^ THP‐1 cells were differentiated from macrophages as described previously.^[^ ^56^ ^]^ Co‐culture system: 10^5^ OA‐induced HepG2/ Huh‐7 cells with *TDO2* overexpression/ knockdown were respectively seeded into the upper chamber of a transwell (0.4 µm pore size; Corning), 10^6^ THP‐1‐derived macrophages were seeded into the bottom chamber. Cells were co‐cultured in a standard humidified incubator at 37 °C in a 5% CO_2_ atmosphere for 72 h and subjected to subsequent assays, respectively.

### RNA Exaction and Quantitative Real‐Time PCR Analysis

The isolated RNA from cells was converted into cDNA by using RevertAid First Strand cDNA Synthesis Kit (Thermo Scientific Bio) following the manufacturer's manual, the resulting cDNA samples were analyzed by quantitative real‐time PCR using a PerfectStart Green qPCR SuperMix (TransGen) on a CFX96 Touch Real‐Time PCR Detection System (Bio‐Rad, CA). All primers used are listed in Table S1 (Supporting Information).

### Drugs, siRNAs and Plasmids

Curcumin was obtained from Shanghai Yuanye Bio‐Technology Co., Ltd. (Shanghai, China). Atorvastatin (used in the in vivo experiments) was purchased from Pfizer, Inc. Oleic acid (OA), KYN, AHR antagonist (CH‐223191), and Allopurinol (Allo) were purchased from Sigma‐Aldrich (St. Louis, MO). siRNAs against *YY1* and *TDO2* were synthesized by Shanghai GenePharma Co., Ltd. Full‐length *YY1* and *TDO2* CDS were amplified and inserted into pCDH vector according to the manufacturer's instructions. siRNAs and plasmids were transfected into corresponding cells by using Lipofectamine 2000 (Invitrogen, MA, USA) according to the instruction. Oligonucleotides used are listed in Table S2 (Supporting Information).

### Western Blotting Analysis

Western blot was performed as previously described.^[^ ^57^ ^]^ All primary antibodies used are listed in Table S3 (Supporting Information).

### Lentivirus Production

shRNAs targeting rat *Tdo2* were synthesized and inserted into pLKO.1 vector. Lentivirus production and transduction were performed as previously described.^[^ ^58^ ^]^ All primers used are listed in Table S2 (Supporting Information).

### Luciferase Activity Assay

*TDO2* promoter (2 kb) was amplified and inserted into pGL3‐Basic luciferase reporters as recommended. The mutant *TDO2* promoter pGL3‐Basic vector was amplified by using special primers according to the wide‐type *TDO2* promoter pGL3‐Basic vector. pRL‐TK plasmid was provided as an internal transfection control. The detailed protocol of luciferase reporter assays was described in the previous study.^[^ ^59^ ^]^ All primers used are listed in Table S2 (Supporting Information).

### Bodipy 493/503 Fluorescence Staining

HepG2 and Huh‐7 cells with indicated treatments were stained with Bodipy 493/503 as previously described.^[^ ^55a^ ^]^ Images of stained cells were captured by ZEISS AXIO fluorescence microscope.

### Determination of Intracellular TG Content

The intracellular contents of TG in HepG2 and Huh‐7 cells with indicated treatments were determined using a triglyceride assay kit (GPO‐POD; Applygen Technologies Inc., Beijing, China) according to the manufacturer's protocol. BCA protein assay kit (Beyotime, Jiangsu, China) was used to measure the protein concentration, the intracellular content of TG was normalized to the total protein concentration in the cell lysates.

### Immunofluorescence Assay

Immunofluorescence assay was performed to examine the protein expressions in cells and liver tissue sections with different treatments by using an Immunol Fluorescent Staining Kit (Beyotime, Jiangsu, China) according to the manufacturer's instructions. Images were captured by ZEISS AXIO fluorescence microscope. All primary antibodies used are listed in Table S3 (Supporting Information).

### Flow Cytometry

Percentage of CD86^+^ (374208, Biolegend) THP‐1‐dirived macrophages in the bottom chamber of co‐culture system were detected by flow cytometry as previously described.^[^ ^60^ ^]^ The samples were harvested and analyzed with a Cyto FLEX flow cytometry (Beckman, Germany).

### Elisa Analysis

KYN concentration was determined by using a human KYN ELISA Kit purchased from Keshun (China) according to the guidance.

### Preparation and Characterization of Allopurinol‐BSA Nanoparticles (NPs‐Allo)

To form allopurinol‐BSA conjugate, 5 mg allopurinol is dissolved in 1 m NaOH and adjust the pH of the solution to ≈9.8 with hydrochloric acid. Add 100 mg BSA to the solution and stir until a suspension was created, which indicates the formation of allopurinol‐BSA NPs. Crosslink the allopurinol‐BSA NPs with a 2.5% glutaraldehyde solution at room temperature. Wash the crosslinked allopurinol‐BSA nanoconjugates with distilled water and centrifuge at 15,000 rpm. The particles that were collected underwent reconstitution in phosphate‐buffered saline (PBS) with a pH of 7.4 after undergoing sonication. The size and morphology of NPs‐Allo were characterized by TEM. The surface modification of NPs‐Allo, Allo, and BSA was measured using an FTIR spectrophotometer. For assessment of drug release, 1 mL NPs‐Allo was dissolved in 4 mL ddH2O (pH 7.4) to form a release system after determining the packaging dose, then rotated continuously at 37 °C (200 rpm). 1 mL sample was removed at the indicated intervals and the release system was resupply with the same volume of dd H2O. The amount of drug released at each time point was measured using enzyme‐labeled instrument (OD = 300 nm).

### Statistical Analysis

GraphPad Prism 8.0 software was used for data processing and analysis. Data are presented as mean ± standard deviation (SD) from at least three independent experiments. Student *t*‐test, one‐way ANOVA or two‐way ANOVA was used for group comparisons appropriately. Spearman Pearson correlation analysis was used to assess the association between *TDO2* mRNA level and H3K27ac density. *P*‐values were categorized as follows: * *p* < 0.05; ** *p* < 0.01; *** *p* < 0.001.

## Conflict of Interest

The authors declare no conflict of interest.

## Author Contributions

Y.Z., L.S., and Y.T. contributed equally to this work. Y.Z., B.C.Z., and H.Q.Z. conceived and designed the experiments. A.Y.H. helped to conceive and design the experiments. Y.Z., Y.L.Z., L.M.S., and Y.S.T. together performed experiments, analyzed data, wrote and revised the manuscript. T.T.Z., B.X., S.W.C., X.R.Y., Q.S.L., and L.D. helped to perform animal experiments and bioinformatic analysis. Y.W., J.H.M., X.Y.L., and J.P. analyzed data. All authors read and approved the final version of the manuscript.

## Supporting information



Supporting Information

## Data Availability

The data that support the findings of this study are available from the corresponding author upon reasonable request.
