# Supplementary material for: Genome‐Wide Profiling of H3K27ac Identifies TDO2 as a Pivotal Therapeutic Target in Metabolic Associated Steatohepatitis Liver Disease
Source: Adv Sci (Weinh). 2024 Oct 4;11(45):2404224. doi: 10.1002/advs.202404224 (PMC11615751; doi:10.1002/advs.202404224)
Supplement: Supplementary file 1 — Supporting Information [file ADVS-11-2404224-s001.docx]

**Genome-wide Profiling of H3K27ac Identifies TDO2 as a Pivotal Therapeutic target in Metabolic Associated Steatohepatitis Liver Disease**

Yaling Zhu^#^, Limeng Shang^#^, Yunshu Tang^#^, Qiushuang Li, Lin Ding, Yi Wang, Tiantian Zhang, Bin Xie, Jinhu Ma, Xinyu Li, Shuwen Chen, Xinrui Yi, Jin Peng, Youfeng Liang, Anyuan He, Hong Yan, Huaqing Zhu*, BuChun Zhang*, Yong Zhu*

Supplemental Figure


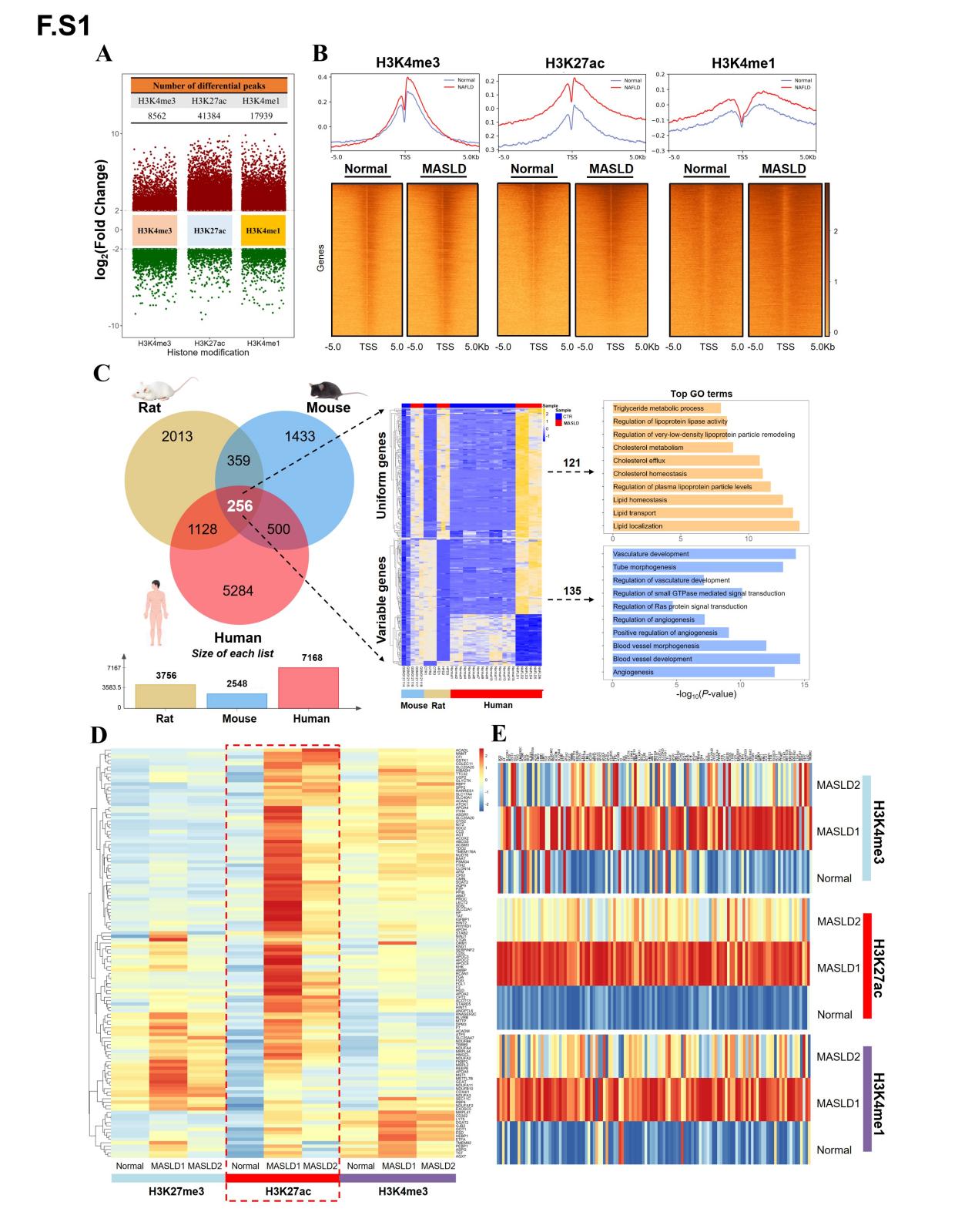


**Figure S1. H3K27ac is a representative histone marker in the regulation of key genes in MASLD** A) The number of differential peaks of H3K4me3, H3K27ac and H3K4me1 between normal and MASLD-related patients. The red and green indicated upregulation and downregulation, respectively. B) Heatmap showing the density of enhancers marked by H3K4me3, H327ac, and H3K4me1 ±5 kb near the transcription start site (TSS) in normal and cirrhosis patients (n= 3 per group). C) Venn diagram illustrates the overlap of differentially expressed genes between MASLD and control from mouse (Control=2, MASLD=3), rat (n=3 per group) and human (Normal=15, MASLD=6), showing the overlapping genes numbers between mouse, rat and human. Heatmap of 256 overlapped genes in mouse, rat and human. And Pathway enrichment analysis of 121 uniform genes and 135 variable genes with Metascape, respectively. D, E) The peak heatmaps of H3K27ac, H3K4me1, and H3K4me3 of these uniform genes.


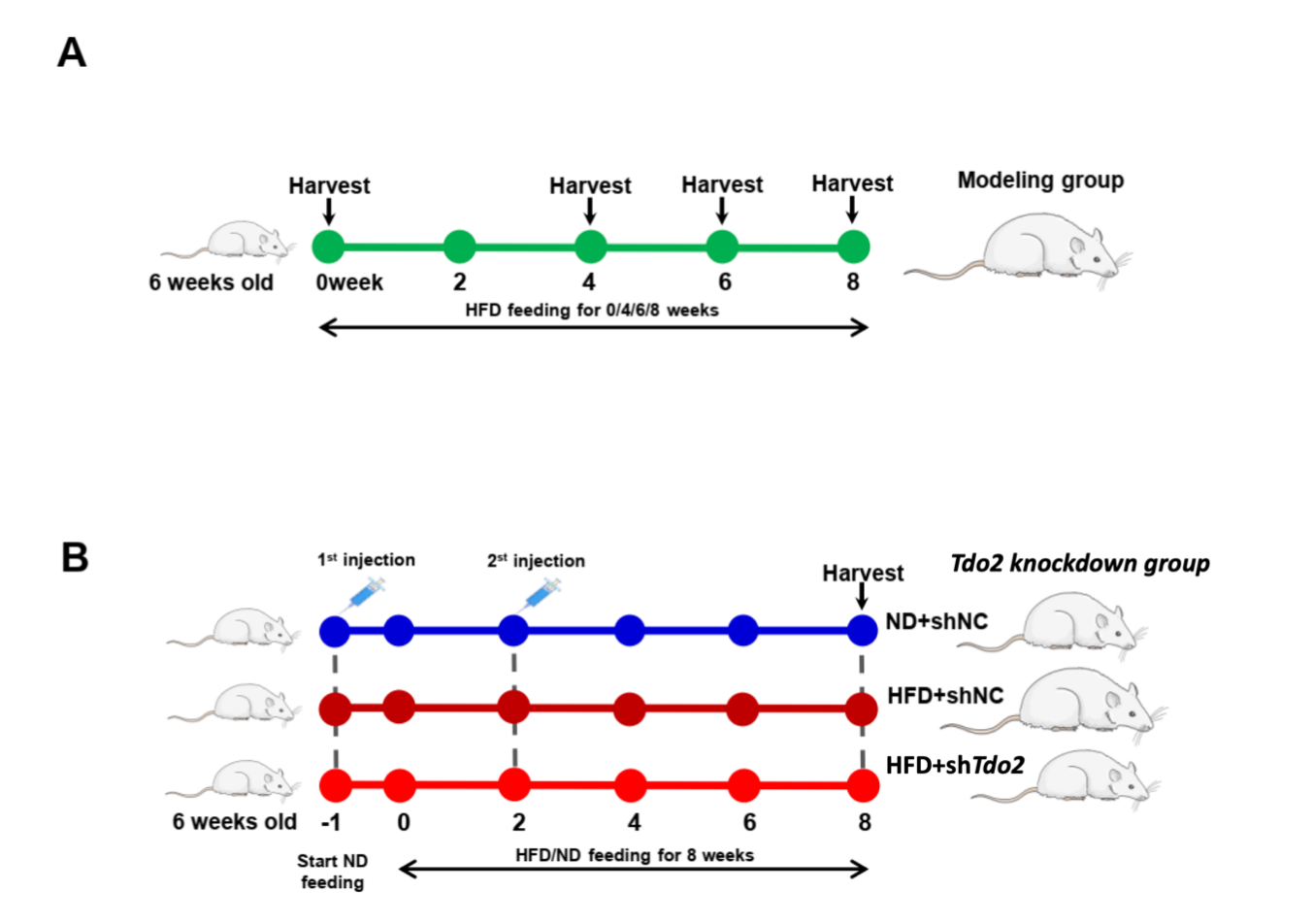


**Figure S2. Schematic diagram of animal experiments design**. Sprague Dawley rats purchased from the Vital River Laboratory Animal Technology Co. Ltd. A) In total 20 male rats of 6 weeks old were randomly divided into ND (n= 5) and HFD (n= 15) groups, which were fed with standard-diet (20.6% protein, 12% fat and 67.4% carbohydrate) and high-fat diet (20.2% protein, 45.4% fat and 34.5% carbohydrate), respectively. The body weight of each rat was recorded weekly, and liver and plasma samples were collected at 0, 4, 6 and 8 weeks of HFD feeding for verification of HFD-induced MASLD model. B) In total 15 male rats of 6 weeks old were randomly divided into ND (n= 5) and HFD (n= 10) groups, which were fed with standard-diet or high-fat diet for 8 weeks, and injected with lentivirus-containing shRNAs against *Tdo2* via the tail vein at the indicated weeks as follows: standard-diet with shNC (ND+shNC), high-fat diet with shNC (HFD+shNC), high-fat diet with shTDO2 (HFD+sh*Tdo2*,)((n= 5 per group). The rats were humanely sacrificed at the end of 8 weeks for further effects evaluation.


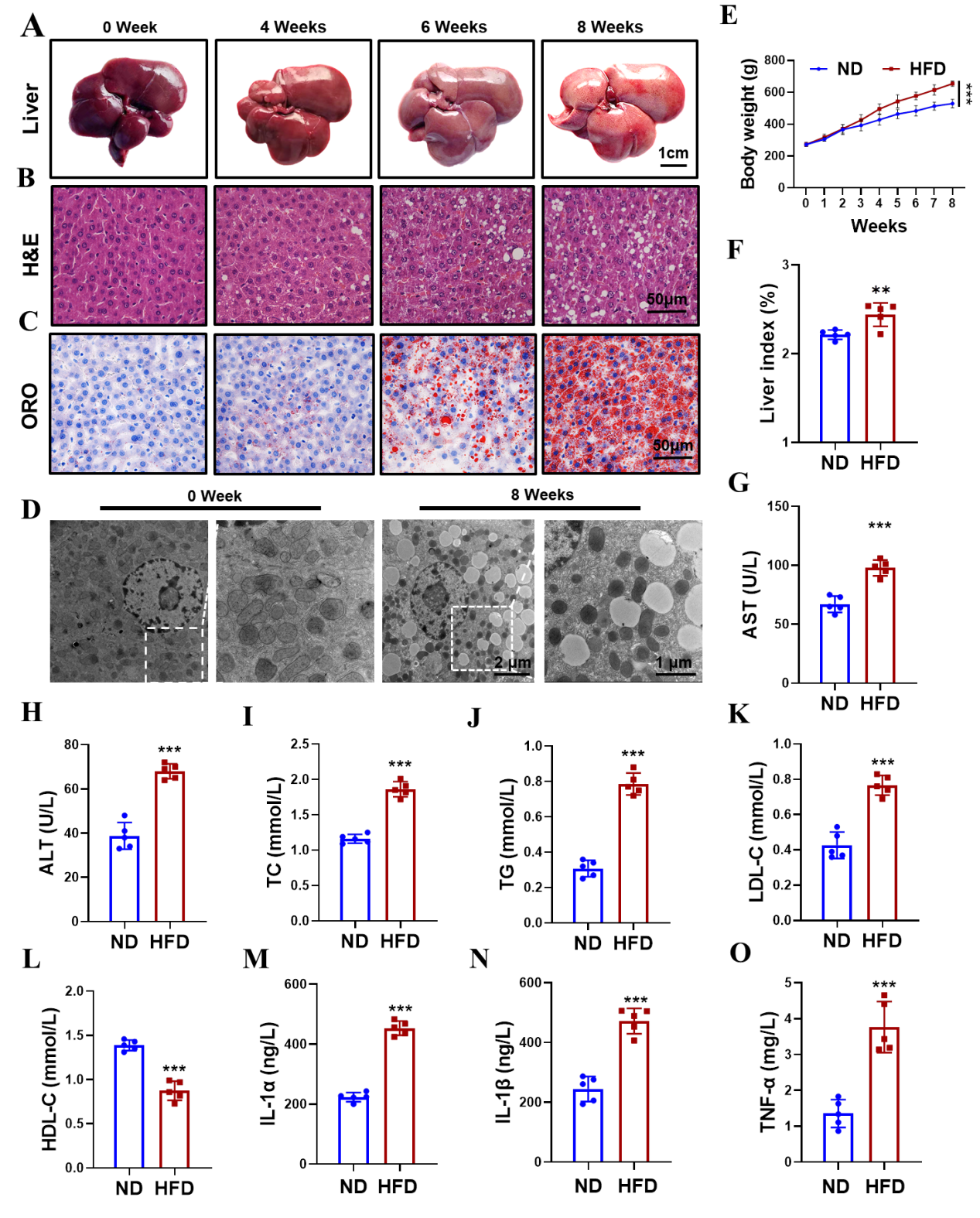


**Figure S3. Verification of HFD-induced hepatic steatosis model in rats.** A) Liver gross specimens of rats fed with HFD for 0, 4, 6 and 8 weeks, respectively. (scale bar, 1 cm) (n= 3 per group). B) Representative Hematoxylin-Eosin (H&E) staining of liver sections from HFD-fed rats in the indicated period, respectively (n= 3 per group). Scale bar, 50 μm. C) Oil Red O (ORO) staining indicated lipid accumulation in liver tissues at different time points. (n= 3 per group). Scale bar, 50 μm. D) Electron microscopy images of hepatocytes at 3600×(left, scale bar, 2 μm) and 8500× (right, scale bar, 1 μm) magnification showed ultrastructural changes after 0 and 8 weeks of HFD feeding (n=3 per group). E) The changes in body weight of rats in the two groups during the experiment stage. (n= 5 per group). F) Liver index of rats after 8 weeks of HFD feeding (n= 5 per group). G-L) The content of serum AST (G), ALT (H), TC (I), TG (J), LDL-C (K) and HDL-C (L) in the indicated groups (n= 5 per group). M-O) Levels of inflammatory factors IL-1α (M), IL-1β (N) and TNF-α (O) in serum between ND and HFD group (n= 5 per group). Results were shown as mean ± SD. *P* values are indicated by * < 0.05; ** < 0.01; *** < 0.001 (n=5 per group, two-way ANOVA test in E, others with unpaired two-sided Student’s t-test). ND, normal diet; HFD, high fat diet; ALT, alanine transaminase; AST, aspartate transaminase; TG, triglyceride; TC, total cholesterol; LDL-C, low density lipoprotein cholesterol; HDL-C, high density lipoprotein cholesterol; IL-1α: interleukin-1α, IL-1β: interleukin-1β, TNF-α: tumor necrosis factor-α.


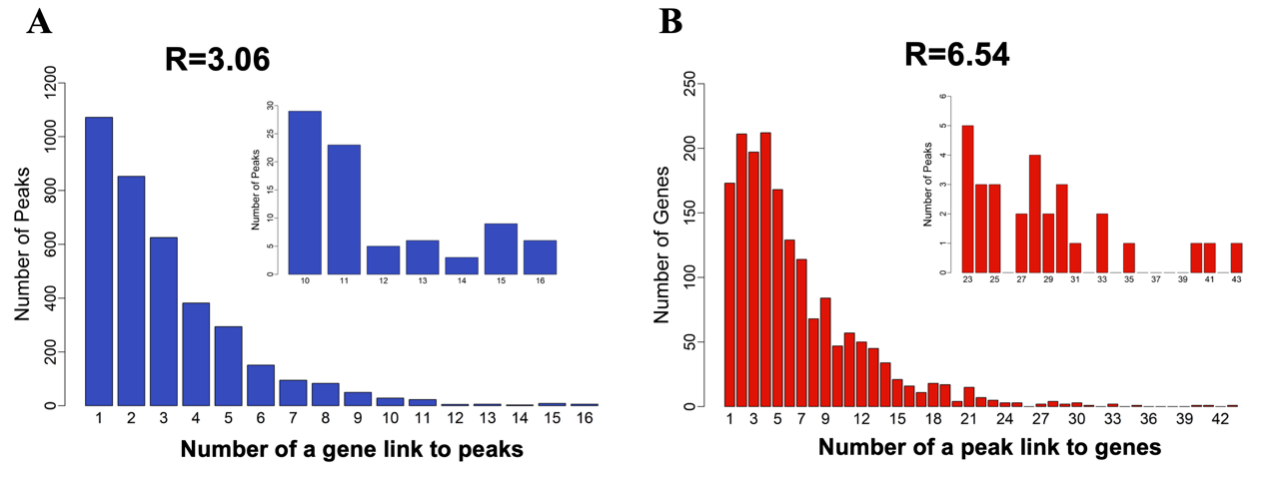


**Figure S4. The regulatory relationship between the H3K27ac peaks and genes.** A) The number of a gene link to peaks. There are 3.06 H3K27ac peaks connected to one gene on average. B) The number of a peak link to genes. There are 6.54 genes connected to one peak on average.

**
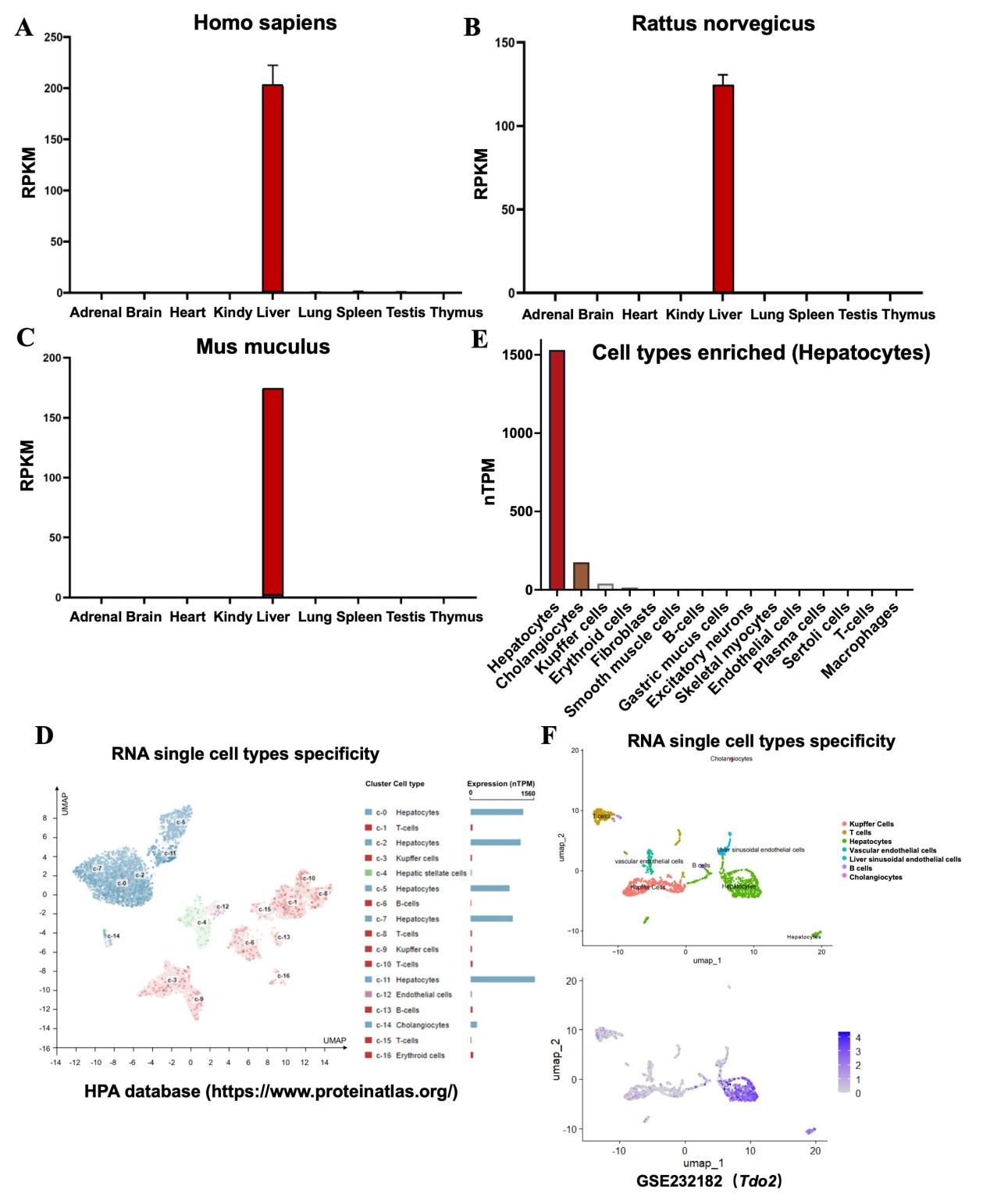
**

**Figure S5.** ***TDO2* is highly enriched in hepatocytes**. A-C) The tissue localization analysis of *TDO2* in different tissues of Homo sapiens (A), Rattus norvegicus (B) and Mus musculus (C) was performed using data from PRJEB443, PRJNA238328, and PRJNA66167 in the NCBI public database (http://www.ncbi.nlm.nih.gov). D) The cell localization analysis of *TDO2* in different cell types in liver tissue using the HPA public database (https://www.proteinatlas.org/). E) The top 15 cell types enriched by *TDO2* using the HPA public database (https://www.proteinatlas.org/). F) The cell localization analysis of *Tdo2* in different cell types in liver tissue using the single-cell data of GEO public database (GSE232182).


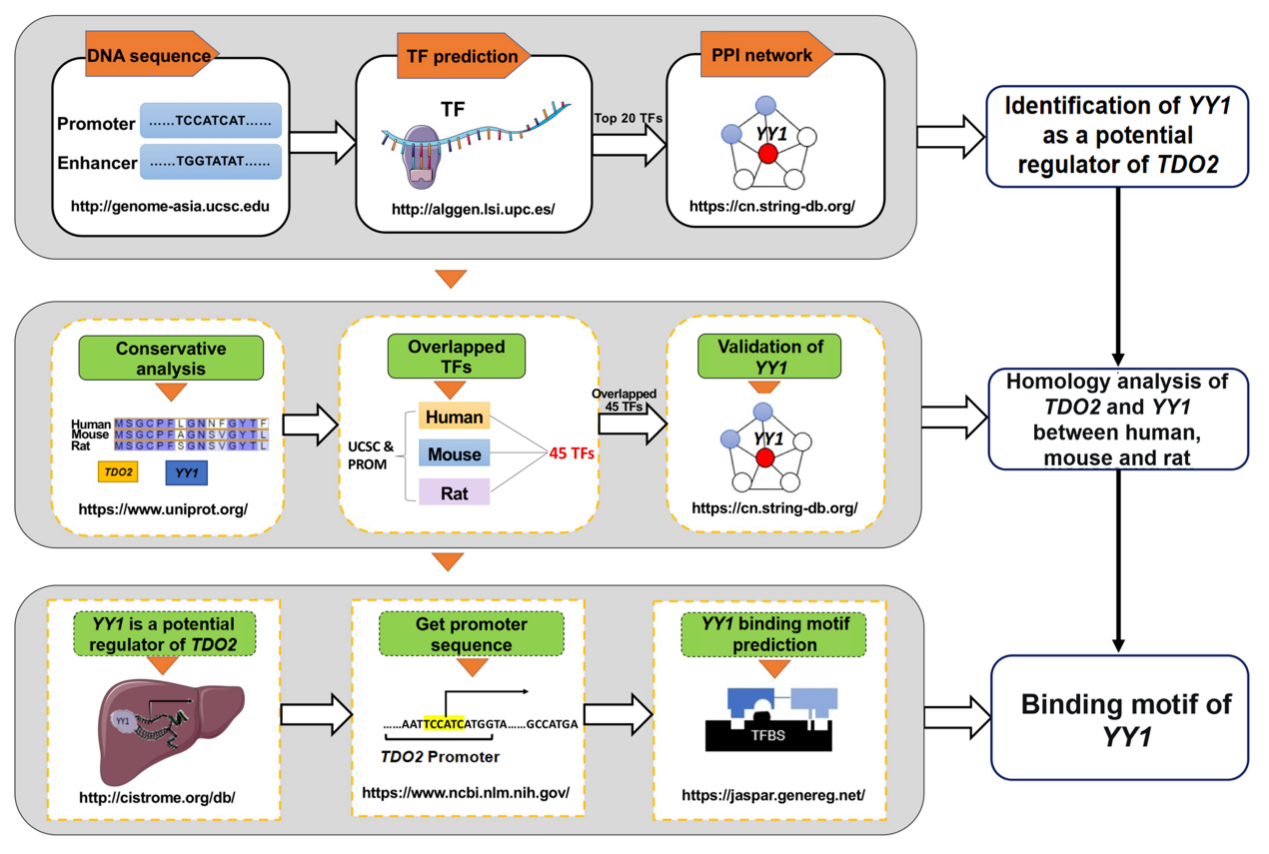


**Figure S6.** A schematic overview of the process for identifying the core transcription factor of *TDO2*, followed by further analysis of homology and binding motifs.

**
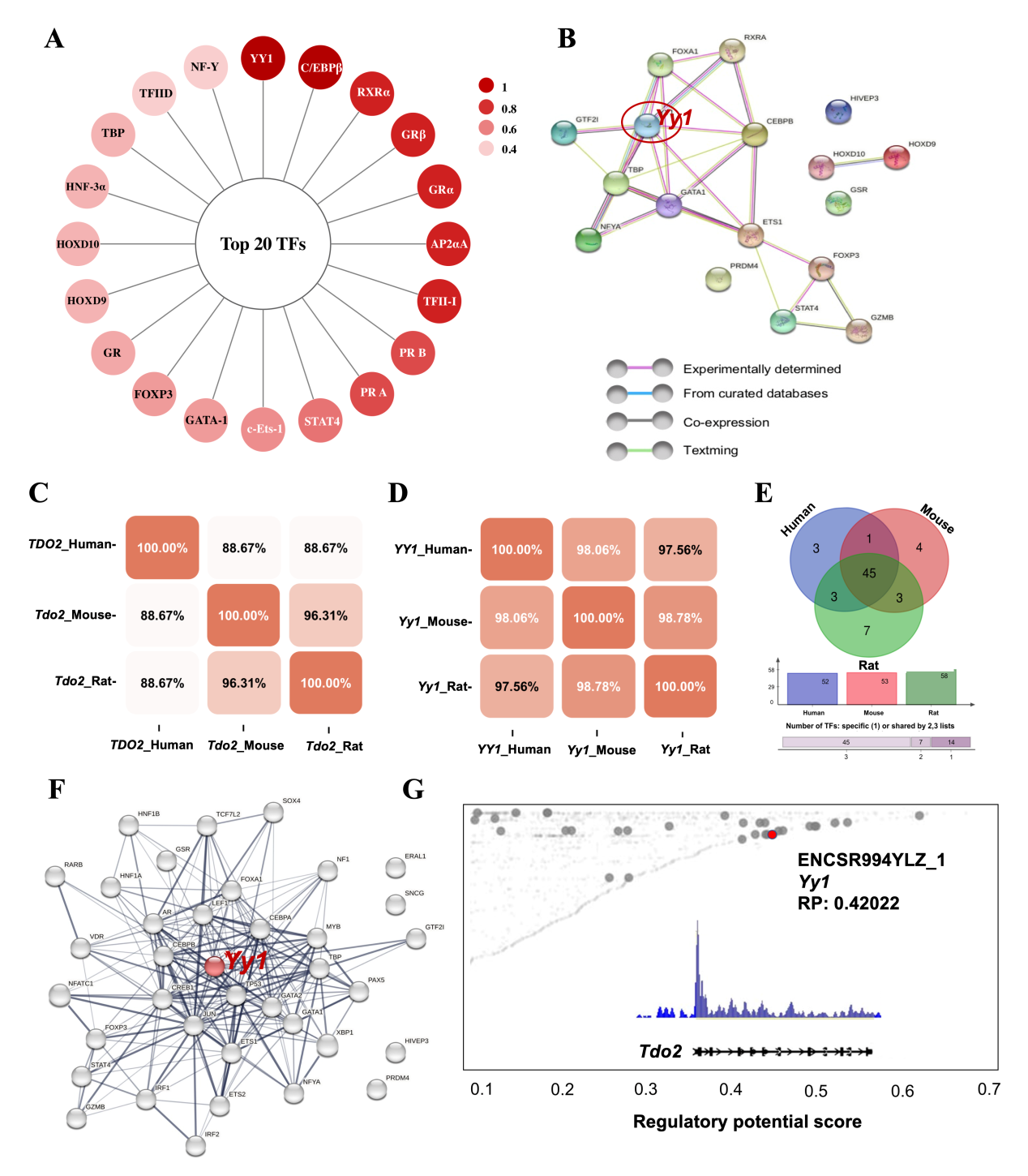
**

**Figure S7. YY1 was identified as a potential transcriptional factor of *TDO2*.** A) Top 20 predicted TFs binding with the enhancer and promoter of *Tdo2*. B) PPI network of these top 20 TFs. C, D) Sequence conservation analysis of TDO2 (C) and YY1 (D) among human, rat, and mouse based on the Uniprot database (https://www.uniprot.org/). E) Venn diagram of putative TFs of TDO2 from human (blue), mouse (red), and rat (green), respectively. F) Validating homologies of *Yy1* by constructing a PPI network of the overlapped 45 TFs. G) Identification of *Yy1* as a potential regulator of the *Tdo2* gene according to the Cistrome database (accession number: ENCSR994YLZ_1).

**
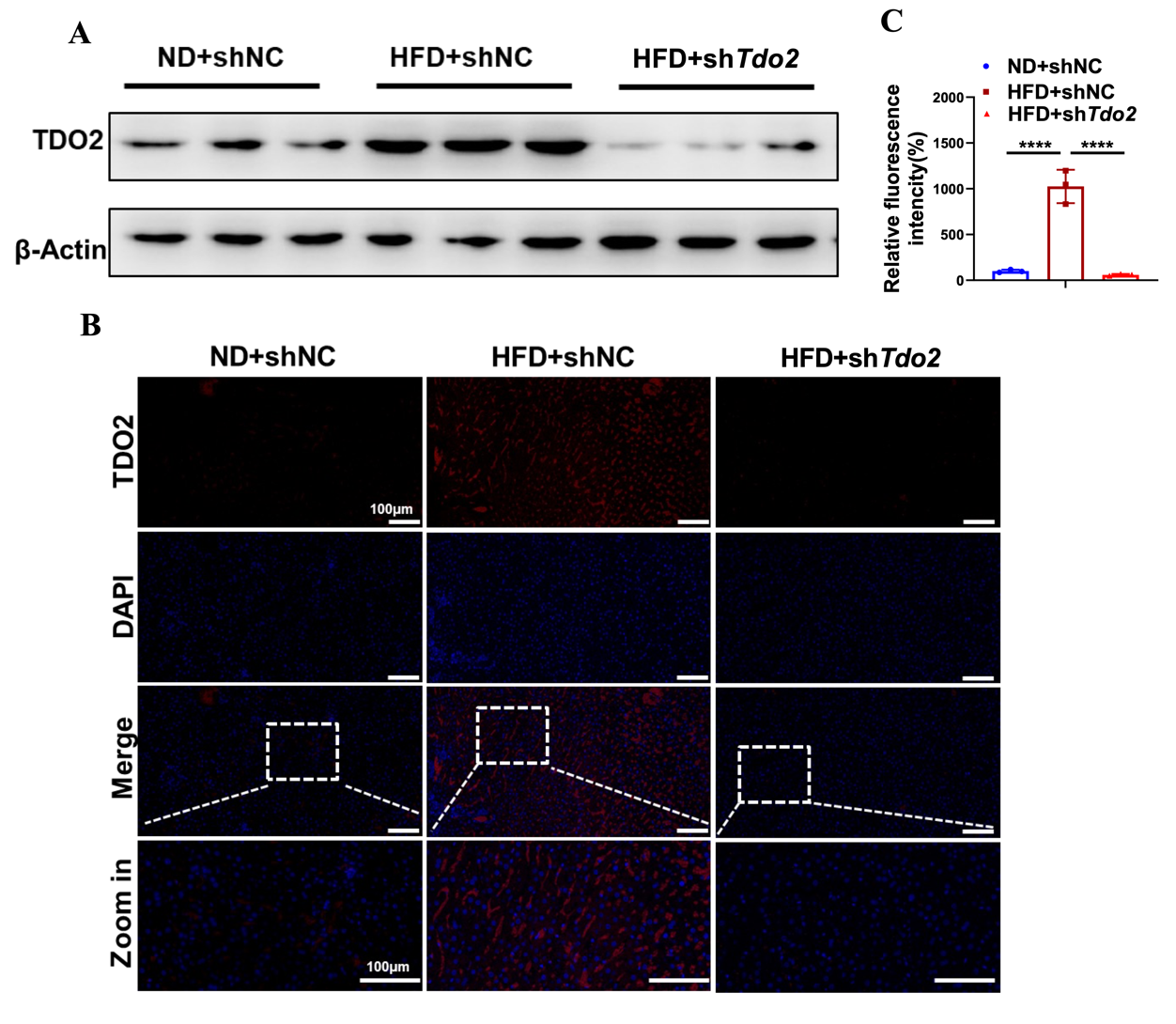
**

**Figure S8.** **Confirmation of *Tdo2* knockdown efficiency in liver tissues of HFD-induced MASLD rats.** A) The protein level of TDO2 in the liver tissues of rats infected with lentivirus particles of shNC or sh*Tdo2* through tail-vein injection and fed with ND or HFD was detected by Western blotting (n=3 per group). B, C) Representative immunofluorescence staining and quantification of TDO2 expression in the liver tissue sections of rats infected with lentivirus of shNC or sh*Tdo2* through tail-vein injection and fed with ND or HFD (n=3 per group, One-way ANOVA test, scale bar, 100 μm). The data in C were shown as mean ± SD. *P* values are indicated by * < 0.05; ** < 0.01; *** < 0.001; **** < 0.0001.


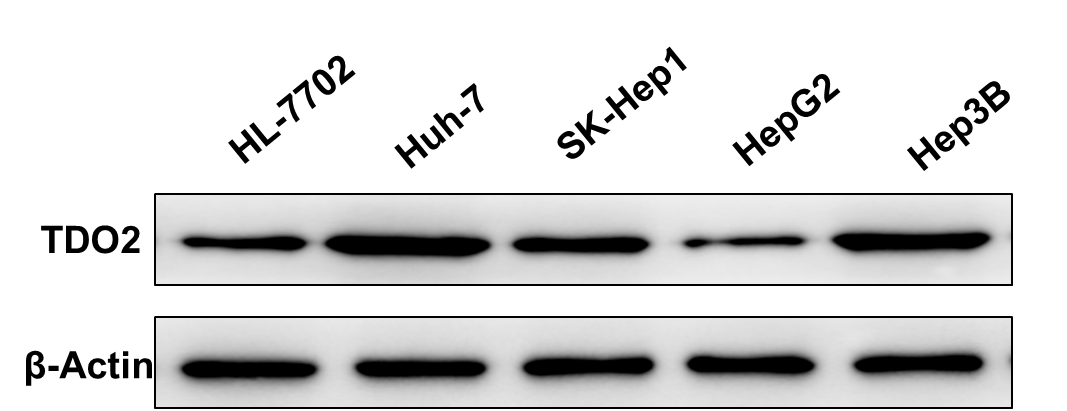


**Figure S9.** **Protein level of TDO2 in five human hepatic cell lines.** The protein level of TDO2 in five human hepatic cell lines (HL-7702, Huh-7, SK-Hep1, HepG2, Hep3B) was detected by Western blotting


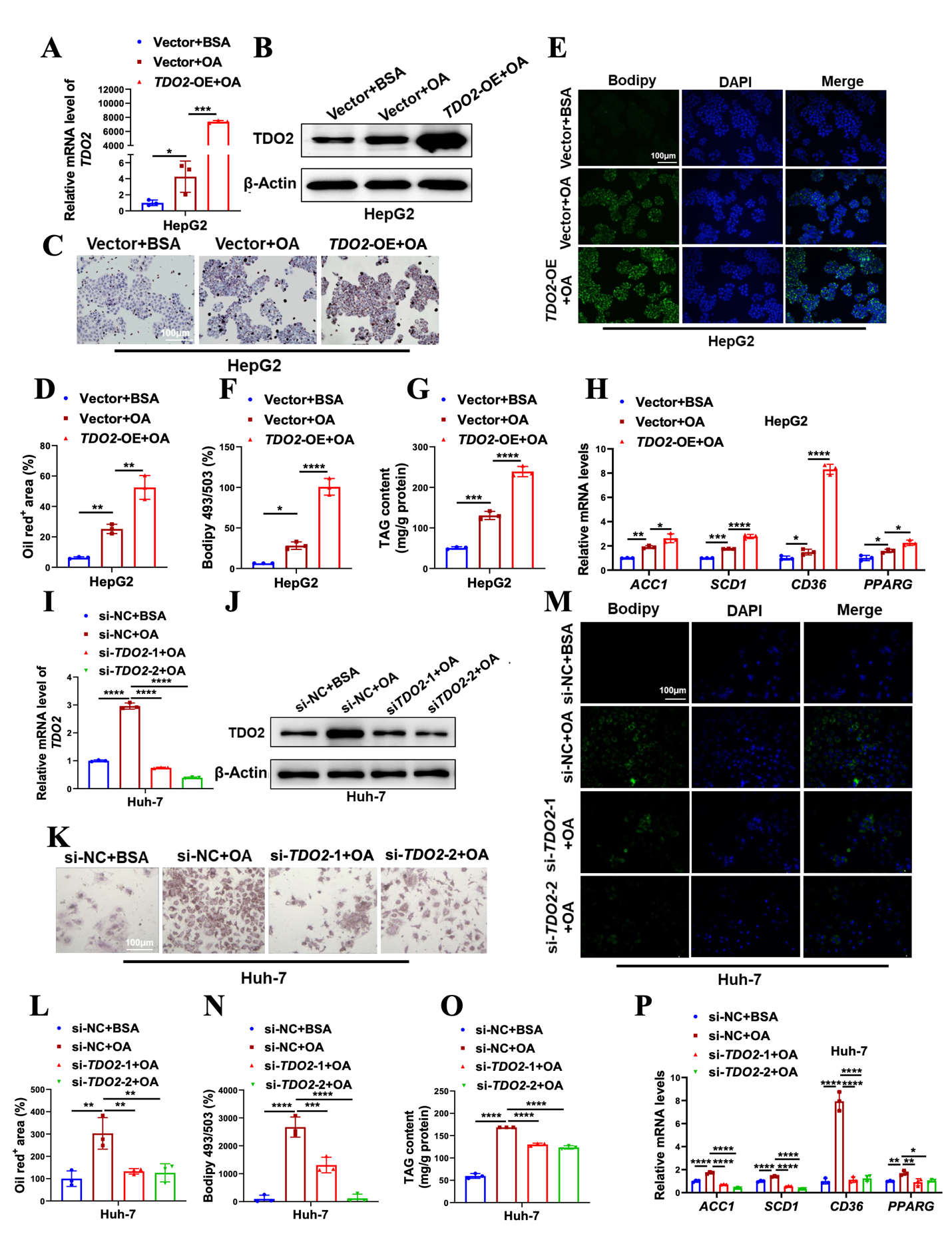


**Figure S10. *TDO2* exacerbated OA-induced cellular steatosis in hepatocytes.** A, B) The mRNA and protein levels of *TDO2* in HepG2 cells transfected with *TDO2* overexpression plasmid or empty vector with or without OA treatment (0.6 mM) were detected by qRT-PCR and Western blotting, respectively. C, D) Representative ORO staining of lipid droplets accumulation and quantitative analysis in HepG2 cells transfected with *TDO2* overexpression plasmid or empty vector with or without OA treatment (0.6 mM). Scale bar, 100 μm. E, F) Representative Bodipy 493/503 fluorescence staining of lipid droplets accumulation and quantitative analysis in HepG2 cells transfected with *TDO2* overexpression plasmid or empty vector with or without OA treatment (0.6 mM). Scale bar, 100 μm. G) Quantification of intracellular TG content in HepG2 cells transfected with *TDO2* overexpression plasmid or empty vector with or without OA treatment (0.6 mM). H) The mRNA levels of lipid transport and synthesis genes (*ACC1, SCD1, CD36* and *PPARG*) in HepG2 cells transfected with *TDO2* overexpression plasmid or empty vector with or without OA treatment (0.6 mM) were assessed by qRT-PCR. I, J) RNA and protein levels of *TDO2* in Huh-7 cells transfected with siRNAs against *TDO2* (si-*TDO2*-1 and si-*TDO2*-2) or negative control siRNA (si-NC) with or without OA treatment (0.6 mM) were detected by qRT-PCR and Western blotting, respectively. K, L) Representative Oil Red O staining of lipid droplets accumulation and quantitative analysis in Huh-7 cells transfected with siRNAs against *TDO2* (si-*TDO2*-1 and si-*TDO2*-2) or negative control siRNA (si-NC) with or without OA treatment (0.6 mM). Scale bar, 100 μm. M, N) Representative Bodipy 493/503 fluorescence staining of lipid droplets accumulation and quantitative analysis in Huh-7 cells transfected with siRNAs against *TDO2* (si-*TDO2*-1 and si-*TDO2*-2) or negative control siRNA (si-NC) with or without OA treatment (0.6 mM). Scale bar, 100 μm. O) Intracellular TG content in Huh-7 cells transfected with siRNAs against *TDO2* (si-*TDO2*-1 and si-*TDO2*-2) or negative control siRNA (si-NC) with or without OA treatment (0.6 mM). P) RNA levels of lipid transport and synthesis genes (*ACC1*, *SCD1*, *CD36* and *PPARG*) in Huh-7 cells transfected with siRNAs against *TDO2* (si-*TDO2*-1 and si-*TDO2*-2) or negative control siRNA (si-NC) with or without OA treatment (0.6 mM) were assessed by qRT-PCR. Results were shown as mean ± SD. *P* values are indicated by *** < 0.05; **** < 0.01; *** < 0.001; **** < 0.0001 (n=3 per group, One-way ANOVA test).


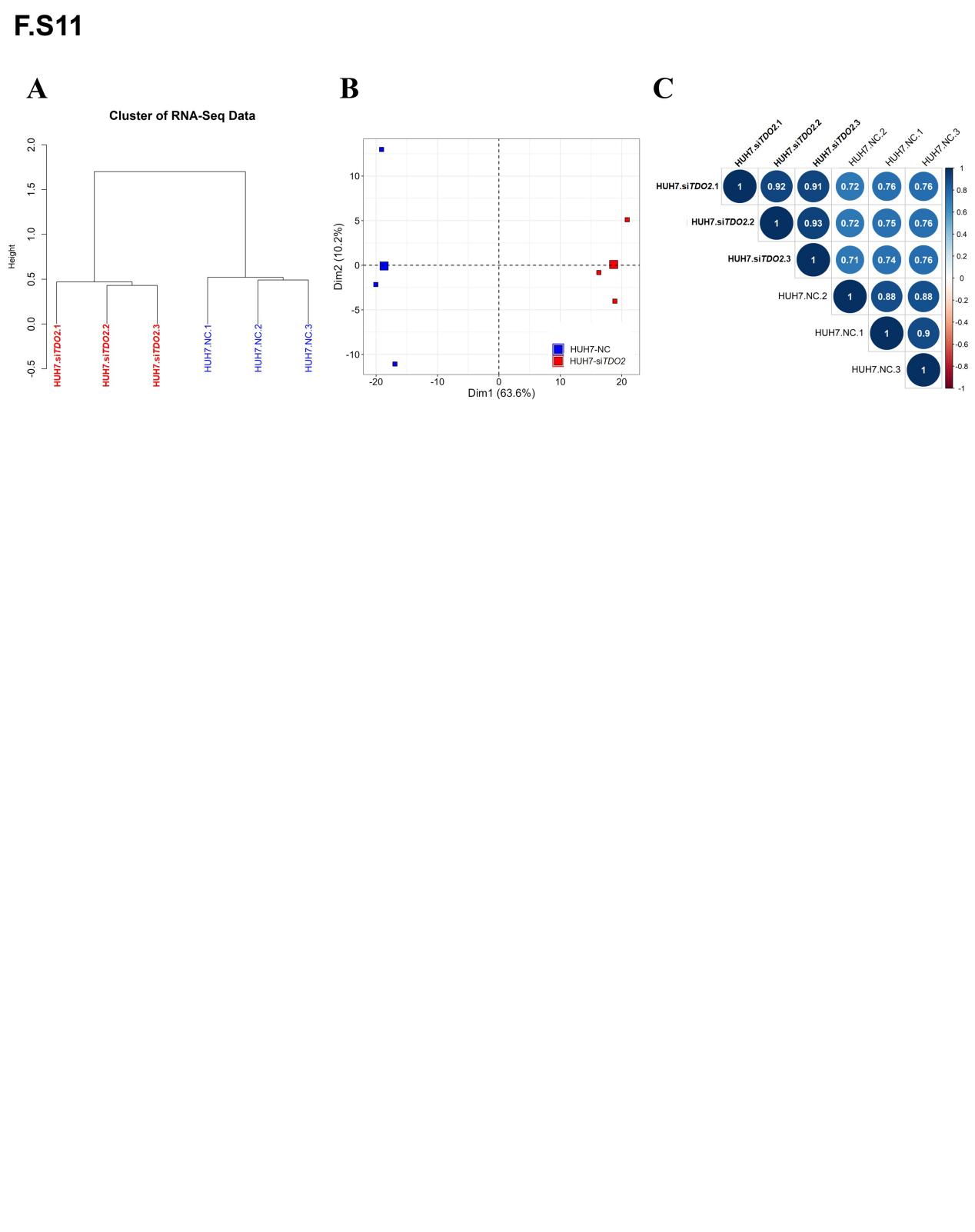


**Figure S11.** **Cluster analysis of** **RNA-Seq data from *TDO2*-depleted Huh-7 cells and its negative control counterparts in the upper chamber of co-culture system**. A) Neighbor Joining (NJ) tree clustering dendrogram illustrates the hierarchical relationships among the samples. B) Principal Component Analysis (PCA) of the samples. C) Spearman's correlation coefficient of the samples (n = 3 per group).


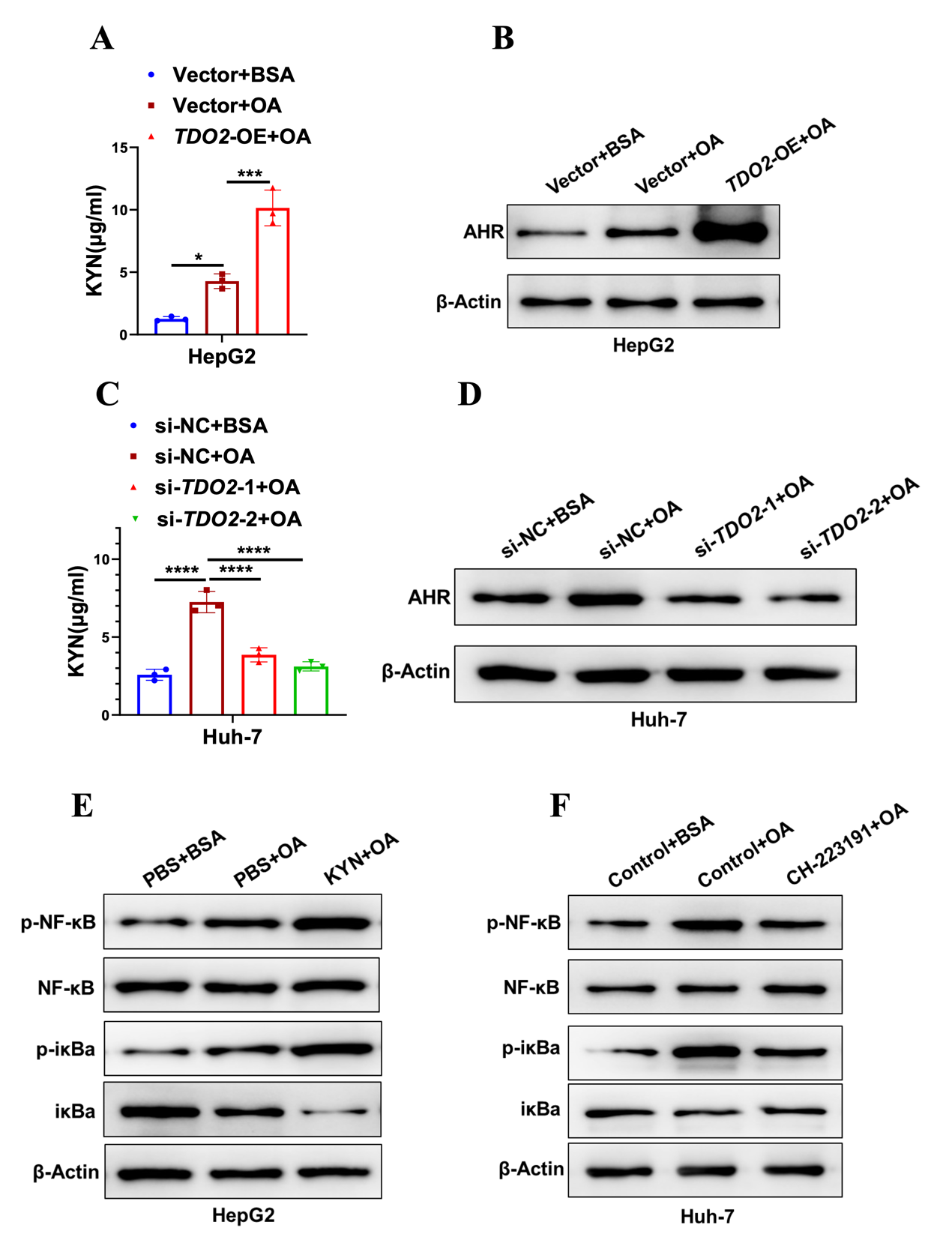


**Figure S12. KYN/AHR axis mediated TDO2-activated NF-κB signaling pathway.** A) KYN (Kynurenine) concentration in culture medium of HepG2 cells transfected with *TDO2* overexpression plasmid or empty vector with or without OA treatment (0.6 mM) was detected by ELISA. B) Protein level of AHR in HepG2 cells transfected with *TDO2* overexpression plasmid or empty vector with or without OA treatment (0.6 mM) was determined by Western blotting. C) KYN concentration in culture medium of Huh-7 cells transfected with siRNAs against *TDO2* (si-*TDO2*-1 and si-*TDO2*-2) or negative control siRNA (si-NC) with or without OA treatment (0.6 mM) was detected by ELISA.D) Protein levels of AHR in Huh-7 cells were transfected with siRNAs against TDO2 (si-TDO2-1 and si-TDO2-2) or negative control siRNA (si-NC) with or without OA treatment (0.6 mM) was determined by Western blotting. E) Protein levels of p-NF-κB, NF-κB p-iκBɑ and iκBɑ in KYN (30μM)- or PBS-treated HepG2 cells with or without OA treatment (0.6 mM) were determined by Western blotting. F) Protein levels of p-NF-κB, NF-κB p-iκBɑ and iκBɑ in CH-223191 (10μM)- or Control-treated Huh-7 cells with or without OA treatment (0.6 mM) were determined by Western blotting. Results were shown as mean ± SD. *P* values are indicated by *** < 0.05; **** < 0.01; *** < 0.001; **** < 0.0001 (n=3 per group, One-way ANOVA test).


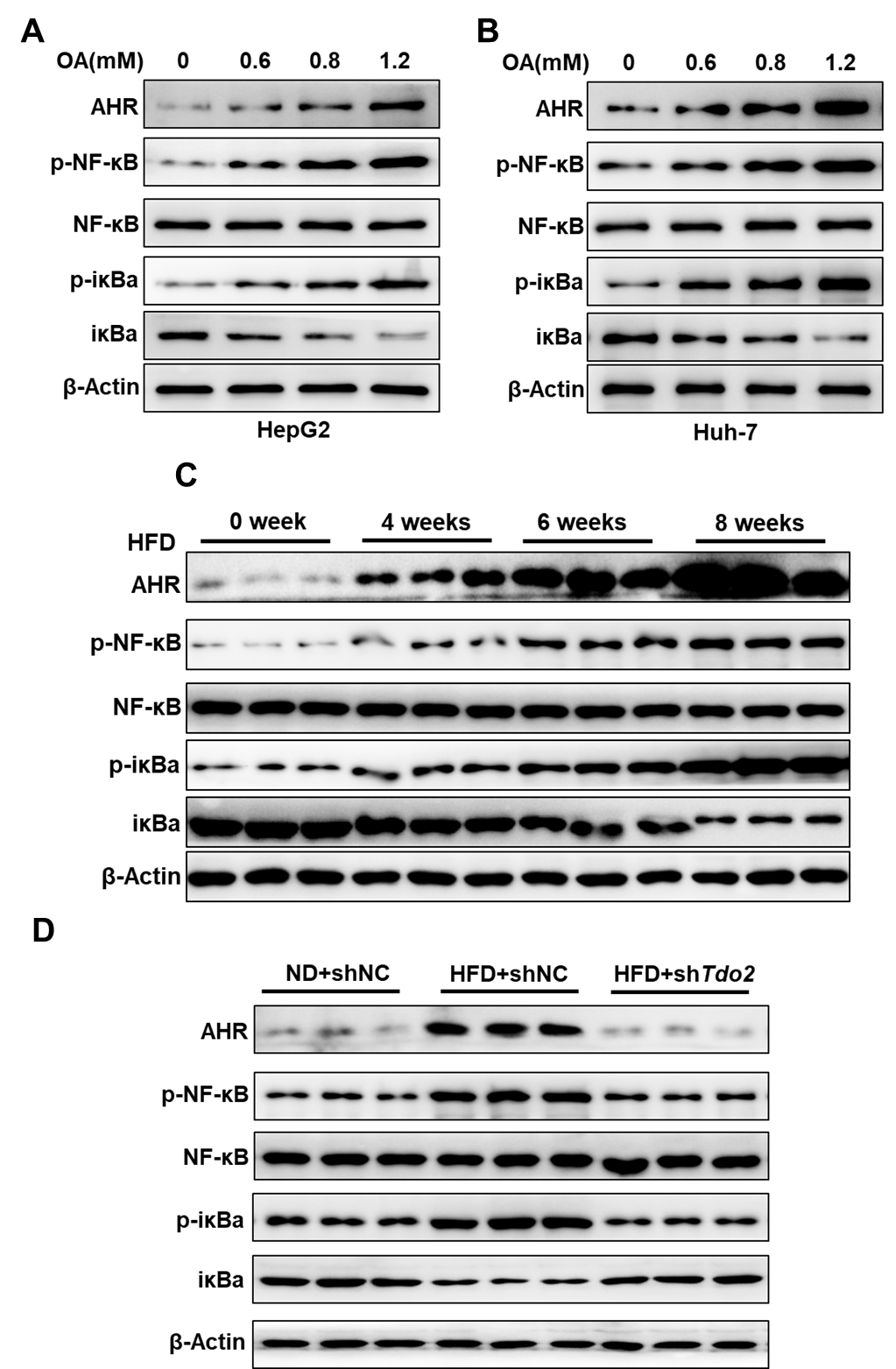


**Figure S13. Verification of the downstream signaling pathway of *TDO2*.** A, B) Protein levels of AHR, p-NF-κB, NF-κB p-iκBɑ and iκBɑ in HepG2 and Huh-7 cells treated with indicated dose of OA for 48 h were examined by Western blotting. C) Protein levels of AHR, p-NF-κB, NF-κB p-iκBɑ and iκBɑ in the liver tissues of rats fed with HFD for indicated weeks was evaluated by Western blotting (n = 3 per group). D) Protein levels of AHR, p-NF-κB, NF-κB p-iκBɑ and iκBɑ in the liver tissues of rats infected with lentivirus of shNC or sh*Tdo2* through tail-vein injection and fed with ND or HFD was detected by Western blotting (n=3 per group).


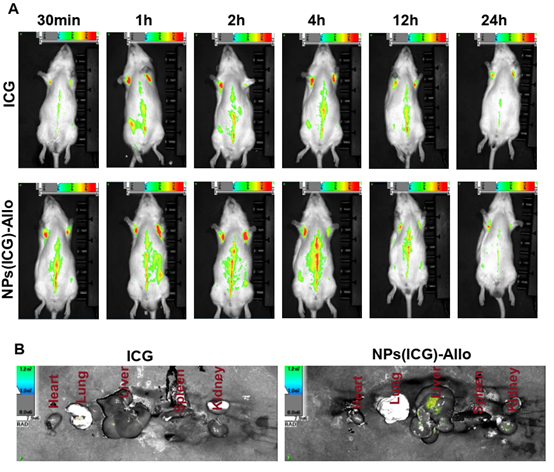


**Figure S14. The *in vivo* biodistribution image of ICG and NPs(ICG)-Allo in ND-fed** **rats.** A) Representative images of the ICG and NPs(ICG)-Allo *in vivo* at different time points (n=3 per group). B) Representative images of the ICG and NPs(ICG)-Allo distributed in the major organs (heart, lung, liver, spleen and kidney) collected at 24 h post injection (n=3 per group).


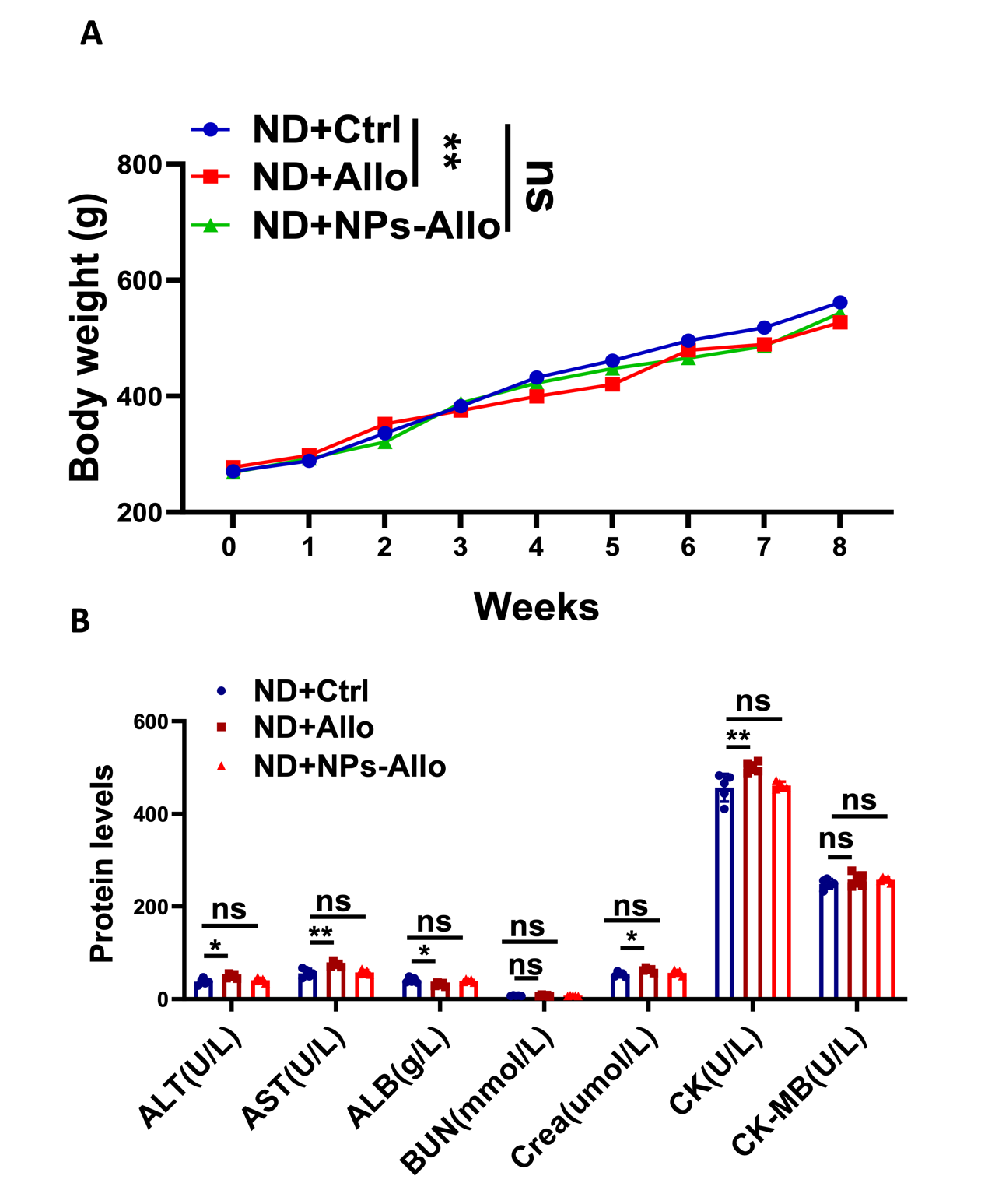


**Figure S15. Biocompatibility of** **free Allopurinol and NPs-Allo in rats.** A) Changes in body weight of ND-fed rats venously injected with free Allopurinol, NPs-Allo or negative control (n=3 per group, two-way ANOVA test). B) Biochemical analysis of the serum derived from rats fed with ND diet and venously injected with free Allopurinol, NPs-Allo or negative control, including liver function, renal function, and myocardial enzymes (n=5 per group, P-values were calculated with One-way ANOVA test ). Results are shown as mean ± SD. *P* values are indicated by * < 0.05; ** < 0.01.

**Supplemental Tables**

**Table S1. Primers used for qPCR.**

| Gene | Forward primer sequence | Reverse primer sequence | Species |
| --- | --- | --- | --- |
| *β-Actin* | CCTCGCCTTTGCCGATCC | GGATCTTCATGAGGTAGTCAGTC | Human |
| *TDO2* | CCGTAGAAGGCAGCGAAGAA | GCAGGTAGTTCCCATAGATAAGACC | Human |
| *YY1* | AAGAGCGGCAAGAAGAGTTAC | CAACCACTGTCTCATGGTCAATA | Human |
| *ACC1* | ATGTCTGGCTTGCACCTAGTA | CCCCAAAGCGAGTAACAAATTCT | Human |
| *SCD1* | GGGGGTGTGCTGACAACTTA | AGGCCCCTTTTTCTACCAGC | Human |
| *CD36* | GGCTGTGACCGGAACTGTG | AGGTCTCCAACTGGCATTAGAA | Human |
| *PPARG* | CCAGAAGCCTGCATTTCTGC | GTGTCAACCATGGTCATTTCGTT | Human |
| *HLA-DR* | GCTTGAAGAATTTGGACG | AGGAAGGGGAGATAGTGG | Human |
| *TNF-α* | CTGGGCAGGTCTACTTTGGG | CTGGAGGCCCCAGTTTGAAT | Human |
| *β-actin* | GTTGACATCCGTAAAGAC | ACCAATCCACACAGAGTA | Rattus |
| *Tdo2* | TAAACAGAGCCAGCAAAG | GAAGATGACCACCACACG | Rattus |
| *Cd36* | CTATTGGGAAAGTTATTGCGA | GGGTTCTGGAGTGGGGA | Rattus |
| *Cpt1α* | AGGTCTGGCTCTACCACGAT | CGCATCCAGGGACTGCTTAT | Rattus |
| *Fasn* | TGTTATCACCCGACTTCCTCTG | GCTGAATACGACCACGCACTA | Rattus |
| *Pparα* | GTCCTCTGGTTGTCCCCTTG | TGGGGAGAGAGGACAGATGG | Rattus |

**Table S2. Sequences of siRNAs, shRNAs, and primers for cloning, ChIP assays.**

| Gene | Application | Sequence | Species |
| --- | --- | --- | --- |
| si-*TDO2*-1 | Cell transfection | AUACCUUGUACCUAUCACUCACAGU  ACUGUGAGUGAUAGGUACAAGGUAU | Human |
| si-*TDO2*-2 | Cell transfection | CCCGACACUGGAUACCGAAGAUGAA  UUCAUCUUCGGUAUCCAGUGUCGGG | Human |
| si-*YY1*-1 | Cell transfection | CGAGGAUCAGAUUCUCAUC  GAUGAGAAUCUGAUCCUCG | Human |
| si-*YY1*-2 | Cell transfection | GAACUCACCUCCUGAUUAU  AUAAUCAGGAGGUGAGUUC | Human |
| *Tdo2*-shRNA1-F | plasmid  construction | ccggGCTTAGAGCCACATGGATTCAggatccTGAATCCATGTGGCTCTAAGCtttttg | Rattus |
| *Tdo2*-shRNA1-R | plasmid  construction | aattcaaaaaGCTTAGAGCCACATGGATTCAggatccTGAATCCATGTGGCTCTAAGC | Rattus |
| *Tdo2*-shRNA2-F | plasmid  construction | ccggCCCGACACTGGATACCAAAGATGAAggatccTTCATCTTTGGTATCCAGTGTCGGGtttttg | Rattus |
| *Tdo2*-shRNA2-R | plasmid  construction | aattcaaaaaCCCGACACTGGATACCAAAGATGAAggatccTTCATCTTTGGTATCCAGTGTCGGG | Rattus |
| *TDO2*-clone-F | plasmid  construction | CTAGCTAGCATGAGTGGGTGCCCATTTTTAG | Human |
| *TDO2*-clone-R | plasmid  construction | CCGCTCGAGTTAATCTGATTCATCACTGCTG | Human |
| *YY1*-clone-F | plasmid  construction | CTAGCTAGCATGGCCTCGGGCGACACCC | Human |
| *YY1*-clone-R | plasmid  construction | CCGCTCGAGTCACTGGTTGTTTTTGGC | Human |
| *TDO2* promoter-clone-F | plasmid  construction | CTAGCTAGCTATTGGCAAATGATGACCAG | Human |
| *TDO2*  promoter-clone-R | plasmid  construction | CCGCTCGAGTCCCAGATGTTCTACAGCTG | Human |
| *TDO2* promoter-mut-clone-F | plasmid  construction | CAAAATGCAAGTATGGTAGAT | Human |
| *TDO2* promoter-mut-clone-R | plasmid  construction | ATCTACCATACTTGCATTTTG | Human |
| *TDO2* enhancer-F | CHIP- qPCR | AGATTGATTCCAACTATGCTTC | Human |
| *TDO2* enhancer-R | CHIP- qPCR | TTGTTCTCCCCTTTTACTACGG | Human |

**Table S3. Antibodies.**

| Antibodies | Assay | Origin | Dilution |  |
| --- | --- | --- | --- | --- |
| anti-TDO2 | WB | BBI  D199153 | 1:500 |  |
| anti-YY1 | WB | Proteintech  66281-1-Ig | 1:5000 |  |
| anti-p-NF-κB | WB | CST  3033 | 1：1000 |  |
| anti-NF-κB | WB | CST  8242 | 1：1000 |  |
| anti-p-iκBa | WB | CST  2859 | 1：1000 |  |
| anti-iκBa | WB | CST  4812 | 1：1000 |  |
| anti-AHR | WB | CST  83200 | 1：1000 | |
| anti-β-actin | WB | Proteintech  66009-1-Ig | 1:20000 |  |
| anti-TDO2 | IF | Abcam  ab259359 | 1:500 |  |
| anti-INOS | IF | Abcam  Ab283655 | 1:50 |  |
| anti-hCD86-APC | FC | Biolegend  374208 | 1:100 |  |

**Table S4. The function of top 20 differentially H3K27ac peak–genes between ND and MASLD rats.**

| Peak | Target gene | ChIP(log_2_FoldChange) | *P* value (ChIP) | RNA(log_2_FoldChange) | *P* value (RNA) | Corelation | *P* value | Function |
| --- | --- | --- | --- | --- | --- | --- | --- | --- |
| 2:180025335-180029021 | *Tdo2* | 2.345122109 | 3.48E-11 | 15.15114162 | 2.41E-37 | 0.975865282 | 8.67E-04 | Tryptophan 2,3-dioxygenase (TDO2), a heme enzyme catabolyzing tryptophan to kynurenine that constitutively expressed in the liver, which is involved in inflammation-related diseases. High TDO2 levels in the synovium is correlated with pro-inflammatory cytokines and severity of OA. |
| 14:22083483-22087917 | *Sult1e1* | 1.72056292 | 5.61E-12 | 16.07131038 | 1.56E-41 | 0.995347344 | 3.24E-05 | A member of sulfotransferase family 1E participating in the inflammatory response and lipid metabolism through PPARγ regulation. |
| 5:79055283-79063048 | *Ambp* | 1.112112875 | 6.93E-10 | 15.11574512 | 1.73E-218 | 0.977490946 | 7.54E-04 | AMBP (alpha-1-microglobulin/bikunin precursor), inhibiting oxidative modification of low-density lipoprotein (LDL) and lipids, attenuating oxidized LDL-driven cell death. |
| 14:22083483-22087917 | *Sult1b1* | 1.72056292 | 5.61E-12 | 13.48459954 | 8.12E-30 | 0.992651226 | 8.08E-05 | SULT1B1(sulfotransferase family 1B member 1), involving in several processes, including 3'-phosphoadenosine 5'-phosphosulfate metabolic process; ethanol catabolic process. |
| 2:150776814-150778643 | *Aadac* | 1.713965606 | 9.73E-08 | 12.71373706 | 1.80E-26 | 0.97727705 | 7.69E-04 | AADAC (arylacetamide deacetylase) has triglyceride lipase activity, involving in lipid metabolic and positive regulation of triglyceride catabolic and promotes TG lipolysis. |
| 5:77246433-77247383 | *Aabr07048463.1* | 1.577326752 | 9.39E-05 | 12.55833777 | 8.19E-26 | 0.971533599 | 1.20E-03 | / |
| 2:61058421-61061519 | *Agxt2* | 1.346930862 | 3.11E-08 | 12.21967849 | 5.74E-23 | 0.983101821 | 4.26E-04 | AGXT2 is a mitochondrial aminotransferase that specifically expressed in the liver and kidney. Alterion of AGXT2 expression modulates the progression of liver fibrosis and steatosis. |
| 17:69440705-69442161 | *Akr1c12* | 1.61274393 | 2.93E-05 | 11.71904446 | 3.00E-72 | 0.981898099 | 4.89E-04 | AKR1C12(aldo-keto reductase family 1, member C12), highly expressed in the liver, and involves in steroid metabolic. |
| 20:28831582-28834049 | *Pla2g12b* | 1.529272975 | 0.00101879 | 11.05447949 | 4.00E-20 | 0.968779231 | 1.45E-03 | The gene encodes the group XIIB secreted phospholipase A2, and involves in cholesterol homeostasis, lipid catabolic and triglyceride homeostasis. |
| 17:69440705-69442161 | *Akr1c1* | 1.61274393 | 2.93E-05 | 10.94196882 | 6.40E-18 | 0.976918978 | 7.93E-04 | AKR1C1(aldo-keto reductase family 1, member C1) exerts hydroxysteroid dehydrogenase (HSD) activity which is association with the reduction and inactivation of steroid hormones. |
| 2:180022886-180024798 | *Rf00181* | 2.975079789 | 4.81E-11 | 8.981777302 | 1.04E-10 | 0.98514023 | 3.30E-04 | / |
| 7:124256880-124259097 | *Cyp2d5* | 1.213483404 | 0.000300439 | 10.52209111 | 2.28E-93 | 0.977982697 | 7.22E-04 | Cyp2d5(cytochrome P450, family 2, subfamily d, polypeptide 5), an integral component of membrane that active in cytoplasm and intracellular membrane-bounded organelle. |
| 14:22323743-22324239 | *Ugt2b15* | 2.069285007 | 7.20E-05 | 9.125202099 | 3.26E-11 | 0.975326072 | 9.06E-04 | This gene encodes a glycosyltransferase that is invovled in the metabolism and elimination of toxic compounts, both endogenous and of xenobiotic origin. |
| 17:69440705-69442161 | *Rgd1564865* | 1.61274393 | 2.93E-05 | 9.56163621 | 1.30E-12 | 0.95908884 | 2.48E-03 | / |
| 3:152506786-152509376 | *Romo1* | 1.270933443 | 0.000343173 | 8.922983444 | 4.83E-59 | 0.980012955 | 5.95E-04 | ROMO1 (Reactive Oxygen Species Modulator 1) is a Protein Coding gene that essential for the proliferation cells. |
| 7:124256880-124259097 | *Cyp2d1* | 1.213483404 | 0.000300439 | 6.765483487 | 8.16E-56 | 0.959701909 | 2.40E-03 | CYP2D is expressed in rat brain regions and enzyme activity correlates with protein and messenger ribonucleic acid (mRNA) levels. |
| 12:19113441-19116198 | *Aabr07035541.2* | 1.508567667 | 1.36E-05 | 6.391880098 | 1.18E-06 | 0.990731178 | 1.28E-04 | / |
| 1:213498092-213504032 | *Slc25a22* | 2.046494469 | 3.43E-09 | 5.840613842 | 2.40E-35 | 0.97786063 | 7.30E-04 | / |
| 6:26074140-26075523 | *Khk* | 1.177095668 | 0.009487882 | 6.14582176 | 7.10E-22 | 0.988984297 | 1.81E-04 | This gene encodes ketohexokinase that catalyzes conversion of fructose to fructose-1-phosphate, and involves in fructose-induced accumulation of triglycerides in hepatocytes in vitro and in vivo. |
| 20:28831582-28834049 | *Oit3* | 1.529272975 | 0.00101879 | 5.287140103 | 1.46E-14 | 0.964165741 | 1.90E-03 | OIT3 (Oncoprotein Induced Transcript 3),a novel marker of M2 macrophages that mediates macrophage polarization and promotes hepatocellular carcinoma progression. |
| 12:38031716-38033920 | *Eif2b1* | -1.065807998 | 9.44E-05 | -5.881961406 | 7.35E-07 | 0.991587556 | 1.06E-04 | / |
| 8:49489616-49492843 | *Il10ra* | -1.433052076 | 1.39E-10 | -5.611194643 | 2.82E-06 | 0.997400271 | 1.01E-05 | The protein encoded by this gene is a receptor for interleukin 10 that involves in negative regulation of inflammatory response. |
| 12:38031716-38033920 | *Orai1* | -1.065807998 | 9.44E-05 | -6.081615151 | 2.95E-07 | 0.992884557 | 7.58E-05 | / |
| 3:120893075-120894134 | *Anapc1* | -2.042441441 | 2.77E-07 | -5.413614797 | 1.33E-10 | 0.990722214 | 1.29E-04 | / |
| 3:55647749-55649487 | *Dhrs9* | -2.631474071 | 0.00037972 | -5.030528593 | 0.000411157 | 0.992122892 | 9.28E-05 | DHRS9 (Dehydrogenase/Reductase 9) is a Protein Coding gene involves in retinoic acid biosynthetic process. |
| 8:49489616-49492843 | *Kmt2a* | -1.433052076 | 1.39E-10 | -6.234877484 | 1.10E-13 | 0.998806228 | 2.14E-06 | This gene encodes a transcriptional coactivator that plays an essential role in regulating gene expression during early development and hematopoiesis. |
| 14:83160537-83161993 | *Slc5a1* | -1.557963029 | 3.15E-05 | -6.11781529 | 2.53E-07 | 0.994119393 | 5.18E-05 | This gene encodes a member of the sodium-dependent glucose transporter protein (SGLT) family. This protein is found mainly in the intestinal tract and the kidneys. It spans the membrane of cells and moves (transports) two sugars called glucose and galactose from outside the cell to inside the cell. |
| 8:49489616-49492843 | *Cbl* | -1.433052076 | 1.39E-10 | -6.430106108 | 6.02E-08 | 0.994778219 | 4.08E-05 | This gene is a member of the cobalamin transport protein family that encodes a glycoprotein secreted by parietal cells of the gastric mucosa. |
| 14:83160537-83161993 | *Limk2* | -1.557963029 | 3.15E-05 | -6.549018703 | 3.39E-08 | 0.992763102 | 7.84E-05 | LIM kinase 2 (LIMK2), a members of the LIM kinase family that play a crucial role in the regulation of cytoskeleton dynamics by controlling actin filaments and microtubule turnover, especially through the phosphorylation of cofilin, an actin depolymerising factor. |
| 14:83160537-83161993 | *Nsd2* | -1.557963029 | 3.15E-05 | -6.662826888 | 1.77E-08 | 0.992256457 | 8.97E-05 | / |
| 5:153122051-153126632 | *Rps6ka1* | -1.308908589 | 1.54E-09 | -6.93566847 | 6.05E-09 | 0.99080562 | 1.26E-04 | Rps6ka1(ribosomal protein S6 kinase polypeptide 1), involves in positive regulation of hepatic stellate cell activation and hepatocyte proliferation. |
| 1:219741559-219747215 | *Loc102555167* | -1.619419935 | 8.42E-15 | -6.819617767 | 8.51E-09 | 0.990884646 | 1.24E-04 | / |
| 13:36179952-36182219 | *Dbi* | -1.733024236 | 3.11E-07 | -6.731070698 | 1.69E-08 | 0.992697878 | 7.98E-05 | This gene encodes diazepam binding inhibitor, a protein that is regulated by hormones and is involved in fatty acid metabolic process, triglyceride metabolic and positive regulation of lipid biosynthetic. |
| 12:38031716-38033920 | *Mlxip* | -1.065807998 | 9.44E-05 | -7.474838062 | 2.54E-10 | 0.991551236 | 1.07E-04 | This gene encodes a protein that functions as part of a heterodimer to activate transcription. The encoded protein forms a heterodimer with Max-like protein X (MLX) to regulate lipid storage. |
| 1:177006246-177010630 | *Mical2* | -2.497270752 | 8.40E-38 | -6.215249511 | 1.53E-09 | 0.994113788 | 5.19E-05 | The protein encoded by this gene is a monooxygenase that enhances depolymerization of F-actin and is therefore involved in cytoskeletal dynamics. |
| 9:45768855-45770715 | *Rnf149* | -1.73181107 | 8.61E-09 | -7.082901578 | 2.90E-09 | 0.994686261 | 4.23E-05 | Rnf149(ring finger protein 149), an E3 ligase that involes in inflammatory response. |
| 8:49489616-49492843 | *Bcl9l* | -1.433052076 | 1.39E-10 | -8.811209646 | 9.01E-14 | 0.996797037 | 1.54E-05 | BCL9L, a homologous coactivators of the β-catenin transcription factor complex involved in negative regulation of transforming growth factor beta receptor signaling pathway; positive regulation of epithelial to mesenchymal transition; and positive regulation of transcription by RNA polymerase II. |
| 12:38031716-38033920 | *Ncor2* | -1.065807998 | 9.44E-05 | -9.449375358 | 1.51E-15 | 0.995718356 | 2.75E-05 | This gene encodes a nuclear receptor co-repressor that mediates transcriptional silencing of certain target genes. |
| 12:23905549-23906719 | *Cux1* | -3.770262879 | 4.20E-05 | -7.042726993 | 2.98E-09 | 0.993654107 | 6.03E-05 | CUX1 belongs to the homeodomain transcription factor family that involves in various physiological events including tissue development, cell proliferation, differentiation and migration, and DNA damage response. |
| 1:41166132-41172948 | *Akap12* | -2.122652281 | 1.25E-21 | -9.786838157 | 4.83E-16 | 0.99242988 | 8.57E-05 | A-kinase anchor protein 12 (AKAP12) also known as Gravin and SSeCKS, an in vivo and in vitro protein kinase C substrate that involves in the regulation of immune-related signaling pathways. |

**Table S5. The function of 121 uniform genes among human, mice and rats.**

| gene | Mouse | | Rat | | Human | | Function |
| --- | --- | --- | --- | --- | --- | --- | --- |
|  | log_2_FoldChange | *P* value | log_2_FoldChange | *P* value | log_2_FoldChange | *P* value |  |
| *Abat* | 2.57 | 1.65E-02 | 8.24 | 6.19E-41 | 3.57 | 1.74E-62 | This gene catalyzes the conversion of gamma-aminobutyrate and L-beta-aminoisobutyrate to succinate semialdehyde and methylmalonate semialdehyde. |
| *Abcg5* | 2.44 | 1.23E-02 | 8.01 | 1.59E-07 | 9.17 | 2.54E-179 | Transporter that appears to play an indispensable role in the selective transport of the dietary cholesterol in and out of the enterocytes and in the selective sterol excretion by the liver into bile. |
| *Acaa2* | 2.24 | 1.94E-02 | 2.42 | 9.28E-85 | 2.65 | 1.33E-73 | Abolishes BNIP3-mediated apoptosis and mitochondrial damage. |
| *Acadl* | 5.28 | 1.38E-03 | 2.57 | 6.07E-116 | 8.47 | 1.42E-100 | This gene has acyl-CoA dehydrogenase and fatty acid beta-oxidation regulation function. |
| *Acadm* | 3.79 | 1.51E-03 | 2.08 | 1.11E-55 | 2.17 | 9.14E-78 | acyl-CoA dehydrogenase, fatty acid beta-oxidation. |
| *Acot13* | 3.00 | 2.74E-03 | 3.00 | 8.74E-20 | 1.53 | 1.45E-22 | Catalyze the hydrolysis of acyl-CoAs to the free fatty acid and coenzyme A (CoASH). |
| *Acox2* | 3.62 | 8.47E-04 | 2.39 | 2.34E-02 | 4.04 | 7.09E-30 | Peroxisomal acyl-CoA oxidase, acting on the CoA-esters of bile acid intermediates and pristanic acid, expressed in liver and extrahepatic tissues. |
| *Acsm3* | 3.30 | 1.62E-03 | 4.16 | 2.22E-21 | 5.60 | 3.08E-258 | acyl-CoA synthetase medium-chain family member 3(ACSM3), predicted to be involved in fatty acid biosynthetic process. |
| *Afm* | 2.98 | 3.15E-03 | 16.28 | 3.94E-43 | 10.69 | 3.77E-270 | Vitamin E binding protein. May be involved in the regulation and transport of vitamin E at the blood-brain barrier |
| *Agt* | 3.36 | 1.56E-03 | 5.93 | 5.37E-249 | 2.32 | 2.12E-16 | Essential component of the renin-angiotensin system (RAS), a potent regulator of blood pressure, body fluid and electrolyte homeostasis. |
| *Agxt* | 2.60 | 8.00E-03 | 5.87 | 1.47E-10 | 11.11 | 0.00E+00 | alanine--glyoxylate aminotransferase (AGXT), This gene encodes alanine-glyoxylate aminotransferase, which catalyzes the interconversion of L-alanine and glyoxylate to pyruvate and glycine. |
| *Ambp* | 2.36 | 1.73E-02 | 15.12 | 1.73E-218 | 8.23 | 7.19E-52 | / |
| *Angptl6* | 4.24 | 7.64E-04 | 5.02 | 1.19E-39 | 6.96 | 2.50E-70 | May counteract high-fat diet-induced obesity and related insulin resistance through increased energy expenditure. |
| *Apoa2* | 2.10 | 2.62E-02 | 15.12 | 4.59E-109 | 11.20 | 0.00E+00 | May stabilize HDL (high density lipoprotein) structure by its association with lipids, and affect the HDL metabolism. |
| *Apoa4* | 9.12 | 3.13E-07 | 16.44 | 1.31E-41 | 6.28 | 3.18E-16 | May have a role in chylomicrons and VLDL secretion and catabolism. Required for efficient activation of lipoprotein lipase by ApoC-II; potent activator of LCAT. Apoa-IV is a major component of HDL and chylomicrons. |
| *Apoa5* | 2.63 | 7.12E-03 | 10.44 | 1.74E-105 | 10.15 | 3.29E-301 | Important determinant of plasma triglyceride (TG) levels by both being a potent stimulator of apo-CII lipoprotein lipase (LPL) TG hydrolysis and a inhibitor of the hepatic VLDL-TG production rate. |
| *Apoc2* | 3.88 | 6.56E-04 | 15.82 | 1.82E-30 | 11.15 | 0.00E+00 | Component of the very low density lipoprotein (VLDL) fraction in plasma, and is an activator of several triacylglycerol lipases. |
| *Apoc3* | 2.24 | 1.74E-02 | 9.13 | 7.75E-25 | 10.86 | 0.00E+00 | Inhibits lipoprotein lipase and hepatic lipase and decreases the uptake of lymph chylomicrons by hepatic cells. This suggests that it delays the catabolism of triglyceride-rich particles. |
| *Apoc4* | 2.24 | 2.06E-02 | 9.93 | 2.81E-184 | 10.96 | 1.48E-193 | This gene predicted to be involved in positive regulation of sequestering of triglyceride and triglyceride homeostasis, and to be part of high-density lipoprotein particle and very-low-density lipoprotein particle. |
| *Apoh* | 3.88 | 5.28E-04 | 16.69 | 2.53E-59 | 10.44 | 0.00E+00 | May prevent activation of the intrinsic blood coagulation cascade by binding to phospholipids on the surface of damaged cells. |
| *Aqp9* | 2.75 | 6.66E-03 | 7.12 | 2.74E-122 | 11.62 | 1.74E-286 | Forms a channel with a broad specificity, also permeable to urea and glycerol. |
| *Asgr1* | 2.58 | 7.70E-03 | 8.28 | 3.59E-10 | 8.81 | 1.27E-86 | Mediates the endocytosis of plasma glycoproteins to which the terminal sialic acid residue on their complex carbohydrate moieties has been removed. |
| *Aspg* | 4.46 | 1.29E-03 | 7.18 | 1.29E-02 | 7.65 | 1.48E-82 | Exhibits lysophospholipase, transacylase, PAF acetylhydrolase and asparaginase activities. |
| *Atf5* | 4.69 | 8.25E-05 | 2.96 | 2.13E-143 | 4.08 | 3.18E-66 | Transcriptional activator which binds the cAMP response element (CRE), a sequence present in many viral and cellular promoters and blocks the differentiation of neuroprogenitor cells into neurons. |
| *Atox1* | 3.41 | 1.25E-03 | 3.81 | 1.64E-64 | 1.17 | 4.32E-17 | Binds and deliver cytosolic copper to the copper ATPase proteins. May be important in cellular antioxidant defense. |
| *Baat* | 2.15 | 3.46E-02 | 7.43 | 2.20E-107 | 5.12 | 8.11E-15 | Involved in bile acid metabolism. In liver hepatocytes catalyzes the second step in the conjugation of C24 bile acids (choloneates) to glycine and taurine before excretion into bile canaliculi. In vitro, catalyzes the hydrolysis of long- and very long-chain saturated acyl-CoAs to the free fatty acid and coenzyme A (CoASH), and conjugates glycine to these acyl-CoAs. |
| *Blvrb* | 2.22 | 2.02E-02 | 2.51 | 1.41E-88 | 1.05 | 2.31E-14 | Contributes to heme catabolism and metabolizes linear tetrapyrroles |
| *C1qa* | 3.23 | 1.91E-03 | 2.17 | 1.35E-10 | 11.37 | 1.44E-247 | C1q associates with the proenzymes C1r and C1s to yield C1, the first component of the serum complement system. |
| *Ccs* | 2.17 | 2.38E-02 | 2.36 | 3.09E-68 | 1.55 | 5.84E-70 | / |
| *Cd302* | 3.05 | 2.80E-03 | 2.73 | 1.16E-93 | 5.64 | 1.70E-196 | Potential multifunctional C-type lectin receptor that may play roles in endocytosis and phagocytosis as well as in cell adhesion and migration. |
| *Cd5l* | 3.58 | 6.73E-04 | 7.54 | 2.41E-29 | 10.04 | 3.75E-58 | Secreted protein that acts as a key regulator of lipid synthesis: mainly expressed by macrophages in lymphoid and inflamed tissues and regulates mechanisms in inflammatory responses and inhibit lipid droplet size in adipocytes. |
| *Cfi* | 3.09 | 4.03E-03 | 6.60 | 6.04E-289 | 2.56 | 5.78E-23 | This gene regulate the immune response by controlling all complement pathways. |
| *Cldn14* | 2.54 | 1.78E-02 | 10.36 | 1.17E-16 | 6.76 | 4.51E-79 | / |
| *Cmbl* | 3.62 | 3.27E-03 | 4.23 | 5.63E-157 | 1.28 | 7.24E-08 | Related to the oxidation pathway of cytochrome P450 and activate medoxomil-ester prodrugs. |
| *Colec11* | 4.56 | 2.03E-04 | 8.71 | 8.84E-36 | 11.18 | 2.01E-291 | plays a role in innate immunity, apoptosis and embryogenesis. |
| *Cox4i1* | 2.92 | 4.46E-03 | 1.88 | 5.24E-66 | 1.03 | 1.78E-25 | Respiratory electron transport, ATP synthesis by chemiosmotic coupling, and heat production by uncoupling proteins. |
| *Cps1* | 4.18 | 1.30E-03 | 11.60 | 2.60E-73 | 9.09 | 1.48E-41 | Involved in the urea cycle of ureotelic animals. |
| *Cpt2* | 1.93 | 4.66E-02 | 1.66 | 2.45E-16 | 1.52 | 5.65E-34 | Participating in fatty acids β oxidation and and PPARA activates gene expression. |
| *Dgat2* | 3.96 | 1.74E-03 | 1.89 | 6.44E-35 | 3.89 | 1.60E-68 | This gene is required for synthesis and storage of intracellular triglycerides. |
| *Dpm3* | 2.48 | 1.23E-02 | 3.23 | 7.99E-33 | 1.58 | 9.62E-33 | / |
| *Esd* | 2.25 | 1.71E-02 | 2.05 | 1.03E-52 | 1.26 | 3.35E-39 | / |
| *Etfa* | 2.13 | 2.53E-02 | 2.52 | 6.28E-121 | 1.21 | 1.12E-31 | Participate in catalyzing the mitochondrial fatty acid beta-oxidation. |
| *Exosc5* | 2.22 | 1.86E-02 | 1.43 | 7.92E-11 | 1.18 | 7.13E-16 | Predicted to enable RNA binding activity and participates in a multitude of cellular RNA processing and degradation events. |
| *F2* | 3.85 | 6.35E-04 | 11.18 | 1.31E-33 | 4.95 | 1.16E-12 | This gene participate in blood homeostasis, inflammation and wound healing. |
| *F7* | 2.44 | 1.25E-02 | 7.49 | 6.64E-51 | 10.30 | 6.44E-129 | blood coagulation. |
| *Fga* | 3.03 | 6.93E-03 | 15.45 | 1.84E-50 | 8.48 | 0.00E+00 | Hemostasis and may facilitate the immune response. |
| *Fgg* | 1.99 | 3.45E-02 | 5.85 | 2.18E-138 | 10.47 | 0.00E+00 | Hemostasis and may facilitate the immune response. |
| *Fgl1* | 5.00 | 7.76E-05 | 2.43 | 1.72E-03 | 10.72 | 0.00E+00 | May play a role in the development of hepatocellular carcinomas. |
| *Fkbp2* | 3.29 | 2.49E-03 | 2.88 | 1.59E-128 | 1.05 | 2.84E-21 | Accelerate the folding of proteins and may also act as a component of membrane cytoskeletal scaffolds. |
| *Gcat* | 3.73 | 7.51E-04 | 4.85 | 1.04E-77 | 2.62 | 4.61E-40 | Catalyzes the cleavage of 2-amino-3-oxobutanoate to glycine and acetyl-CoA. |
| *Gjb2* | 2.61 | 2.15E-02 | 2.13 | 6.02E-08 | 1.02 | 6.51E-03 | Structural component of gap junctions. |
| *Glyctk* | 4.08 | 4.45E-04 | 1.92 | 2.00E-06 | 3.23 | 3.07E-42 | May be involved in serine degradation and fructose metabolism. |
| *Got1* | 4.09 | 3.83E-04 | 1.24 | 8.35E-12 | 2.47 | 5.30E-37 | The aspartate aminotransferase activity is involved in hepatic glucose synthesis during development and in adipocyte glyceroneogenesis. |
| *Gstk1* | 1.93 | 4.14E-02 | 4.19 | 3.30E-99 | 1.57 | 1.25E-08 | catalyzes the conjugation of glutathione to exogenous and endogenous compounds. |
| *Gys2* | 3.45 | 4.79E-03 | 10.87 | 1.91E-19 | 3.03 | 5.18E-21 | Catalytic synthesis of liver glycogen. |
| *Hebp1* | 2.24 | 2.16E-02 | 2.43 | 2.41E-37 | 1.14 | 1.60E-69 | promotes calcium mobilization and chemotaxis in monocytes and dendritic cells. |
| *Hgd* | 2.63 | 6.47E-03 | 6.58 | 4.09E-260 | 1.04 | 4.13E-09 | Involved in the catabolism of the amino acids tyrosine and phenylalanine. |
| *Hibadh* | 2.28 | 3.54E-02 | 1.37 | 7.99E-29 | 1.34 | 5.75E-72 | Play a critical role in the catabolism of L-valine. |
| *Hint1* | 3.81 | 4.38E-04 | 2.65 | 6.89E-182 | 1.26 | 8.49E-19 | This gene encodes a protein that hydrolyzes purine nucleotide phosphoramidates substrates, including AMP-morpholidate, AMP-N-alanine methyl ester, AMP-alpha-acetyl lysine methyl ester, and AMP-NH2. The encoded protein interacts with these substrates via a histidine triad motif. This gene is considered a tumor suppressor gene. |
| *Hint2* | 2.01 | 3.28E-02 | 2.93 | 1.07E-93 | 1.86 | 2.62E-59 | The histidine triad nucleotide‐binding protein 2 (HINT2) is a nucleotide hydrolase and transferase located in mitochondria. HINT2 has multiple functions such as regulating mitochondrial lipid metabolism and respiration and glucose homeostasis. |
| *Hmgcl* | 2.51 | 1.05E-02 | 2.35 | 3.75E-62 | 1.38 | 5.61E-36 | HMGCL (3-Hydroxy-3-Methylglutaryl-CoA Lyase) is a Protein Coding gene. Diseases associated with HMGCL include 3-Hydroxy-3-Methylglutaryl-Coa Lyase Deficiency and Long-Chain 3-Hydroxyacyl-Coa Dehydrogenase Deficiency. Among its related pathways are Ketone body metabolism and Peroxisomal lipid metabolism. |
| *Hp* | 2.04 | 2.74E-02 | 2.29 | 1.72E-02 | 11.06 | 0.00E+00 | HP (Haptoglobin) is a Protein Coding gene. Diseases associated with HP include Anhaptoglobinemia and Plasmodium Falciparum Malaria. Among its related pathways are Innate Immune System and Binding and Uptake of Ligands by Scavenger Receptors. |
| *Igfbp1* | 7.09 | 2.76E-07 | 11.49 | 6.29E-18 | 6.86 | 3.71E-127 | Expression and secretion of the chemokine IGFBP1 mediates the increase in white adipose tissue lipolysis and serum free fatty acids induced by Wilms' tumor 1-associating protein (WTAP) deficiency and leads to hepatocyte degeneration. |
| *Itih2* | 3.26 | 1.77E-03 | 13.09 | 2.24E-27 | 9.07 | 0.00E+00 | The inter-alpha-trypsin inhibitors (ITI) are a family of structurally related plasma serine protease inhibitors involved in extracellular matrix stabilization and in prevention of tumor metastasis. |
| *Itih4* | 3.52 | 7.79E-04 | 9.05 | 6.42E-158 | 10.07 | 0.00E+00 | ITIH4 (Inter-Alpha-Trypsin Inhibitor Heavy Chain 4) is a Protein Coding gene. Diseases associated with ITIH4 include Aids Dementia Complex and Human Immunodeficiency Virus Infectious Disease. Among its related pathways are Response to elevated platelet cytosolic Ca2+. Gene Ontology (GO) annotations related to this gene include serine-type endopeptidase inhibitor activity and endopeptidase inhibitor activity. |
| *Khk* | 1.91 | 4.25E-02 | 6.15 | 7.10E-22 | 6.04 | 3.58E-71 | Genetic deletion of KHK or knockdown with small interfering RNA protects mice from the deleterious metabolic effects of fructose, including obesity, insulin resistance, steatosis, hyperlipidemia, and hepatic inflammation, supporting therapeutic targeting of KHK as a method of mitigating metabolic diseases including MASLD/NASH, T2D, and cardiovascular disease. |
| *Kng1* | 2.73 | 5.42E-03 | 7.63 | 4.34E-30 | 10.34 | 0.00E+00 | KNG1 is an important part of the coagulation system and the kinin–kallikrein system (KKS). KKS activation mediates the production of the inflammatory mediator bradykinin (BK), thereby playing an important role in inflammatory diseases. |
| *Lect2* | 4.35 | 3.36E-04 | 12.26 | 1.10E-32 | 11.13 | 1.05E-133 | LECT2 augments lipid accumulation during 3T3-L1 cell differentiation by activating SREBP1c-mediated signaling and stimulates inflammation and insulin resistance in adipocytes via activation of a CD209/P38-dependent pathway. |
| *Mettl7b* | 2.37 | 1.71E-02 | 15.09 | 4.77E-36 | 1.85 | 1.82E-20 | / |
| *Mrpl2* | 4.53 | 5.51E-04 | 2.29 | 2.67E-21 | 1.13 | 3.96E-38 | MRPL2 (Mitochondrial Ribosomal Protein L2) is a Protein Coding gene. Among its related pathways are Mitochondrial translation and Metabolism of proteins. Gene Ontology (GO) annotations related to this gene include RNA binding and structural constituent of ribosome. |
| *Mrpl41* | 2.96 | 3.32E-03 | 2.20 | 3.18E-29 | 1.13 | 7.83E-35 | MRPL41 (Mitochondrial Ribosomal Protein L41) is a Protein Coding gene. Among its related pathways are Mitochondrial translation and Metabolism of proteins. |
| *Mrpl54* | 2.85 | 7.62E-03 | 3.09 | 7.70E-150 | 1.25 | 1.84E-28 | MRPL54 (Mitochondrial Ribosomal Protein L54) is a Protein Coding gene. Among its related pathways are Mitochondrial translation and Metabolism of proteins. |
| *Mst1* | 4.14 | 7.98E-04 | 1.75 | 5.94E-25 | 4.28 | 0.00E+00 | Downregulation of hepatic MST1 expression is involved in the regulation of hepatic fat synthesis and catabolism. |
| *Mttp* | 2.83 | 1.64E-02 | 10.79 | 5.76E-25 | 8.22 | 9.54E-41 | Microsomal triglyceride transfer protein (MTTP) is highly expressed in adipose tissue and is involved in regulating lipid metabolism by facilitating triglyceride transport between membrane vesicles. VLDL particles are formed in the endoplasmic reticulum, where apolipoprotein (apo)B100 is lipidated in a process catalyzed by the enzyme microsomal triglyceride transfer protein (MTTP). |
| *Ndufa11* | 3.40 | 8.45E-04 | 3.57 | 6.32E-148 | 1.05 | 4.11E-19 | NDUFA11 (NADH: Ubiquinone Oxidoreductase Subunit A11) is a Protein Coding gene. Diseases associated with NDUFA11 include Mitochondrial Complex I Deficiency, Nuclear Type 14 and Isolated Complex I Deficiency. Among its related pathways are Respiratory electron transport, ATP synthesis by chemiosmotic coupling, and heat production by uncoupling proteins. |
| *Ndufa2* | 2.47 | 1.04E-02 | 2.98 | 4.74E-116 | 1.18 | 7.29E-27 | NDUFA2 (NADH: Ubiquinone Oxidoreductase Subunit A2) is a Protein Coding gene. Diseases associated with NDUFA2 include Mitochondrial Complex I Deficiency, Nuclear Type 13 and Leukoencephalopathy, Cystic, Without Megalencephaly. Among its related pathways are Respiratory electron transport, ATP synthesis by chemiosmotic coupling, and heat production by uncoupling proteins. and Complex I biogenesis. |
| *Ndufa3* | 2.32 | 1.80E-02 | 3.17 | 1.60E-13 | 1.02 | 1.24E-13 | NDUFA3 (NADH: Ubiquinone Oxidoreductase Subunit A3) is a Protein Coding gene. Diseases associated with NDUFA3 include Schizophrenia 5 and Retinitis Pigmentosa. Among its related pathways are Respiratory electron transport, ATP synthesis by chemiosmotic coupling, and heat production by uncoupling proteins. and Complex I biogenesis. |
| *Ndufa4* | 2.61 | 7.14E-03 | 2.75 | 5.97E-130 | 1.57 | 1.37E-21 | NDUFA4 (NDUFA4 Mitochondrial Complex Associated) is a Protein Coding gene. Diseases associated with NDUFA4 include Mitochondrial Complex Iv Deficiency, Nuclear Type 21 and Lactic Acidosis. Among its related pathways are Respiratory electron transport, ATP synthesis by chemiosmotic coupling, and heat production by uncoupling proteins. and Gene expression (Transcription). |
| *Ndufaf2* | 4.81 | 3.50E-04 | 2.02 | 1.04E-05 | 1.48 | 1.57E-12 | NDUFAF2 (NADH:Ubiquinone Oxidoreductase Complex Assembly Factor 2) is a Protein Coding gene. Diseases associated with NDUFAF2 include Mitochondrial Complex I Deficiency, Nuclear Type 10 and Mitochondrial Complex I Deficiency, Nuclear Type 1. Among its related pathways are Respiratory electron transport, ATP synthesis by chemiosmotic coupling, and heat production by uncoupling proteins. and Complex I biogenesis. |
| *Ndufb10* | 2.63 | 7.59E-03 | 2.40 | 2.33E-41 | 1.14 | 6.15E-42 | NDUFB10 (NADH:Ubiquinone Oxidoreductase Subunit B10) is a Protein Coding gene. Diseases associated with NDUFB10 include Mitochondrial Complex I Deficiency, Nuclear Type 35 and Isolated Complex I Deficiency. Among its related pathways are Respiratory electron transport, ATP synthesis by chemiosmotic coupling, and heat production by uncoupling proteins. and Complex I biogenesis. |
| *Ndufb6* | 3.16 | 2.41E-03 | 3.33 | 6.98E-154 | 1.41 | 2.45E-34 | NDUFB6 expression can participate in the regulation of hepatic lipid transport and ATP synthesis. |
| *Ninj1* | 2.33 | 1.56E-02 | 2.18 | 4.62E-86 | 2.32 | 4.11E-76 | Nerve injury–induced protein 1 (Ninjurin-1 [Ninj1]) plays an important role in various inflammatory processes by regulating leukocyte infiltration. |
| *Nit2* | 1.79 | 4.78E-02 | 3.74 | 1.82E-207 | 1.41 | 6.74E-63 | NIT2 (Nitrilase Family Member 2) is a Protein Coding gene. Diseases associated with NIT2 include Histidinemia and Atrial Septal Defect 4. Among its related pathways are Innate Immune System. |
| *Nnmt* | 3.62 | 7.09E-04 | 3.09 | 1.04E-25 | 8.50 | 1.16E-86 | Nicotinamide [NAM] causes hepatotoxicity and triglyceride accumulation in the liver. Nicotinamide N-methyltransferase (NNMT) is the major NAM metabolizing enzyme, and NNMT metabolically regulates the energy expenditure of white adipose tissue as well as hepatic gluconeogenesis and cholesterol biosynthesis, and promotes hepatic steatosis and fibrosis. |
| *Nudt6* | 2.97 | 4.63E-03 | 2.42 | 1.44E-09 | 2.41 | 9.03E-43 | NUDT6 (Nudix Hydrolase 6) is a Protein Coding gene. Diseases associated with NUDT6 include Desmoid Disease, Hereditary and Gingival Overgrowth. Among its related pathways are Apoptotic Pathways in Synovial Fibroblasts and GPCR Pathway. |
| *Orm1* | 2.36 | 1.59E-02 | 11.37 | 5.53E-118 | 10.97 | 1.20E-254 | The ORM1 gene encodes a key acute-phase plasma protein involved in the immune response and is implicated in sphingolipid, cholesterol, and triglyceride synthesis. |
| *Pebp1* | 2.76 | 7.00E-03 | 2.05 | 8.62E-71 | 3.09 | 3.37E-276 | PEBP1 is a member of the phosphatidylethanolamine-binding protein family and has been shown to regulate a variety of signaling pathways, including the MAP kinase (MAPK), NF-kappa B, and glycogen synthase kinase-3 (GSK-3) signaling pathways, which have been implicated in the immune response and inflammatory response. |
| *Phyhd1* | 2.68 | 7.41E-03 | 2.40 | 6.89E-37 | 4.07 | 4.29E-67 | It is linked to carbohydrate metabolism that catalyzes the conversion of 2-oxoglutarate to succinate and CO(2) in an iron-dependent manner |
| *Ppib* | 2.43 | 1.26E-02 | 1.72 | 9.56E-62 | 1.16 | 2.72E-32 | It is involved in the synthesis of biological vesicles and protein folding and affects the expressions of proteins related to apoptosis, migration, and invasion. |
| *Prdx4* | 4.05 | 1.08E-03 | 3.30 | 9.88E-165 | 1.45 | 2.73E-21 | It plays a role in cell protection against oxidative stress as a sensor of hydrogen peroxide-mediated signaling events. It regulates the activation of NF-kappa-B in the cytosol by a modulation of I-kappa-B-alpha phosphorylation as well. |
| *Proc* | 2.08 | 3.07E-02 | 8.40 | 1.87E-20 | 6.79 | 6.25E-85 | It exerts a protective effect on the endothelial cell barrier function. |
| *Psmg4* | 3.00 | 5.17E-03 | 1.72 | 3.37E-11 | 1.13 | 4.99E-19 | There are positive correlations between PSMG family genes and the immune response, metabolism of ubiquinone, cell cycle regulatory pathways. |
| *Pzp* | 2.44 | 1.15E-02 | 11.36 | 7.12E-25 | 10.83 | 2.36E-21 | It activates brown adipose tissue and is responsible for all metabolic regulatory functions. |
| *Rarres1* | 2.69 | 3.66E-02 | 4.14 | 8.90E-102 | 1.73 | 2.13E-06 | It regulates lipid accumulation. |
| *Rbp4* | 2.38 | 1.45E-02 | 10.11 | 7.62E-239 | 7.72 | 9.75E-70 | It delivers retinol from the liver stores to the peripheral tissues probably. |
| *Rbp7* | 3.79 | 8.35E-04 | 2.80 | 4.84E-02 | 7.74 | 7.98E-128 | It promotes adipogenesis in vitro and regulates expression of genes involved in retinol metabolism. |
| *Rcan1* | 4.20 | 2.34E-02 | 2.05 | 5.16E-17 | 2.37 | 7.41E-70 | There is a high correlation between Rcan1 expression and metabolic syndrome by suppressing non-shivering thermogenesis. |
| *Reep6* | 2.43 | 1.19E-02 | 2.61 | 1.80E-03 | 2.85 | 4.44E-79 | Genetic inactivation of REEP6 reduces energy expenditure, increases adiposity, and the susceptibility to obesity-related metabolic dysfunction. |
| *Rnaseh2c* | 2.10 | 2.75E-02 | 1.81 | 1.12E-08 | 1.44 | 9.11E-40 | Defective RNASEH2 may induce accumulation of self-nucleic acid species that trigger chronic type I interferon and inflammatory responses, leading to The neuroinflammatory autoimmune disease. |
| *Sdc2* | 5.15 | 1.16E-03 | 1.70 | 2.44E-14 | 9.25 | 0.00E+00 | This gene is associated with inflammation. |
| *Sdsl* | 6.18 | 2.20E-06 | 3.48 | 1.18E-15 | 2.24 | 3.33E-35 | This gene is associated with inflammation. |
| *Sec11c* | 2.15 | 2.37E-02 | 2.32 | 1.38E-37 | 2.48 | 9.12E-42 | It is atalytic component of the signal peptidase complex (SPC) which catalyzes the cleavage of N-terminal signal sequences from nascent proteins as they are translocated into the lumen of the endoplasmic reticulum. |
| *Serpinf2* | 2.20 | 3.09E-02 | 6.81 | 2.19E-24 | 7.70 | 7.37E-217 | It is a serum protein selectively expressed and secreted by the liver are differentially expressed in patients with different degrees of MASLD. |
| *Slc17a4* | 6.19 | 2.90E-06 | 9.12 | 1.03E-09 | 1.25 | 3.36E-03 | Proteoliposomal systems induced the accumulation of uric acid. |
| *Slc22a1* | 1.94 | 4.19E-02 | 7.58 | 1.51E-114 | 9.00 | 0.00E+00 | Encoded a hepatic plasma membrane transporter ,Involved in hepatic uptake of vitamin B1/thiamine, regulated hepatic lipid and energy metabolism. |
| *Slc25a20* | 2.29 | 2.20E-02 | 1.65 | 3.58E-23 | 1.79 | 2.39E-20 | Involved in fatty acid oxidation. |
| *Slc25a25* | 2.32 | 2.04E-02 | 1.53 | 1.90E-06 | 1.53 | 8.80E-20 | Involved in metabolism, abnormal expression leads to reduced metabolic efficiency. |
| *Slc25a47* | 1.84 | 4.63E-02 | 9.32 | 1.25E-106 | 10.49 | 3.22E-297 | regulated lipid homeostasis ,hepatocyte-specific mitochondrial NAD+ transporter. |
| *Slc40a1* | 2.39 | 1.56E-02 | 2.34 | 5.07E-13 | 1.11 | 9.03E-05 | The only discovered iron export protein in mammals, increased expression occurs with iron deposition. |
| *Soat2* | 3.52 | 1.10E-03 | 4.12 | 1.56E-06 | 2.01 | 6.69E-06 | Catalyzes the formation of fatty acid-cholesterol esters, plays a role in lipoprotein assembly and dietary cholesterol absorption, and provides cholesterol esters for lipoprotein secretion by hepatocytes and intestinal mucosa. |
| *Spp2* | 2.56 | 7.13E-03 | 10.70 | 8.24E-19 | 11.76 | 6.31E-120 | Secretory factors that negatively regulate liver regeneration. |
| *Stab2* | 3.30 | 6.05E-03 | 4.39 | 4.88E-04 | 9.69 | 2.48E-51 | Clearance of transforming growth factor, beta-induced, and Periostin in LSEC. |
| *Stard5* | 2.98 | 1.12E-02 | 1.51 | 7.44E-08 | 4.06 | 3.01E-44 | Involved in intracellular transport of lipids, synthesis of bile acids and bile salts, and metabolism. |
| *Tat* | 3.11 | 5.53E-03 | 11.55 | 8.14E-62 | 9.32 | 0.00E+00 | Encodes a mitochondrial protein tyrosine aminotransferase which is present in the liver and catalyzes the conversion of L-tyrosine into p-hydroxyphenylpyruvate. |
| *Tdo2* | 2.32 | 1.58E-02 | 15.15 | 2.41E-37 | 10.83 | 9.12E-251 | Involved in tryptophan utilization and NAD metabolism,and can be upregulated by steroids. |
| *Timm9* | 2.04 | 4.11E-02 | 1.86 | 5.25E-11 | 1.57 | 1.38E-32 | Involved in peroxisomal lipid metabolism. |
| *Tmem176a* | 2.68 | 8.80E-03 | 3.33 | 3.59E-65 | 1.40 | 1.68E-08 | Inhibited ERK signaling. |
| *Tmem82* | 2.43 | 1.14E-02 | 1.09 | 1.16E-03 | 2.64 | 7.30E-13 | / |
| *Tst* | 3.69 | 1.27E-03 | 3.37 | 6.86E-49 | 2.01 | 1.05E-19 | Involved in sulfur amino acid metabolism and metabolism. |
| *Ttc32* | 2.51 | 9.27E-03 | 2.15 | 2.19E-12 | 1.07 | 1.39E-05 | / |
| *Ugp2* | 3.18 | 7.26E-03 | 1.34 | 1.12E-27 | 1.19 | 2.58E-17 | involved in the process of glycogenesis in liver. |

**Table S6. Peak normalized read counts of 121 uniform genes across all histone samples.**

| Uniform Gene | Gene_chr | Gene_stat | Gene_end | Gene_strand | Peak_ID | Peak_chr | Peak_stat | Peak_end | NL1_H3K4me3 | MASLD1_H3K4me3 | MASLD3_H3K4me3 | NL1_h3k27ac | MASLD1_h3k27ac | MASLD3_h3k27ac | NL1_H3K4me1 | MASLD1_  H3K4me1 | MASLD3_  H3K4me1 |
| --- | --- | --- | --- | --- | --- | --- | --- | --- | --- | --- | --- | --- | --- | --- | --- | --- | --- |
| *Abat* | 16 | 8674596 | 8784575 | + | 16:8737566-8744017 | 16 | 8737566 | 8744017 | 13.32 | 10.61 | 4.42 | 36.50 | 160.24 | 89.38 | 43.48 | 64.81 | 44.12 |
| *Abcg5* | 2 | 43812472 | 43838865 | - | 2:43815327-43815458 | 2 | 43815327 | 43815458 | 0.00 | 0.00 | 0.00 | 0.00 | 117.69 | 30.03 | 49.23 | 21.28 | 51.22 |
| *Acaa2* | 18 | 49782164 | 49813953 | - | 18:49783784-49792122 | 18 | 49783784 | 49792122 | 6.67 | 15.15 | 8.32 | 22.39 | 95.61 | 80.71 | 34.29 | 105.24 | 84.75 |
| *Acadl* | 2 | 210187126 | 210225447 | - | 2:210188270-210191972 | 2 | 210188270 | 210191972 | 13.81 | 13.47 | 15.32 | 23.69 | 71.80 | 127.48 | 34.16 | 55.50 | 47.65 |
| *Acadm* | 1 | 75724431 | 75787575 | + | 1:75723350-75729355 | 1 | 75723350 | 75729355 | 101.28 | 125.50 | 98.21 | 38.66 | 140.06 | 102.91 | 50.37 | 79.02 | 71.30 |
| *Acot13* | 6 | 24667035 | 24705065 | + | 6:24708789-24723163 | 6 | 24708789 | 24723163 | 54.73 | 83.45 | 81.18 | 37.68 | 126.96 | 109.29 | 39.31 | 89.25 | 82.29 |
| *Acox2* | 3 | 58505136 | 58537283 | - | 3:58515482-58541104 | 3 | 58515482 | 58541104 | 16.25 | 16.04 | 11.08 | 22.81 | 102.35 | 40.12 | 61.83 | 85.29 | 63.83 |
| *Acsm3* | 16 | 20610243 | 20797581 | + | 16:20594583-20601898 | 16 | 20594583 | 20601898 | 8.95 | 9.50 | 11.90 | 24.66 | 112.40 | 63.84 | 52.02 | 69.56 | 56.29 |
| *Afm* | 4 | 73481745 | 73504001 | + | 4:73478641-73490391 | 4 | 73478641 | 73490391 | 36.10 | 44.52 | 35.04 | 30.34 | 88.96 | 50.39 | 39.72 | 67.90 | 63.81 |
| *Agt* | 1 | 230690776 | 230745576 | - | 1:230723219-230732200 | 1 | 230723219 | 230732200 | 11.99 | 17.21 | 15.91 | 10.75 | 105.34 | 44.94 | 77.89 | 85.51 | 74.55 |
| *Agxt* | 2 | 240868824 | 240880502 | + | 2:240854575-240877515 | 2 | 240854575 | 240877515 | 34.58 | 50.65 | 25.91 | 10.50 | 55.66 | 24.90 | 59.51 | 64.09 | 45.26 |
| *Ambp* | 9 | 114060127 | 114078328 | - | 9:114071756-114083598 | 9 | 114071756 | 114083598 | 60.71 | 67.07 | 47.48 | 21.97 | 103.24 | 42.86 | 38.10 | 57.38 | 41.74 |
| *Angptl6* | 19 | 10092338 | 10102678 | - | 19:10101288-10107691 | 19 | 10101288 | 10107691 | 25.93 | 55.51 | 46.67 | 19.48 | 107.26 | 86.97 | 22.34 | 47.29 | 41.27 |
| *Apoa2* | 1 | 161222292 | 161223631 | - | 1:161213282-161230170 | 1 | 161213282 | 161230170 | 47.26 | 71.53 | 50.16 | 53.64 | 133.50 | 83.98 | 34.25 | 54.36 | 44.80 |
| *Apoa4* | 11 | 116820700 | 116823304 | - | 11:116812504-116817103 | 11 | 116812504 | 116817103 | 18.50 | 20.83 | 20.59 | 17.57 | 122.44 | 22.12 | 55.84 | 70.21 | 44.39 |
| *Apoa5* | 11 | 116789367 | 116792420 | - | 11:116785679-116794690 | 11 | 116785679 | 116794690 | 70.30 | 114.49 | 81.89 | 20.92 | 88.74 | 32.31 | 34.19 | 61.79 | 45.03 |
| *Apoc2* | 19 | 44946035 | 44949565 | + | 19:44901794-44959651 | 19 | 44901794 | 44959651 | 38.59 | 56.52 | 44.22 | 29.93 | 117.47 | 43.15 | 41.06 | 48.15 | 36.51 |
| *Apoc3* | 11 | 116829706 | 116833072 | + | 11:116825411-116844767 | 11 | 116825411 | 116844767 | 46.18 | 60.38 | 37.66 | 17.82 | 116.69 | 21.05 | 45.21 | 45.19 | 36.01 |
| *Apoc4* | 19 | 44942237 | 44945496 | + | 19:44901794-44959651 | 19 | 44901794 | 44959651 | 38.59 | 56.52 | 44.22 | 29.93 | 117.47 | 43.15 | 41.06 | 48.15 | 36.51 |
| *Apoh* | 17 | 66212033 | 66256525 | - | 17:66216903-66237302 | 17 | 66216903 | 66237302 | 37.37 | 40.57 | 31.36 | 28.38 | 99.32 | 66.32 | 33.25 | 58.12 | 49.67 |
| *Aqp9* | 15 | 58138169 | 58185911 | + | 15:58136953-58149543 | 15 | 58136953 | 58149543 | 51.54 | 56.38 | 36.92 | 66.82 | 152.24 | 103.49 | 50.65 | 71.37 | 71.81 |
| *Asgr1* | 17 | 7173431 | 7179564 | - | 17:7183262-7184567 | 17 | 7183262 | 7184567 | 2.51 | 12.67 | 18.50 | 12.70 | 82.16 | 30.75 | 33.24 | 61.74 | 27.12 |
| *Aspg* | 14 | 104085686 | 104115582 | + | 14:104085830-104093410 | 14 | 104085830 | 104093410 | 22.88 | 45.98 | 31.03 | 9.94 | 40.35 | 13.33 | 45.16 | 52.76 | 34.00 |
| *Atf5* | 19 | 49928702 | 49933935 | + | 19:49921606-49932275 | 19 | 49921606 | 49932275 | 60.02 | 78.47 | 60.75 | 26.92 | 75.42 | 53.05 | 40.33 | 45.42 | 40.81 |
| *Atox1* | 5 | 151742316 | 151772532 | - | 5:151743703-151760331 | 5 | 151743703 | 151760331 | 19.68 | 35.72 | 28.13 | 24.92 | 77.58 | 70.49 | 43.37 | 83.23 | 73.39 |
| *Baat* | 9 | 101354182 | 101385400 | - | 9:101364097-101389544 | 9 | 101364097 | 101389544 | 28.13 | 33.90 | 34.27 | 29.04 | 99.71 | 70.16 | 43.22 | 60.58 | 73.01 |
| *Blvrb* | 19 | 40447765 | 40465764 | - | 19:40440575-40445410 | 19 | 40440575 | 40445410 | 85.95 | 141.12 | 97.92 | 30.77 | 105.82 | 112.81 | 26.14 | 49.90 | 35.40 |
| *C1qa* | 1 | 22635077 | 22639678 | + | 1:22644834-22645153 | 1 | 22644834 | 22645153 | 6.34 | 87.62 | 18.94 | 8.18 | 43.53 | 35.76 | 0.00 | 15.76 | 11.20 |
| *Ccs* | 11 | 66593153 | 66606019 | + | 11:66590014-66608579 | 11 | 66590014 | 66608579 | 17.83 | 34.37 | 19.17 | 19.37 | 82.42 | 30.63 | 51.72 | 74.41 | 52.24 |
| *Cd302* | 2 | 159768628 | 159798255 | - | 2:159781679-159803400 | 2 | 159781679 | 159803400 | 20.17 | 27.56 | 22.94 | 23.29 | 71.30 | 56.14 | 61.32 | 87.89 | 90.22 |
| *Cd5l* | 1 | 157830911 | 157898256 | - | 1:157843295-157843888 | 1 | 157843295 | 157843888 | 21.04 | 47.68 | 20.00 | 12.12 | 63.79 | 41.03 | 14.72 | 32.58 | 28.12 |
| *Cfi* | 4 | 109731008 | 109802150 | - | 4:109796766-109804469 | 4 | 109796766 | 109804469 | 51.61 | 45.84 | 48.57 | 31.92 | 101.70 | 109.86 | 41.00 | 69.05 | 71.07 |
| *Cldn14* | 21 | 36460621 | 36576569 | - | 21:36498184-36502982 | 21 | 36498184 | 36502982 | 19.32 | 34.56 | 12.79 | 17.08 | 107.88 | 50.33 | 36.97 | 65.05 | 65.20 |
| *Cmbl* | 5 | 10275875 | 10307902 | - | 5:10294998-10312227 | 5 | 10294998 | 10312227 | 22.41 | 39.90 | 25.67 | 27.84 | 123.13 | 52.24 | 47.85 | 67.71 | 50.49 |
| *Colec11* | 2 | 3594832 | 3644644 | + | 2:3582799-3588633 | 2 | 3582799 | 3588633 | 10.07 | 14.34 | 18.18 | 33.92 | 102.28 | 89.18 | 27.44 | 54.04 | 45.80 |
| *Cox4i1* | 16 | 85798633 | 85807068 | + | 16:85796897-85802715 | 16 | 85796897 | 85802715 | 89.31 | 130.63 | 114.23 | 17.60 | 52.61 | 35.53 | 35.40 | 71.13 | 59.00 |
| *Cps1* | 2 | 210477682 | 210679107 | + | 2:210536554-210579722 | 2 | 210536554 | 210579722 | 31.33 | 32.50 | 33.59 | 22.04 | 124.80 | 56.41 | 51.83 | 67.32 | 72.44 |
| *Cpt2* | 1 | 53196792 | 53214197 | + | 1:53218366-53221680 | 1 | 53218366 | 53221680 | 52.90 | 60.46 | 71.09 | 25.32 | 113.34 | 121.12 | 35.03 | 60.17 | 39.47 |
| *Dgat2* | 11 | 75759512 | 75801535 | + | 11:75766493-75797223 | 11 | 75766493 | 75797223 | 16.38 | 28.05 | 16.13 | 19.39 | 47.55 | 21.07 | 59.22 | 74.05 | 55.49 |
| *Dpm3* | 1 | 155139891 | 155140595 | - | 1:155122394-155140945 | 1 | 155122394 | 155140945 | 71.09 | 125.17 | 86.10 | 41.38 | 139.84 | 96.21 | 38.00 | 69.72 | 37.39 |
| *Esd* | 13 | 46771256 | 46797420 | - | 13:46792025-46802973 | 13 | 46792025 | 46802973 | 33.10 | 60.04 | 42.17 | 21.14 | 87.57 | 60.53 | 54.93 | 104.88 | 93.56 |
| *Etfa* | 15 | 76188555 | 76311730 | - | 15:76305078-76314546 | 15 | 76305078 | 76314546 | 42.23 | 58.68 | 49.67 | 23.91 | 59.84 | 56.02 | 52.78 | 95.68 | 80.96 |
| *Exosc5* | 19 | 41386371 | 41397362 | - | 19:41393559-41403627 | 19 | 41393559 | 41403627 | 54.87 | 76.24 | 79.64 | 17.63 | 56.40 | 41.91 | 33.58 | 57.12 | 50.71 |
| *F2* | 11 | 46719196 | 46739506 | + | 11:46746667-46746857 | 11 | 46746667 | 46746857 | 5.23 | 0.00 | 0.00 | 14.41 | 112.13 | 63.15 | 21.78 | 10.40 | 0.00 |
| *F7* | 13 | 113105788 | 113120685 | + | 13:113105309-113106468 | 13 | 113105309 | 113106468 | 57.55 | 96.71 | 33.32 | 15.03 | 84.10 | 27.49 | 13.34 | 31.17 | 15.98 |
| *Fga* | 4 | 154583128 | 154590745 | - | 4:154582641-154624759 | 4 | 154582641 | 154624759 | 50.32 | 41.71 | 37.35 | 25.22 | 145.87 | 83.58 | 42.04 | 57.09 | 42.59 |
| *Fgg* | 4 | 154604134 | 154612967 | - | 4:154582641-154624759 | 4 | 154582641 | 154624759 | 50.32 | 41.71 | 37.35 | 25.22 | 145.87 | 83.58 | 42.04 | 57.09 | 42.59 |
| *Fgl1* | 8 | 17864380 | 17910365 | - | 8:17890025-17900437 | 8 | 17890025 | 17900437 | 43.87 | 38.85 | 50.29 | 29.89 | 181.18 | 148.31 | 32.90 | 41.69 | 43.29 |
| *Fkbp2* | 11 | 64241003 | 64244132 | + | 11:64224962-64249347 | 11 | 64224962 | 64249347 | 48.03 | 91.78 | 65.48 | 24.76 | 65.02 | 54.13 | 35.23 | 53.02 | 33.52 |
| *Gcat* | 22 | 37807905 | 37817176 | + | 22:37801839-37810016 | 22 | 37801839 | 37810016 | 72.63 | 135.69 | 88.38 | 17.07 | 60.49 | 44.91 | 43.52 | 62.12 | 43.76 |
| *Gjb2* | 13 | 20187463 | 20192938 | - | 13:20196993-20200376 | 13 | 20196993 | 20200376 | 11.40 | 18.43 | 20.10 | 8.80 | 37.54 | 38.04 | 67.80 | 107.30 | 87.30 |
| *Glyctk* | 3 | 52287089 | 52295257 | + | 3:52264289-52296816 | 3 | 52264289 | 52296816 | 41.81 | 65.52 | 54.84 | 28.52 | 108.21 | 83.80 | 49.35 | 89.44 | 76.58 |
| *Got1* | 10 | 99396870 | 99430624 | - | 10:99407532-99437186 | 10 | 99407532 | 99437186 | 33.24 | 38.88 | 51.29 | 18.56 | 74.26 | 49.99 | 52.20 | 76.83 | 65.73 |
| *Gstk1* | 7 | 143244093 | 143270854 | + | 7:143278817-143280991 | 7 | 143278817 | 143280991 | 4.52 | 16.60 | 15.48 | 24.91 | 125.21 | 145.72 | 20.49 | 77.65 | 69.07 |
| *Gys2* | 12 | 21536107 | 21604847 | - | 12:21559086-21571719 | 12 | 21559086 | 21571719 | 3.61 | 7.35 | 10.33 | 23.63 | 117.02 | 50.68 | 54.16 | 63.33 | 70.26 |
| *Hebp1* | 12 | 12974870 | 13000265 | - | 12:12989895-13008215 | 12 | 12989895 | 13008215 | 29.60 | 51.20 | 38.35 | 19.99 | 60.03 | 54.49 | 52.60 | 100.94 | 79.17 |
| *Hgd* | 3 | 120628172 | 120682269 | - | 3:120675527-120686304 | 3 | 120675527 | 120686304 | 84.49 | 80.87 | 80.09 | 28.05 | 236.77 | 105.30 | 41.62 | 58.66 | 49.06 |
| *Hibadh* | 7 | 27525442 | 27662883 | - | 7:27655855-27664859 | 7 | 27655855 | 27664859 | 32.97 | 47.22 | 44.32 | 33.63 | 87.46 | 72.56 | 49.27 | 93.24 | 69.77 |
| *Hint1* | 5 | 131155383 | 131224468 | - | 5:131159207-131166279 | 5 | 131159207 | 131166279 | 53.98 | 75.84 | 54.87 | 33.92 | 146.98 | 126.41 | 35.12 | 73.40 | 71.45 |
| *Hint2* | 9 | 35812960 | 35815354 | - | 9:35810815-35817218 | 9 | 35810815 | 35817218 | 42.67 | 75.58 | 64.95 | 36.03 | 148.84 | 93.71 | 49.65 | 80.42 | 65.62 |
| *Hmgcl* | 1 | 23801885 | 23838620 | - | 1:23820807-23827267 | 1 | 23820807 | 23827267 | 48.75 | 74.00 | 63.39 | 31.55 | 104.06 | 69.06 | 31.00 | 57.04 | 46.30 |
| *Hp* | 16 | 72054505 | 72061055 | + | 16:72038566-72056476 | 16 | 72038566 | 72056476 | 26.81 | 29.51 | 16.26 | 26.96 | 222.18 | 108.90 | 45.26 | 48.52 | 36.75 |
| *Igfbp1* | 7 | 45888360 | 45893660 | + | 7:45879625-45892018 | 7 | 45879625 | 45892018 | 33.13 | 34.08 | 28.20 | 32.01 | 158.41 | 67.45 | 45.70 | 47.43 | 44.54 |
| *Itih2* | 10 | 7703316 | 7749520 | + | 10:7694728-7712460 | 10 | 7694728 | 7712460 | 42.25 | 51.86 | 41.29 | 23.43 | 110.51 | 63.41 | 47.63 | 79.09 | 67.39 |
| *Itih4* | 3 | 52812962 | 52830688 | - | 3:52824368-52847340 | 3 | 52824368 | 52847340 | 27.68 | 29.78 | 25.54 | 17.80 | 85.78 | 29.76 | 43.15 | 49.87 | 40.64 |
| *Khk* | 2 | 27086747 | 27100762 | + | 2:27085790-27098284 | 2 | 27085790 | 27098284 | 61.87 | 86.40 | 53.76 | 14.77 | 111.97 | 36.34 | 57.69 | 53.87 | 44.92 |
| *Kng1* | 3 | 186717348 | 186744410 | + | 3:186715464-186724731 | 3 | 186715464 | 186724731 | 61.32 | 70.28 | 44.43 | 30.33 | 97.67 | 57.86 | 28.53 | 49.25 | 41.00 |
| *Lect2* | 5 | 135922279 | 135954983 | - | 5:135948856-135956403 | 5 | 135948856 | 135956403 | 38.28 | 42.57 | 53.64 | 35.72 | 190.95 | 70.62 | 38.08 | 50.08 | 52.47 |
| *Ly75* | 2 | 159771851 | 159904710 | - | 2:159781679-159803400 | 2 | 159781679 | 159803400 | 20.17 | 27.56 | 22.94 | 23.29 | 71.30 | 56.14 | 61.32 | 87.89 | 90.22 |
| *Mettl7b* | 12 | 55681736 | 55684611 | + | 12:55679023-55684821 | 12 | 55679023 | 55684821 | 58.10 | 87.15 | 59.04 | 18.21 | 51.75 | 34.66 | 24.67 | 35.05 | 37.41 |
| *Mrpl2* | 6 | 43054029 | 43059438 | - | 6:43057666-43062247 | 6 | 43057666 | 43062247 | 92.46 | 144.60 | 113.42 | 38.19 | 120.00 | 73.78 | 40.91 | 67.73 | 47.47 |
| *Mrpl41* | 9 | 137551879 | 137552555 | + | 9:137559922-137561981 | 9 | 137559922 | 137561981 | 7.92 | 5.58 | 20.00 | 2.30 | 40.80 | 23.86 | 34.41 | 60.94 | 49.13 |
| *Mrpl54* | 19 | 3762682 | 3768575 | + | 19:3757750-3764120 | 19 | 3757750 | 3764120 | 43.36 | 91.05 | 81.93 | 36.56 | 103.10 | 86.30 | 29.47 | 65.13 | 38.16 |
| *Mst1* | 3 | 49683947 | 49689501 | - | 3:49686348-49695723 | 3 | 49686348 | 49695723 | 63.28 | 103.89 | 63.00 | 15.70 | 62.63 | 31.97 | 36.36 | 56.41 | 42.78 |
| *Mttp* | 4 | 99564081 | 99623997 | + | 4:99561695-99565283 | 4 | 99561695 | 99565283 | 86.07 | 138.85 | 114.12 | 47.33 | 192.38 | 122.51 | 33.98 | 41.90 | 32.50 |
| *Ndufa11* | 19 | 5891276 | 5904006 | - | 19:5901095-5907523 | 19 | 5901095 | 5907523 | 59.32 | 104.60 | 79.84 | 15.28 | 49.69 | 34.37 | 35.32 | 52.43 | 38.42 |
| *Ndufa2* | 5 | 140638740 | 140647771 | - | 5:140628974-140651175 | 5 | 140628974 | 140651175 | 52.45 | 84.09 | 67.25 | 31.71 | 96.43 | 68.56 | 44.03 | 63.62 | 53.59 |
| *Ndufa3* | 19 | 54102728 | 54109257 | + | 19:54099381-54105426 | 19 | 54099381 | 54105426 | 72.27 | 94.16 | 94.86 | 20.23 | 45.39 | 33.86 | 22.98 | 38.53 | 28.14 |
| *Ndufa4* | 7 | 10931943 | 10940153 | - | 7:10933316-10941890 | 7 | 10933316 | 10941890 | 65.08 | 88.76 | 79.26 | 30.74 | 123.66 | 98.20 | 31.39 | 67.81 | 70.93 |
| *Ndufaf2* | 5 | 60945177 | 61154531 | + | 5:61157928-61164870 | 5 | 61157928 | 61164870 | 61.01 | 85.43 | 76.74 | 17.90 | 65.56 | 63.13 | 34.82 | 70.95 | 74.24 |
| *Ndufb10* | 16 | 1959538 | 1961975 | + | 16:1963214-1966618 | 16 | 1963214 | 1966618 | 82.84 | 165.95 | 142.65 | 32.08 | 70.80 | 81.64 | 29.12 | 40.54 | 27.39 |
| *Ndufb6* | 9 | 32553001 | 32573184 | - | 9:32570461-32574042 | 9 | 32570461 | 32574042 | 91.07 | 130.34 | 108.66 | 32.72 | 126.62 | 116.32 | 52.42 | 113.49 | 84.56 |
| *Ninj1* | 9 | 93121496 | 93134251 | - | 9:93126847-93136407 | 9 | 93126847 | 93136407 | 27.07 | 65.67 | 42.36 | 42.25 | 62.11 | 50.42 | 44.43 | 54.77 | 44.17 |
| *Nit2* | 3 | 100334739 | 100361635 | + | 3:100322624-100326382 | 3 | 100322624 | 100326382 | 6.97 | 9.60 | 5.93 | 30.45 | 113.72 | 57.94 | 49.34 | 97.91 | 80.33 |
| *Nnmt* | 11 | 114257787 | 114313536 | + | 11:114288368-114321792 | 11 | 114288368 | 114321792 | 31.94 | 31.36 | 45.25 | 28.59 | 107.03 | 115.69 | 34.90 | 56.12 | 54.32 |
| *Nudt6* | 4 | 122888697 | 122922968 | - | 4:122890031-122890396 | 4 | 122890031 | 122890396 | 0.00 | 8.28 | 0.00 | 7.15 | 74.07 | 26.78 | 4.77 | 22.86 | 41.42 |
| *Orm1* | 9 | 114323098 | 114326479 | + | 9:114327540-114332536 | 9 | 114327540 | 114332536 | 49.88 | 94.19 | 39.03 | 19.66 | 148.40 | 34.50 | 19.61 | 186.11 | 21.54 |
| *Pebp1* | 12 | 118136124 | 118145584 | + | 12:118129920-118144246 | 12 | 118129920 | 118144246 | 37.04 | 65.94 | 43.35 | 21.80 | 70.14 | 40.71 | 44.58 | 73.43 | 53.09 |
| *Phyhd1* | 9 | 128920966 | 128942041 | + | 9:128910653-128924320 | 9 | 128910653 | 128924320 | 31.41 | 42.69 | 26.58 | 25.94 | 101.75 | 63.73 | 38.11 | 65.75 | 47.42 |
| *Ppib* | 15 | 64155740 | 64163134 | - | 15:64157608-64157739 | 15 | 64157608 | 64157739 | 17.14 | 11.29 | 0.00 | 9.96 | 93.44 | 51.35 | 24.18 | 34.88 | 40.12 |
| *Proc* | 2 | 127418427 | 127429242 | + | 2:127404785-127420911 | 2 | 127404785 | 127420911 | 23.42 | 43.40 | 28.53 | 40.75 | 140.50 | 83.61 | 43.43 | 79.20 | 64.26 |
| *Psmg4* | 6 | 3231403 | 3303373 | + | 6:3281227-3282844 | 6 | 3281227 | 3282844 | 4.05 | 16.23 | 5.70 | 17.75 | 62.98 | 44.54 | 16.41 | 29.49 | 38.81 |
| *Pzp* | 12 | 9148840 | 9208395 | - | 12:9110290-9140285 | 12 | 9110290 | 9140285 | 41.47 | 27.77 | 29.29 | 32.83 | 136.04 | 93.53 | 48.86 | 72.74 | 62.73 |
| *Rarres1* | 3 | 158696892 | 158732489 | - | 3:158722154-158729389 | 3 | 158722154 | 158729389 | 6.28 | 11.74 | 9.98 | 17.86 | 67.66 | 84.74 | 42.61 | 62.17 | 63.73 |
| *Rbp4* | 10 | 93591687 | 93601744 | - | 10:93591372-93606555 | 10 | 93591372 | 93606555 | 66.61 | 74.98 | 54.05 | 15.84 | 67.90 | 29.20 | 30.14 | 56.83 | 49.57 |
| *Rbp7* | 1 | 9997206 | 10016021 | + | 1:9990208-9992042 | 1 | 9990208 | 9992042 | 9.50 | 10.73 | 19.62 | 18.31 | 39.05 | 47.20 | 28.78 | 33.65 | 48.46 |
| *Rcan1* | 21 | 34513142 | 34615113 | - | 21:34514913-34530407 | 21 | 34514913 | 34530407 | 51.77 | 37.42 | 38.83 | 34.49 | 162.69 | 108.05 | 46.80 | 64.40 | 54.84 |
| *Reep6* | 19 | 1491166 | 1497927 | + | 19:1489365-1494621 | 19 | 1489365 | 1494621 | 63.90 | 112.17 | 66.82 | 20.42 | 75.52 | 34.19 | 28.39 | 37.19 | 28.15 |
| *Rnaseh2c* | 11 | 65714005 | 65720818 | - | 11:65711154-65713239 | 11 | 65711154 | 65713239 | 120.60 | 212.59 | 145.18 | 65.76 | 174.67 | 146.08 | 36.57 | 38.20 | 30.18 |
| *Sdc2* | 8 | 96493813 | 96611790 | + | 8:96564775-96594224 | 8 | 96564775 | 96594224 | 7.89 | 11.84 | 8.87 | 22.19 | 106.30 | 55.83 | 61.08 | 91.57 | 86.80 |
| *Sdsl* | 12 | 113422380 | 113438276 | + | 12:113411831-113424817 | 12 | 113411831 | 113424817 | 21.54 | 33.66 | 30.17 | 28.15 | 162.02 | 48.84 | 41.52 | 51.24 | 47.48 |
| *Sec11c* | 18 | 59139866 | 59158832 | + | 18:59139470-59146426 | 18 | 59139470 | 59146426 | 50.17 | 86.09 | 68.40 | 10.08 | 55.21 | 32.76 | 37.08 | 102.39 | 68.09 |
| *Serpinf2* | 17 | 1742836 | 1755265 | + | 17:1761963-1766725 | 17 | 1761963 | 1766725 | 37.93 | 80.93 | 39.05 | 26.28 | 145.67 | 68.18 | 35.44 | 63.13 | 44.13 |
| *Slc17a4* | 6 | 25754699 | 25781199 | + | 6:25742363-25763119 | 6 | 25742363 | 25763119 | 28.31 | 35.91 | 37.62 | 19.02 | 75.79 | 76.06 | 45.22 | 76.49 | 75.78 |
| *Slc22a1* | 6 | 160121815 | 160158718 | + | 6:160119221-160144428 | 6 | 160119221 | 160144428 | 18.58 | 38.87 | 13.93 | 52.28 | 215.80 | 74.26 | 39.79 | 51.30 | 50.12 |
| *Slc25a20* | 3 | 48856926 | 48898904 | - | 3:48901310-48901463 | 3 | 48901310 | 48901463 | 6.08 | 10.72 | 11.28 | 24.58 | 78.36 | 14.40 | 18.84 | 46.47 | 22.33 |
| *Slc25a25* | 9 | 128068201 | 128109245 | + | 9:128060206-128106789 | 9 | 128060206 | 128106789 | 25.27 | 41.47 | 31.90 | 24.60 | 101.94 | 74.10 | 46.21 | 77.33 | 64.14 |
| *Slc25a47* | 14 | 100323339 | 100330421 | + | 14:100318144-100318355 | 14 | 100318144 | 100318355 | 57.78 | 90.09 | 147.01 | 24.73 | 139.98 | 54.90 | 5.82 | 59.28 | 34.21 |
| *Slc40a1* | 2 | 189560590 | 189583758 | - | 2:189547841-189585643 | 2 | 189547841 | 189585643 | 28.66 | 38.33 | 33.59 | 31.33 | 106.36 | 113.74 | 78.93 | 133.68 | 120.45 |
| *Soat2* | 12 | 53103486 | 53124535 | + | 12:53093738-53094715 | 12 | 53093738 | 53094715 | 7.49 | 20.61 | 12.63 | 22.93 | 93.75 | 74.27 | 37.47 | 36.26 | 34.77 |
| *Spp2* | 2 | 234050679 | 234077134 | + | 2:234081194-234085127 | 2 | 234081194 | 234085127 | 17.41 | 11.96 | 20.08 | 20.90 | 92.61 | 84.87 | 47.58 | 77.09 | 64.21 |
| *Stab2* | 12 | 103587273 | 103766719 | + | 12:103596328-103596661 | 12 | 103596328 | 103596661 | 4.91 | 47.33 | 0.00 | 31.34 | 54.61 | 46.31 | 13.11 | 21.67 | 22.73 |
| *Stard5* | 15 | 81309053 | 81324183 | - | 15:81290727-81305835 | 15 | 81290727 | 81305835 | 16.20 | 52.17 | 43.42 | 29.94 | 116.27 | 93.95 | 28.08 | 66.96 | 54.76 |
| *Tat* | 16 | 71565660 | 71577092 | - | 16:71563289-71588891 | 16 | 71563289 | 71588891 | 29.89 | 32.40 | 33.01 | 43.24 | 232.35 | 94.78 | 38.01 | 54.20 | 54.56 |
| *Tdo2* | 4 | 155854738 | 155920406 | + | 4:155878571-155891364 | 4 | 155878571 | 155891364 | 11.17 | 14.47 | 10.38 | 30.05 | 177.63 | 108.31 | 61.61 | 91.48 | 84.05 |
| *Timm9* | 14 | 58408495 | 58427531 | - | 14:58423882-58430571 | 14 | 58423882 | 58430571 | 80.40 | 104.92 | 108.66 | 31.27 | 131.63 | 108.65 | 38.64 | 68.56 | 64.79 |
| *Tmem176a* | 7 | 150800403 | 150805118 | + | 7:150775602-150791739 | 7 | 150775602 | 150791739 | 13.99 | 15.80 | 15.34 | 40.35 | 157.16 | 102.71 | 63.01 | 78.83 | 71.54 |
| *Tmem82* | 1 | 15742499 | 15747982 | + | 1:15734927-15737925 | 1 | 15734927 | 15737925 | 37.03 | 56.30 | 27.85 | 18.03 | 31.53 | 12.24 | 47.17 | 53.21 | 32.28 |
| *Tst* | 22 | 37010859 | 37020183 | - | 22:37010870-37046225 | 22 | 37010870 | 37046225 | 38.83 | 51.71 | 34.46 | 16.31 | 66.97 | 32.71 | 55.15 | 72.28 | 54.66 |
| *Ttc32* | 2 | 19896631 | 19901983 | - | 2:19892414-19903186 | 2 | 19892414 | 19903186 | 54.05 | 84.53 | 78.18 | 32.96 | 120.58 | 108.11 | 57.48 | 124.56 | 120.14 |
| *Ugp2* | 2 | 63840952 | 63891562 | + | 2:63837371-63858664 | 2 | 63837371 | 63858664 | 60.25 | 82.54 | 65.81 | 38.65 | 132.48 | 119.98 | 51.71 | 109.65 | 103.93 |
